# Breaking of the site-bond percolation universality in networks

**DOI:** 10.1038/ncomms10196

**Published:** 2015-12-15

**Authors:** Filippo Radicchi, Claudio Castellano

**Affiliations:** 1 Center for Complex Networks and Systems Research, School of Informatics and Computing, Indiana University, 919 E. 10^th^ St, Bloomington, Indiana 47408, USA; 2 Istituto dei Sistemi Complessi (ISC-CNR), Via dei Taurini 19, Roma 00185, Italy; 3 Dipartimento di Fisica, Sapienza Università di Roma, P.le A. Moro 2, Roma 00185, Italy

## Abstract

The stochastic addition of either vertices or connections in a network leads to the observation of the percolation transition, a structural change with the appearance of a connected component encompassing a finite fraction of the system. Percolation has always been regarded as a substrate-dependent but model-independent process, in the sense that the critical exponents of the transition are determined by the geometry of the system, but they are identical for the bond and site percolation models. Here, we report a violation of such assumption. We provide analytical and numerical evidence of a difference in the values of the critical exponents between the bond and site percolation models in networks with null percolation thresholds, such as scale-free graphs with diverging second moment of the degree distribution. We discuss possible implications of our results in real networks, and provide additional insights on the anomalous nature of the percolation transition with null threshold.

## Abstract (web summary)

The percolation transition has been regarded as model-independent, namely determined by the geometry of a system but otherwise identical for bond or site percolation models. Here, the authors show the violation of this assumption both analytically and numerically for networks with null percolation thresholds.

## Graphical abstract

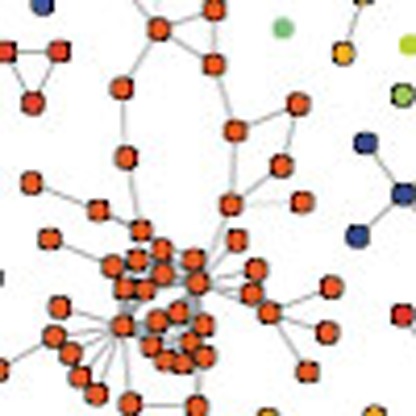

Percolation is among the simplest processes able to generate continuous phase transitions^1^,^2^. The model used to describe percolation assumes the presence of an underlying network structure where either nodes (site percolation) or edges (bond percolation) are randomly occupied with probability *p*. Nearest-neighbor occupied elements form connected clusters. In site percolation, for *p*=0, no elements are present in the system, so that all clusters have size zero. In bond percolation, for *p*=0, no nodes are connected in the system, so that all clusters have size equal to one. In both models, for *p*=1, only a single cluster, coinciding with the whole network, is present. The term percolation transition refers to the structural change, between these two extreme configurations, observed as a function of the occupation probability *p*. The change is usually monitored through the relative size of the largest cluster, or percolation strength, which is regarded as the order parameter of the percolation transition. In the limit of infinitely large networks, this observable is always equal to zero for any value of *p*≤*p*_c_, while it is finite for *p*>*p*_c_. Whereas the percolation threshold *p*_c_ can be different in the two models, for a fixed underlying network, bond and site percolation processes have been always observed to behave identically around their respective threshold values. The exponent describing the power-law growth of the order parameter as a function of the distance from the critical point is the same in both processes^1^. This statement is true also for the critical exponents that describe the singular behavior of other observables, such as the distribution of the cluster size, and the average size of finite clusters. The specific values of the critical exponents play an important role in the characterization of the properties of the percolation transition, and they are used to group networks in different universality classes. In lattices for example, the values of the critical exponents depend only on the dimensionality of the euclidean space^1^. Such a dependence disappears above the upper-critical dimension, where the critical exponents stabilize to their mean-field values^1^. In random networks also, no differences have been reported between the critical exponents of the bond and site percolation models^3^,^4^,^5^,^6^. Theoretical approaches proposed so far indeed assume a perfect equivalence between the models^6^.

 In this paper we are going to show that this assumption is incorrect. In graphs with null percolation threshold, as for example random networks with diverging second moment of the degree distribution, bond and site percolation strengths are characterized by different critical exponents. The breaking of the site-bond universality is accompanied with anomalies in the critical behavior of other macroscopic observables.

## Results

### Bond percolation model

We first derive the basic equations that support our statement, starting from the bond percolation model. We assume the presence of an underlying undirected and unweighted network composed of *N* nodes and *E* edges. The structure of the network is fully described by the adjacency matrix *A*. The generic element of this matrix equals one if the two corresponding nodes share an edge, whereas equals zero if no connection is present between the two vertices. The probability *b*_*i*_ that node *i* is part of the largest cluster of the network is a function of *A* and the bond occupation probability *p*. Such a probability obeys the equation


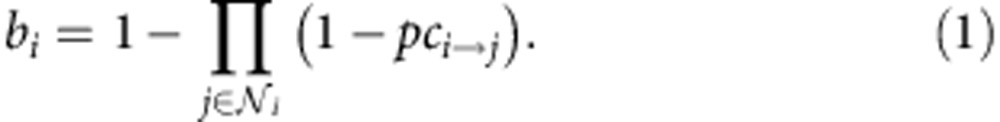


Here, 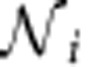 is the set of neighbors of vertex *i*, while *c*_*i*→*j*_ stands for the probability that node *j* is part of the largest cluster discounting the contribution of node *i*. Equation (1) is formulated according to the following straightforward argument. If node *j* is in the set 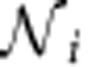 of neighbors of vertex *i*, then *pc*_*i*→*j*_ is the probability that the connection between *i* and *j* is occupied, and node *j* is part of the spanning cluster thanks to a node different from *i*. Thus, the probability that node *i* does not belong to the largest cluster, i.e., 1−*b*_*i*_, is equal to the probability that none of its adjacent nodes, that are connected to vertex *i* by an occupied edge, are part of the largest cluster of the graph. Note that equation (1) is based on the hypothesis that the probabilities *c*_*i*→*j*_ of all neighbors of node *i* are uncoupled, i.e., the so-called locally tree-like approximation^6^, hence their product appears on the r.h.s. of the equation. For consistency, the probability *c*_*i*→*j*_ obeys


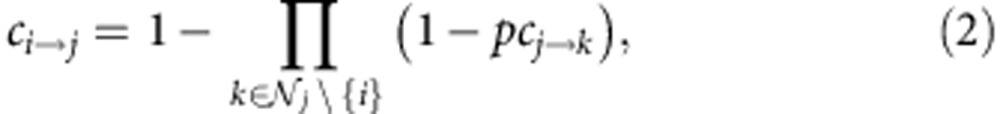


where the product on the r.h.s. of the last equation runs over all neighbors of node *j* but vertex *i*. Given the adjacency matrix *A* of the underlying graph, and fixed a value of the occupation probability *p*, the solution of the bond percolation model can be obtained first by numerically solving the set of 2*E* equations (2), and then plugging these solutions into the set of *N* equations (1) to estimate the value of the variables *b*_*i*_. The order parameter of the transition can be finally computed as the average value of these variables over the entire network, i.e., 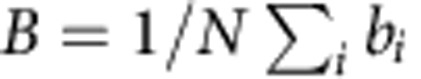. This quantity represents the percolation strength *B* over an infinite number of realizations of the bond percolation model on the graph. Using the Taylor expansion of equation (2) around *c*_*i*→*j*_=0, it can be shown that the percolation threshold equals the inverse of the largest eigenvalue of the non-backtracking matrix of the graph^7^, and that slightly on the right of the critical probability, every *b*_*i*_ grows linearly with the sum of the components of the principal eigenvector of the non-backtracking matrix corresponding to edges pointing out from node *i*^8^.

### Site percolation model

Under the locally tree-like approximation, the probability *s*_*i*_ that node *i* belongs to the largest cluster in the network is given by


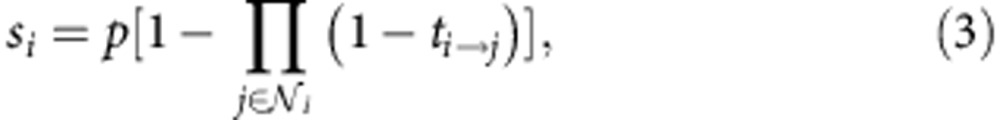


where *t*_*i*→*j*_ stands for the probability that node *j* is part of the largest cluster irrespective of vertex *i*. The probability *s*_*i*_ is written as the product of two contributions: the probability *p* that the node is occupied, and the probability that at least one of its neighbors is part of the largest cluster independently of node *i*. For consistency, the probability *t*_*i*→*j*_ obeys


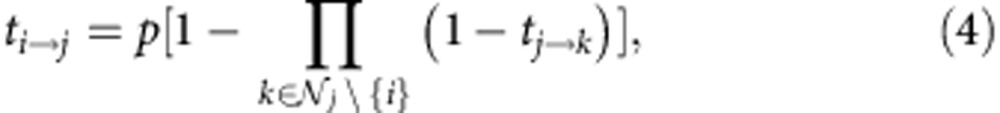


where we have excluded node *i* from the product on the r.h.s. As in the case of bond percolation, equations (4) form a set of 2*E* coupled equations whose solution can be obtained numerically for any value of *p*. The numerical solutions of equation (4) are then plugged into equation (3) to obtain the values of the variables *s*, and finally the order parameter of the transition is computed as 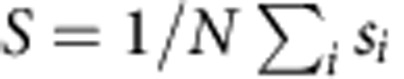. Also in this case, the percolation threshold equals the inverse of the largest eigenvalue of the non-backtracking matrix of the graph^9^.

### Relation between bond and site percolation

**Figure 1:**
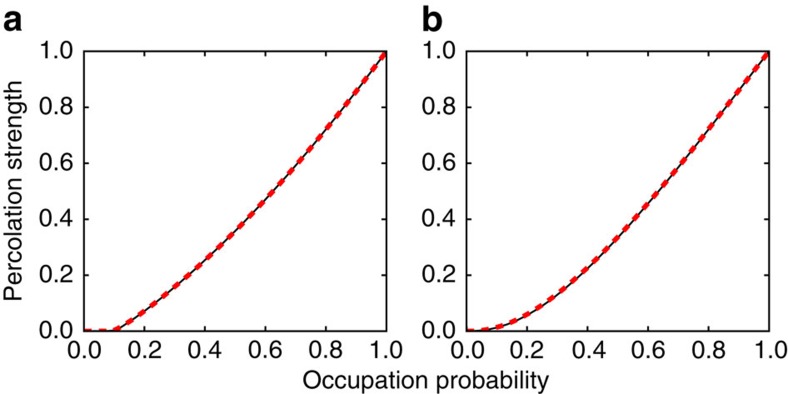
Percolation diagrams of real networks. (**a**) We consider the graph corresponding to the giant component of the *peer-to-peer Gnutella* network as of August 31, 2002 (refs 32, 33). The black thin line represents the site percolation order parameter *S* as a function of the site occupation probability *p*. We calculate also the order parameter *B* for bond percolation and multiply it by *p* to obtain the red dashed line. The average clustering coefficient of the network is *C*=0.0055. Such a low value indicates that the tree-like approximation holds with sufficient accuracy for this network. We further estimated the error *V* of the law *S*=*pB*, by considering the integral 
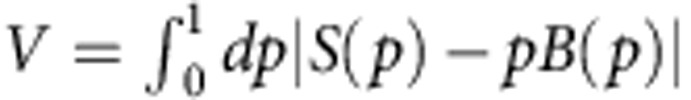
. We find *V*=0.0002. (**b**) We consider the graph corresponding to the giant component of the *Internet at the autonomous system level* in the period January 2004 to November 2007 (ref. 34). The description of the various curves is identical to those appearing in panel **a**. The clustering coefficient for this network is *C*=0.2082. The error associated to equation (5) is *V*=0.0016.

If we multiply both sides of equation (2) by *p*, we recover equation (4), with only the necessity of renaming *pc*_*i*→*j*_→*t*_*i*→*j*_. The same is also true for equation (1) which reduces to equation (3) with a multiplication by *p*, and the additional change of variable *pb*_*i*_→*s*_*i*_. As a consequence, the percolation strengths *B* and *S* are related by


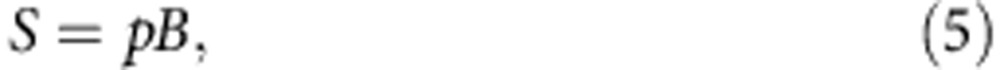


which tells us that, in locally tree-like networks, the order parameters of the bond and site percolation models are linearly proportional^10^. Equation (5) holds with very high accuracy in many real networks, as long as their structure is sufficiently compatible with the locally tree-like approximation (see Fig. 1, Supplementary Figs 1-109, Supplementary Tables 1-3, and Supplementary Note 1 for results on 109 real networks^11^). To provide a quantitative test of this statement, we estimate the error associated to equation (5) as 
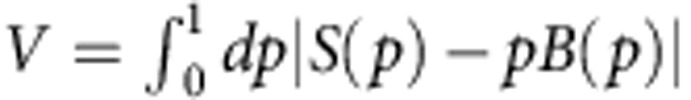
, and use the average clustering coefficient *C* as a proxy for the validity of the tree-like ansatz. We find *V*<0.1 for all the real networks we analyzed (see Supplementary Tables 1-3), suggesting a good accuracy of equation (5) overall. For most networks with relatively low values of the clustering coefficient, equation (5) works exceptionally well (i.e., *V*<0.01). On the other hand, we find also a positive dependence of *V* on *C*, indicating that the accuracy of equation (5) decreases as the tree-like approximation becomes less reliable (see Supplementary Fig. 110).

### Violation of the site-bond percolation universality

From equation (5) a difference in the critical behavior between the bond and site percolation models is straightforwardly deduced. In infinitely large networks, as the occupation probability tends to the critical threshold value from right, i.e., 
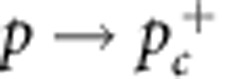
, the order parameter of the percolation transition decreases to zero as a power of the distance from the critical point, that is 
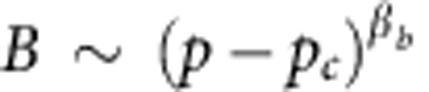
 and 
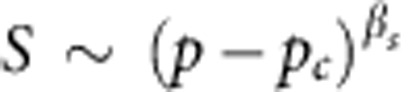
. Whereas in the former equations we stressed the possibility of a difference in the values of the critical exponents for the bond and site percolation models, we remark that there are not known examples of such observation. On the contrary, it is firmly believed that the value of critical exponents depends only on the geometry of the system but not on the specific ordinary percolation model considered^1^. By making use of the linear mapping of equation (5), we can write 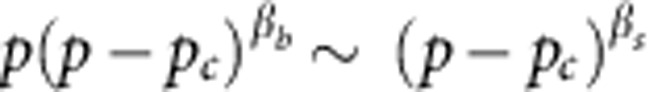. If the percolation threshold is strictly larger than zero, as in the case of regular graphs, Erdös-Rényi models, or random scale-free graphs with finite second moment of the degree distribution, in the limit 
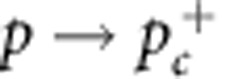
, the prefactor *p* on the l.h.s. of the previous equation acts as a multiplicative constant, and *β*_b_=*β*_s_. If instead *p*_c_=0, as in the case of random scale-free graphs with diverging second moment of the degree distribution^3^,^12^,^13^, the former equation becomes 
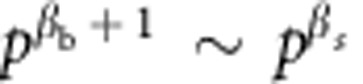
. The critical exponents of the percolation strengths of bond and site percolation are thus related by


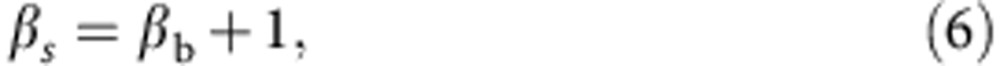


which tells us that, in locally tree-like graphs with null percolation thresholds, the site-bond universality is broken, and the critical exponents of the order parameters of the bond and site percolation models assume different values.

**Figure 2:**
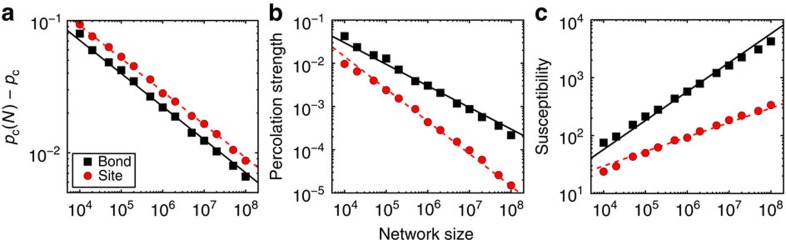
Critical behavior of bond and site percolation on scale-free graphs. Results are obtained for random networks with degree distribution *P*(*k*)∼*k*^−*γ*^ built according to the uncorrelated configuration model (see methods) and setting the degree exponent *γ*=2.5. Black squares refer to bond percolation, while red circles represent the results obtained for site percolation. (**a**) Best estimate of the pseudo-critical point *p*_c_(*N*) for different network sizes *N*. Simulation results (symbols) are compared with the expected power-law decay (full black and dashed red lines are guides to the eye) towards *p*_c_, with *p*_c_=0 and decay exponent 1/*ν*=(3−*γ*)/2. (**b**) Percolation strengths *B* (black squares) and *S* (red circles) at *p*=*p*_c_(*N*) as functions of the network size *N*. The full black line serves as a guide to the eye and decays with exponent *β*_b_/*ν*=1/2 as *N* grows (see methods). The dashed red line serves as a guide to the eye to indicate a power-law decay with an exponent *β*_s_/*ν*=(4−*γ*)/2 (see methods). (**c**) Maximal values of susceptibilities *χ*_B_ (black squares) and *χ*_S_ (red circles) as functions of *N*. The full black line increases as 
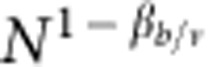
, thus as *N*^1/2^. The dashed red line stands for guide to the eye for the power-law divergence 
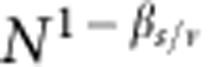
, which means *N*^(*γ*−2)/2^.

**Figure 3:**
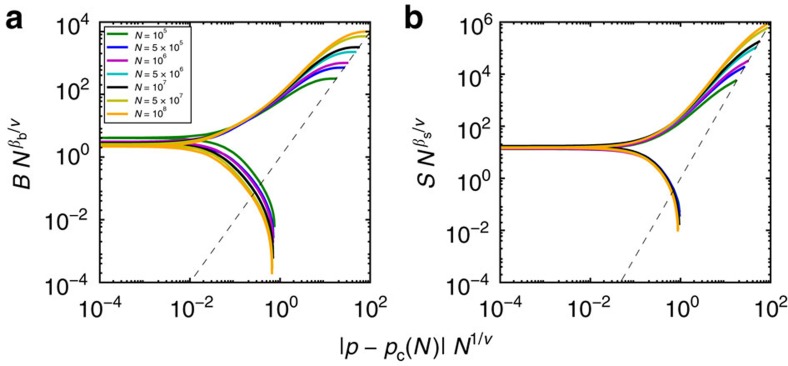
Finite-size scaling in scale-free graphs. We analyze the same networks as in Fig. 2, and test the validity of equation (13). (**a**) Collapse plot for the order parameter *B* of the bond percolation model in networks with different sizes. The collapse is obtained by setting *β*_b_/*ν*=1/2 and *ν*=2/(3−*γ*). The dashed line corresponds to a guide to the eye for a power-law with exponent equal to *β*_b_=1/(3−*γ*). The imperfect collapse for 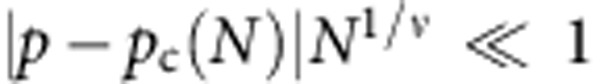 is a consequence of the effective exponent *β*_b_/*ν* in Fig. 2b, which is slightly larger than the value 1/2 due to preasymptotic effects. (**b**) Same as in panel **a**, but for the site percolation model. In this case, we set *β*_s_/*ν*=(4−*γ*)/2 and *ν*=2/(3−*γ*). The dashed line is a guide to the eye for a power-law with exponent equal to *β*_s_=(4−*γ*)/(3−*γ*).

To validate our theoretical predictions, we numerically study the two percolation models in random graphs^14^,^15^ using the Monte Carlo algorithm introduced by Newman and Ziff^16^. We consider random network models that are sparse enough to satisfy the locally tree-like ansatz, and extrapolate critical exponent values of the transition for networks of infinite size by making use of finite-size scaling analysis^1^. First, we verify that for random graphs with nonvanishing percolation thresholds identical values for the critical exponents in bond and site percolation are indeed recovered (Supplementary Figs 111-118). In particular, for networks with power-law degree distribution but finite second moment, we obtain values of the critical exponents consistent with previous theoretical predictions^5^,^17^. These statements are valid not just for the critical exponent *β*, but also for the one that regulates the divergence of the average cluster size, as well as for the Fisher exponent of the distribution of cluster sizes at criticality^1^. Results for scale-free graphs with diverging second moment of the degree distribution, and thus null percolation thresholds, are reported in Fig. 2. For the bond percolation model, we recover the value of the exponent *β*_b_ predicted by the theory of Cohen *et al.*^5^ For the site percolation model, we find instead results consistent with our equation (6) (see also refs 6, 18). These different values of the critical exponents are confirmed in Fig. 3 by the good scaling collapse among curves corresponding to different network sizes.

### Interpretation of universality breaking

What is the physical reason of the difference between the exponents *β* in the two percolation models? To get insights, consider a star-like graph, where a single node is connected to an infinitely large number of vertices. This structure represents the extreme limit of a network with diverging second moment of the degree distribution, and it is often used to understand basic mechanisms induced by the heterogeneity of the node degrees^6^. In the bond percolation model, every node at the end of an active edge is automatically part of the largest cluster. An increment in the occupation probability *p* generates a linear increment of the relative size of the largest cluster, that is *B*∼*p*. In the site percolation model instead, the largest cluster can grow only if the center of the star is active. This happens with probability *p*. If the center of the star is active, then the growth of the largest cluster is determined by the total number of other vertices that are active, that is the rate of growth of the largest cluster in the bond percolation model. Thus, the relative size of the largest cluster in the site percolation model behaves as *S*∼*p*^2^, in accordance with equation (6).

We expect the same physical principle to play a fundamental role in percolation processes on random graphs with degree distribution *P*(*k*). The giant connected component, near its point of creation, has degree distribution proportional to *kP*(*k*), hence it consists mostly of vertices with high degree or hubs^6^ (Supplementary Fig. 119). Bond and site percolation models differ, however, in the way nodes with different degree become part of connected clusters. In the bond percolation model, there is a preference for selecting edges attached to hubs, as *kP*(*k*) is the probability that a node at the end of a randomly selected edge has degree equal to *k*. In the site percolation model instead, a node with degree *k* is activated with probability *P*(*k*) so that there is a weaker preference to select hubs. However, when a high-degree vertex is activated, many edges are activated simultaneously, and many clusters can be merged together. Such a microscopic difference among the two models becomes apparent, with different values of the critical exponent *β*, only if the number of hubs is sufficiently large, as for example in scale-free graphs with *P*(*k*)∼*k*^−*γ*^ and degree exponent 2<*γ*<3. For *γ*>3 instead, hubs are too rare to generate differences at the macroscopic level, and the site-bond percolation universality is restored.

### Anomalies of percolation in scale-free graphs

**Figure 4:**
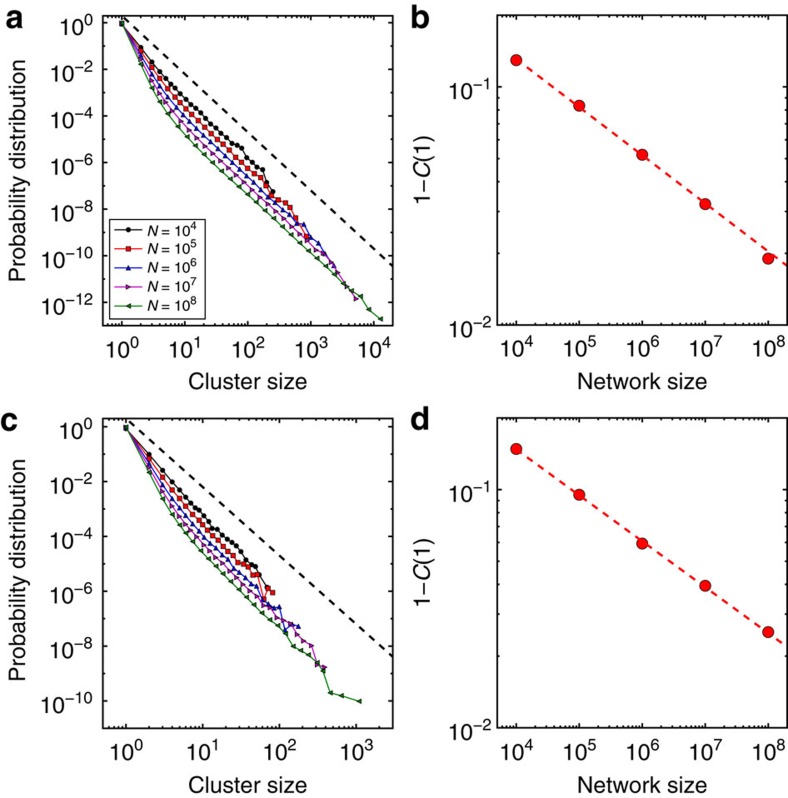
Distribution of finite-cluster sizes in scale-free graphs. We analyze the same networks as in Figs 2 and 3. (**a**) Probability distribution to observe a cluster of a given size in the bond percolation model for *p*=*p*_c_(*N*). Each curve corresponds to a different network size. The tail of the various distributions decays as a power-law for large values of the cluster sizes with exponent compatible with 5/2 (the dashed line is a guide to the eye) (**b**) Weight of clusters of size one, *C*(1), in the distribution of finite-cluster sizes. As the system size grows, *C*(1) tends to one in a power-law fashion (the red dashed line represents the best power-law fit of the empirical points and has decay exponent equal to 0.20). (**c**,**d**) Same as in panels (**a**,**b**) but for the site percolation model. In panel (**c**) the black dashed line serves as a guide to the eye for a power-law decay with exponent 5/2. In panel (**d**) the red dashed line represents the best power-law fit of the empirical points and has decay exponent equal to 0.18.

The study of other macroscopic observables reveals that random networks with null percolation thresholds show anomalies not just at the level of the critical exponents of the order parameter, but in the nature of the transition itself. In ‘standard' percolation transitions, the distribution of finite cluster sizes decreases at criticality as a power-law with an exponential cut-off diverging as the system becomes infinite^1^. In scale-free graphs with null percolation threshold the power-law decay is only a preasymptotic effect, visible only in finite-size systems. This is clearly seen in Fig. 4a,c, showing a power-law tail which tends to disappear in the limit of infinitely large networks. The vanishing of the power-law tail is confirmed in Fig. 4b,d, showing that all the distribution weight gets concentrated on clusters of size 1. This finding is in stark contrast with all theoretical predictions proposed so far^5^,^6^,^19^,^20^, which are inconsistent with each other, as they all provide different estimates for the Fisher critical exponent. We emphasize that their validity has been never systematically tested in numerical experiments. Our results can be interpreted by intuitive arguments. If the percolation threshold is zero, then the critical configuration is given by a disconnected network where all clusters have size one in bond percolation, and size zero in site percolation. Analogous considerations about the critical configuration have been deduced for self-similar graphs^21^, although no difference between site and bond percolation was studied. As a matter of fact, the Fisher critical exponent is not clearly defined, because the entire cluster size distribution does not decay as a power-law. The same argument implies also that the average size of finite clusters does not diverge at criticality and its associated critical exponent is equal to zero (Supplementary Figs 120 and 121).

## Discussion

The breakdown of site-bond percolation universality in locally tree-like networks with null thresholds is a surprising result. Although percolation processes have been extensively studied in the last decades, to the best of our knowledge, there are no previous findings of such discrepancy between the bond and site percolation models. A relation analogous to equation (6) has been found long ago in continuum percolation models for conductivity in *d*-dimensional porous rocks^22^. We stress however that the similarity is only formal, as here the relation is between standard bond and site percolation, while (ref. 22) connects the *β* exponents of an ordinary and a suitably modified continuum percolation process in *d*-dimensional spaces. Our results could therefore contribute to percolation theory by stimulating further research in a direction not yet explored. Also, we remark that scale-free graphs with diverging second moments of the degree distribution are regarded as prototypical models of a large variety of natural and man-made networks^19^,^23^. In this context, our results could have direct consequences in all situations where percolation plays a fundamental role, including spreading processes in networks^24^,^25^,^26^, as well as resilience properties of graphs to random breakdowns^12^,^13^,^27^. One may remark that many real networks are characterized by high values of the clustering coefficient^28^, and thus violate the tree-like approximation at the basis of our mathematical framework. We argue that a nonvanishing clustering coefficient is not a sufficient ingredient to restore the percolation universality class in networks with diverging second moment of the degree distribution. By repeating our numerical experiments on the generalization of the configuration model proposed by Newman^29^, that creates random scale-free networks with nonvanishing clustering coefficients, we find in fact that the anomalous phenomenology still persists (Supplementary Fig. 122). Other ingredients seem thus necessary to observe a nonvanishing percolation threshold and consequently to restore the percolation universality class in networks with diverging second moment of the degree distribution. For instance, we expect that scale-free network models characterized by spatial embedding^30^ or high density of cliques^31^ will not exhibit such an anomalous behavior.

## Methods

### Order parameters and critical exponents

The main order parameter used in the study of the percolation transition in networks is the so-called percolation strength, defined as the number of nodes belonging to the largest connected cluster of the network divided by the total number of vertices in the graph. In our paper, we indicated this quantity as *B* for bond percolation, and *S* for site percolation. In the limit of infinitely large systems, the order parameter *B* grows as a power-law function of the distance between the actual value of the occupation probability *p* and the critical threshold *p*_*c*_, that is


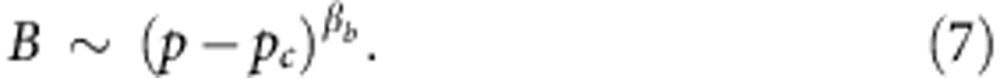


The same behavior is valid for *S*, and the critical exponent is denoted as *β*_s_. As already explained in the text, the value of the critical exponents *β*_b_ and *β*_s_ is the same if the percolation threshold *p_c_* is strictly larger than zero. Whereas *B* and *S* are based only on the size of the largest connected cluster in the network, there are other important macroscopic observables that account for the size of the other clusters, and critical exponents that are associated with them. In our paper, we considered the distribution of the cluster size at criticality which leads to the definition of the Fisher exponent *τ*, and the average size of finite clusters with associated critical exponent *ω*.

### Numerical simulations

Given an undirected and unweighted network with *N* nodes and *E* edges composed of a single connected component, we study bond percolation using the Monte Carlo method proposed by Newman and Ziff^16^. In each realization of the method, we start from a configuration with no connections. We then sequentially add edges in random order and monitor the evolution of the size of the largest cluster in the network *Z*(*p*) as a function of the bond occupation probability *p*=*e*/*E*, where *e* indicates the number of edges added from the initial configuration, i.e., *e*=0. We repeat the entire process *Q* independent times and estimate the order parameter *B* as


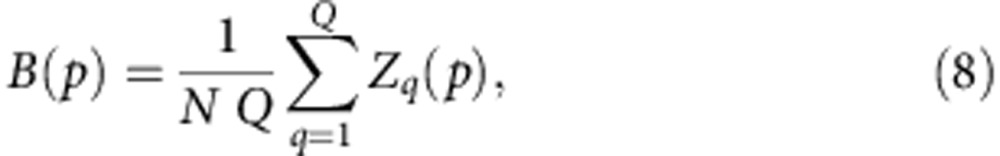


where *Z*_*q*_(*p*) indicates the size of the largest cluster in the network observed, during the *q*th realization of the Monte Carlo algorithm, when the bond occupation probability equals *p*. The susceptibility *χ*_B_ is instead evaluated as


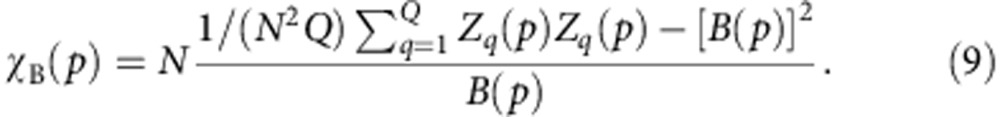


The numerical value of *p*_c_(*N*) is given by the value of *p* for which *χ*_B_ is maximum. In our simulations, we also keep track of the size *z* of all other clusters present in the network, and monitor the average size of finite clusters 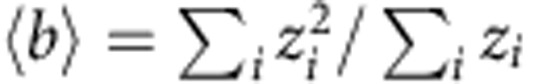, where the sum runs over all clusters excluding the largest one. Results shown in the paper are obtained by considering *Q*=10,000 in simulations of the percolation process in real networks (Fig. 1), and *Q*=1,000 (Figs 2 and 3) or *Q*=100 (Fig. 4) in artificial graphs.

Simulations for the site percolation model are performed in a similar way as described above for the bond percolation model. The initial configuration is given by a network with no nodes, i.e., *n*=0. Vertices are then sequentially introduced in the network in a random order. The occupation probability is defined as *p*=*n*/*N*, with *n* number of nodes added in the Monte Carlo algorithm. The definitions of the order parameter, the susceptibility and the average cluster size are identical to those of the bond percolation model. These quantities are respectively denoted as *S*, *χ*_S_ and 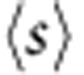.

### Random networks

The generation of a single instance of the Erdös-Rényi model with *N* nodes and average degree 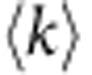 is obtained by connecting each pair of nodes with probability 
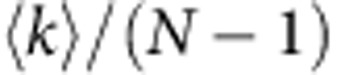
.

To generate a random network with *N* nodes and power-law degree distribution


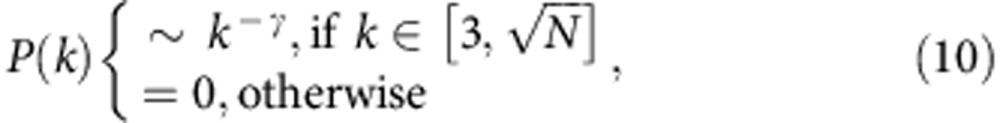


we make use of the so-called uncorrelated configuration model^14^,^15^. The support of the degree distribution is chosen in such a way that the resulting network has no degree-degree correlations, and is always composed of a single connected component. In the generation of a single instance of the network model, we first assign degrees to the nodes according to the prescribed *P*(*k*). Then, we attach pairs of nodes at random, preserving their pre-imposed degrees, but not allowing for multiple connections and self-loops.

To generate a random network with *N* nodes and nonvanishing clustering coefficient, we make use of the generalization of the uncorrelated configuration model proposed by Newman^29^. We first assign each node to a number of triangles randomly extracted from the power-law distribution


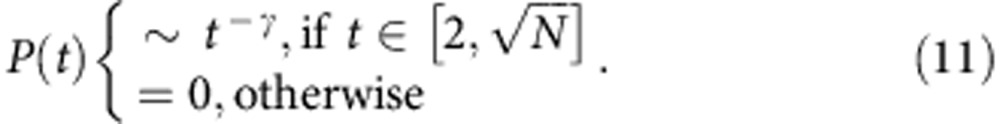


The support of the distribution is chosen in such a way that the resulting network has no degree-degree correlations, and is always composed of a single connected component. After each node has assigned a number *t*, we then attach triplets of nodes at random, preserving their pre-imposed number of triangles *t*, but not allowing for multiple connections and self-loops. The procedure generates a graph with power-law degree distribution with degree exponent *γ*, and average clustering coefficient 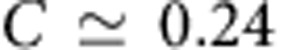 for all sizes *N*. The clustering coefficient *C* of the network is defined as the average value of the clustering coefficients of all the nodes in the graph. The clustering coefficient *C*_*i*_ for node *i* is defined as


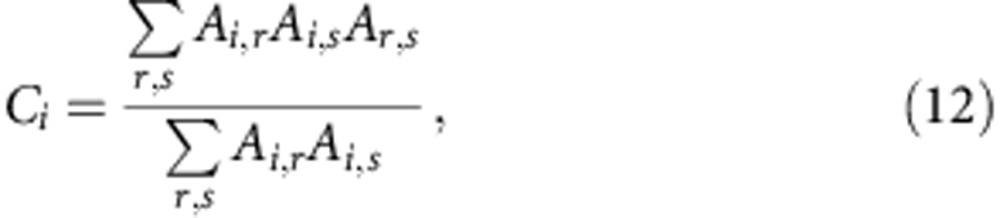


with *A*_*i*,*j*_=1 if nodes *i* and *j* are connected, and *A*_*i*,*j*_=0, otherwise.

Results appearing in the paper are obtained on single network instances.

### Finite-size scaling analysis

On a finite network of size *N*, the order parameter *B* follows the scaling


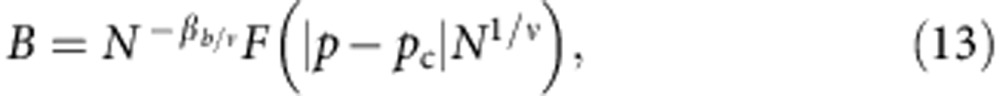


where *β*_b_ is the critical exponent that regulates the power-law behavior of *B* in the infinite-size limit, *ν* is the critical exponent associated with the correlation length of the system, and *F* is a scaling function. The exponent *ν* can be determined by monitoring how the pseudo-critical threshold *p*_c_(*N*) changes as a function of the network size. This quantity is determined by looking at the location of the peak of the susceptibility *χ*_B_. The pseudo-critical threshold decays towards the percolation threshold *p*_c_ as


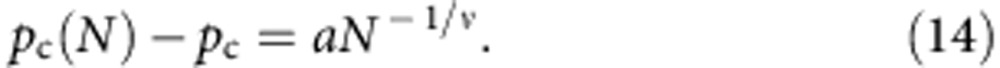


If one measures the value of the order parameter *B* at *p*=*p*_c_(*N*), the argument of the universal function does not longer contain any dependence on either *N* and *p*, so that 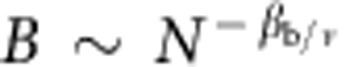, and the ratio of the critical exponents *β*_b_ and *ν* can be determined from the decay of the order parameter *B* for different network sizes. By definition, the susceptibility *χ*_B_ diverges at pseudo-criticality as 
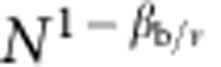
. The same exact technique can be also used to determine the power-law scaling of the average cluster size 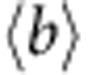. In the case of standard percolation transitions, the average cluster size is expected to diverge at (pseudo-) criticality as 
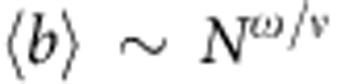
. Critical exponents for the site percolation model are numerically determined in the same way as described above.

### Percolation thresholds and critical exponents

For a finite random network obeying the locally tree-like ansatz, and with degree distribution *P*(*k*), the pseudo-critical percolation threshold is determined as


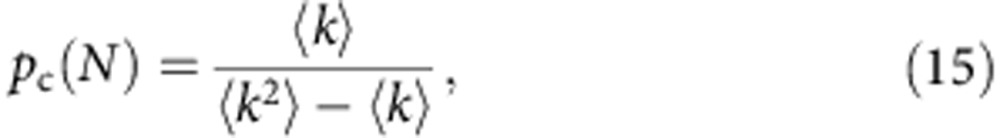


with 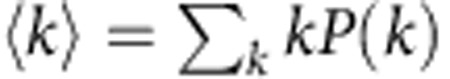 and 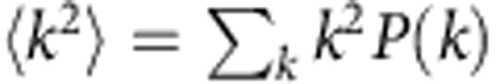 respectively equal to the first and second moments of the degree distribution *P*(*k*)^3^,^13^. This expression is computed with the so-called heterogeneous mean-field theory. It allows us to determine the percolation threshold *p*_c_ for networks with infinite sizes, and also the value of the critical exponent *ν* depending on how *p*_c_(*N*) approaches *p*_c_ as *N* grows. If the degree distribution is given by equation (10), then we have 
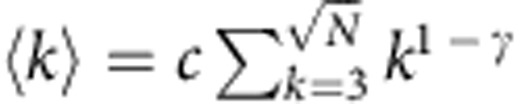
 and 
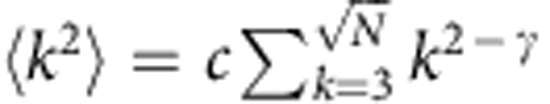
, with *c* normalization constant. We have therefore different predictions based on the value of γ, i.e., depending on whether the second moment of the distribution is diverging or not as *N* increases. For the percolation threshold, we have


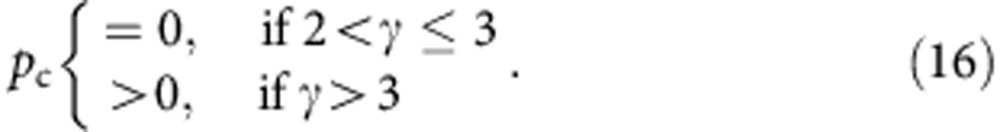


For the critical exponent *ν*, we instead have


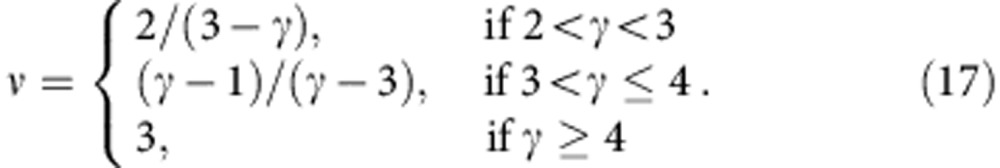


*γ*=3 is a pathological case where we do not expect a power-law decay of *p*_c_(*N*) to *p*_c_, but rather an exponential one. The prediction in the regime 2<*γ*<3 is obtained by accounting for the divergence of the second moment of the degree distribution with cutoff given by 
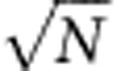
. The prediction in the regime 3<*γ*≤4 has been obtained by Wu *et al.*^17^ For *γ*≥4 instead, the exponent *ν* equals its mean-field value.

The estimates of the critical exponent *β* for the percolation strength are instead given by


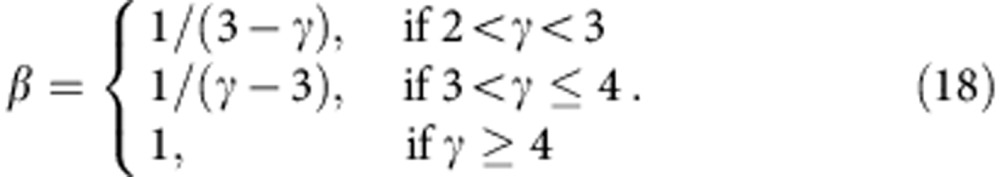


These predictions have been obtained by Cohen *et al.*^5^ In the regime *γ*≥4, *β* assumes its mean-field value. The results of our simulations show the prediction in the regime 2<*γ*<3 to be valid only for the bond percolation model, i.e., *β*_b_=1/(3−*γ*). For the site percolation model, we have instead *β*_s_=*β*_b_+1=(4−*γ*)/(3−*γ*).

According to our arguments, the exponents *τ* and *ω*, respectively used to characterize the distribution of cluster sizes and the average cluster size, are not defined in the regime 2<*γ*<3, where these quantities do not obey power-law scalings. They are instead well defined for *γ*>3, where Cohen *et al.*^5^ predicted


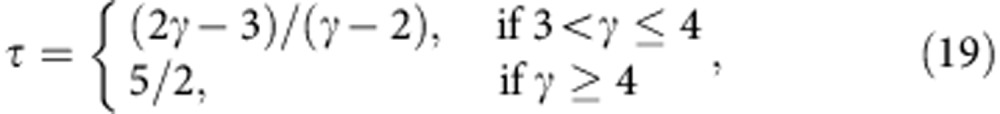


and


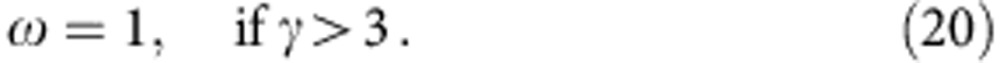


Again, the values of the critical exponents for *γ*>4 are given by their mean-field expectations. We stress also that the critical exponents are related by precise hyperscaling relationships. For example, we must have 2*β*/*ν*+*ω*/*ν*=1.

## Additional information

**How to cite this article:** Radicchi, F. & Castellano, C. Breaking of the site-bond percolation universality in networks. *Nat. Commun.* 6:10196 doi: 10.1038/ncomms10196 (2015).

## Supplementary Material

Supplementary Information Supplementary Figures 1-122, Supplementary Tables 1-3, Supplementary Note 1 and Supplementary References.
