# Supplementary material for: Breaking of the site-bond percolation universality in networks
Source: Nat Commun. 2015 Dec 15;6:10196. doi: 10.1038/ncomms10196 (PMC4682156; doi:10.1038/ncomms10196)
Supplement: Supplementary Information — Supplementary Figures 1-122, Supplementary Tables 1-3, Supplementary Note 1 and Supplementary References. [file ncomms10196-s1.pdf]

Supplementary Figures

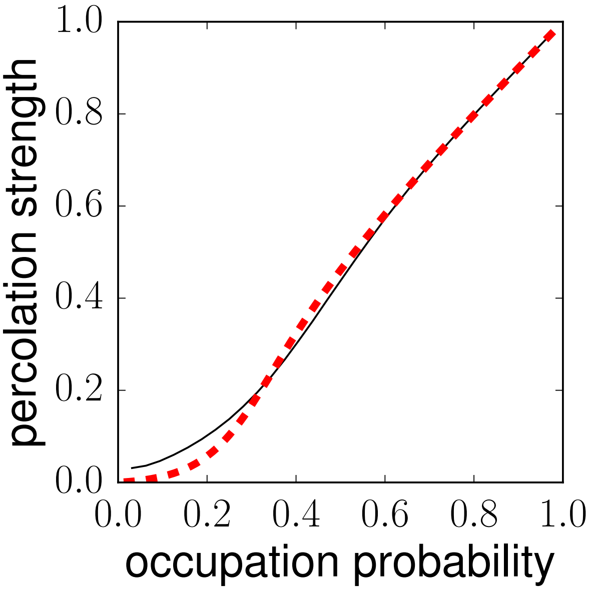

Supplementary Figure 1: Percolation diagram for the network `Social 3` [1]. The black thin line represents the site percolation order parameter S as a function of the site occupation probability p. We calculate also the order parameter B for bond percolation and multiply it by p to obtain the red dashed line.

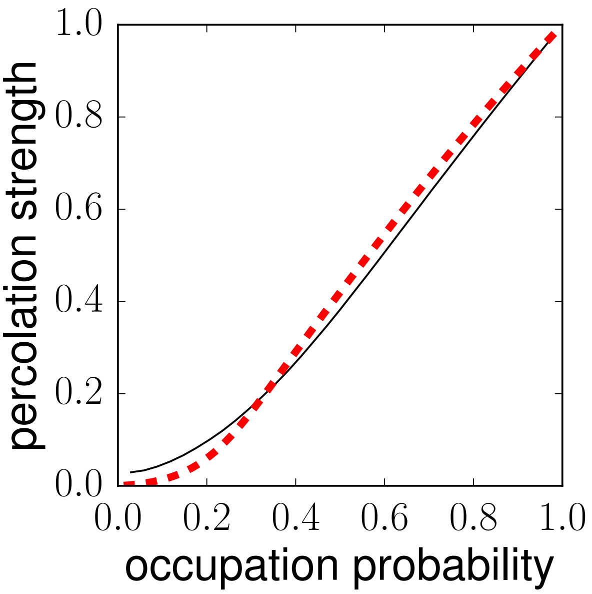

Supplementary Figure 2: Percolation diagram for the network `Karate club` [2]. The black thin line represents the site percolation order parameter S as a function of the site occupation probability p. We calculate also the order parameter B for bond percolation and multiply it by p to obtain the red dashed line.

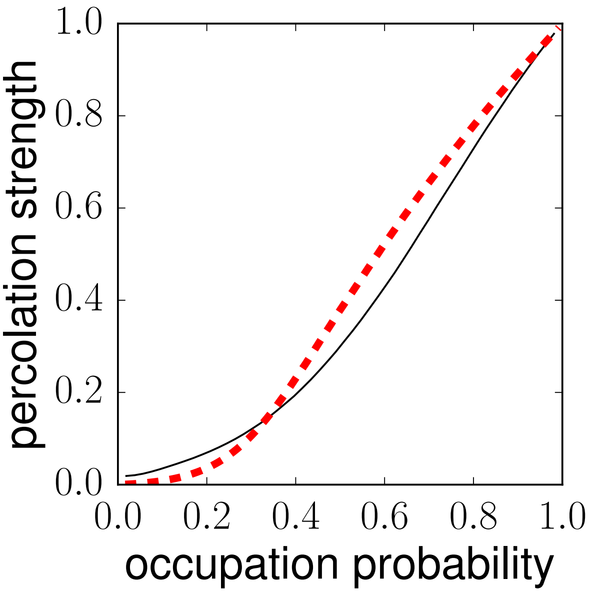

Supplementary Figure 3: Percolation diagram for the network `Protein 2` [1]. The black thin line represents the site percolation order parameter S as a function of the site occupation probability p. We calculate also the order parameter B for bond percolation and multiply it by p to obtain the red dashed line.

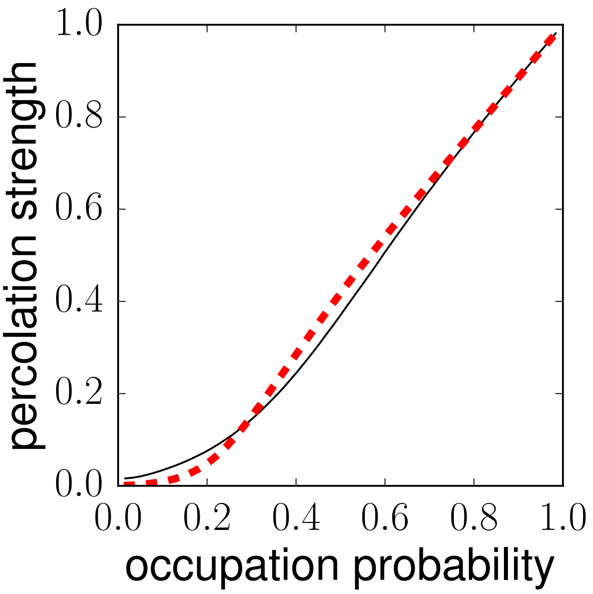

Supplementary Figure 4: Percolation diagram for the network `Dolphins` [3]. The black thin line represents the site percolation order parameter S as a function of the site occupation probability p. We calculate also the order parameter B for bond percolation and multiply it by p to obtain the red dashed line.

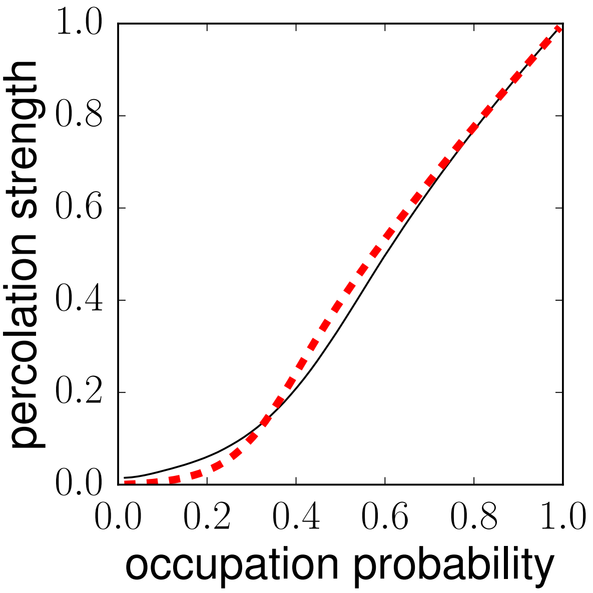

Supplementary Figure 5: Percolation diagram for the network `Social 1` [1]. The black thin line represents the site percolation order parameter S as a function of the site occupation probability p. We calculate also the order parameter B for bond percolation and multiply it by p to obtain the red dashed line.

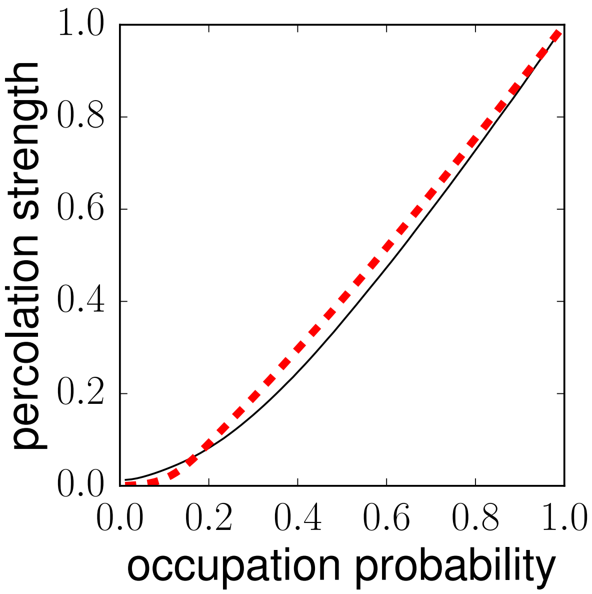

Supplementary Figure 6: Percolation diagram for the network `Les Miserables` [4]. The black thin line represents the site percolation order parameter S as a function of the site occupation probability p. We calculate also the order parameter B for bond percolation and multiply it by p to obtain the red dashed line.

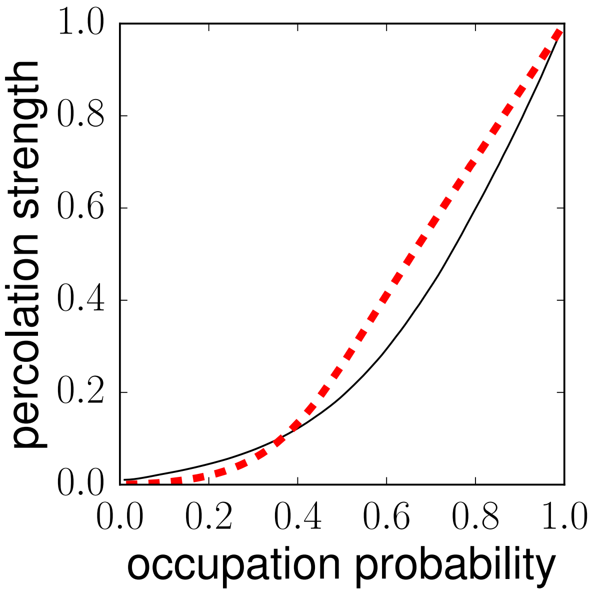

Supplementary Figure 7: Percolation diagram for the network `Protein 1` [1]. The black thin line represents the site percolation order parameter S as a function of the site occupation probability p. We calculate also the order parameter B for bond percolation and multiply it by p to obtain the red dashed line.

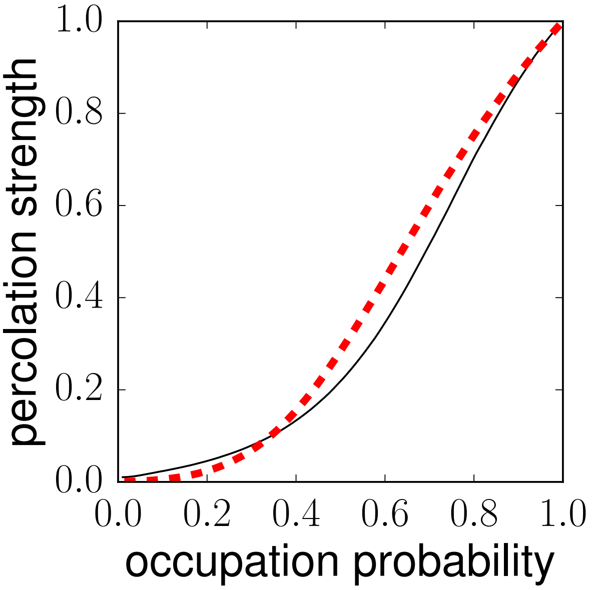

Supplementary Figure 8: Percolation diagram for the network `E. Coli, transcription` [5]. The black thin line represents the site percolation order parameter S as a function of the site occupation probability p. We calculate also the order parameter B for bond percolation and multiply it by p to obtain the red dashed line.

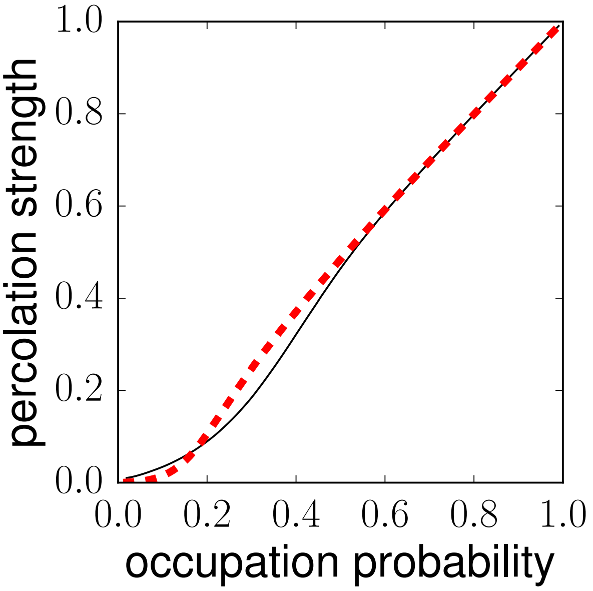

Supplementary Figure 9: Percolation diagram for the network `Political books` [6]. The black thin line represents the site percolation order parameter S as a function of the site occupation probability p. We calculate also the order parameter B for bond percolation and multiply it by p to obtain the red dashed line.

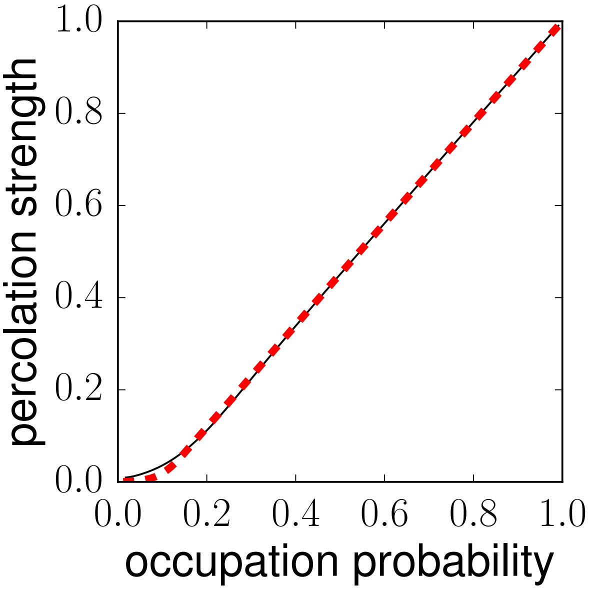

Supplementary Figure 10: Percolation diagram for the network `David Copperfield` [7]. The black thin line represents the site percolation order parameter S as a function of the site occupation probability p. We calculate also the order parameter B for bond percolation and multiply it by p to obtain the red dashed line.

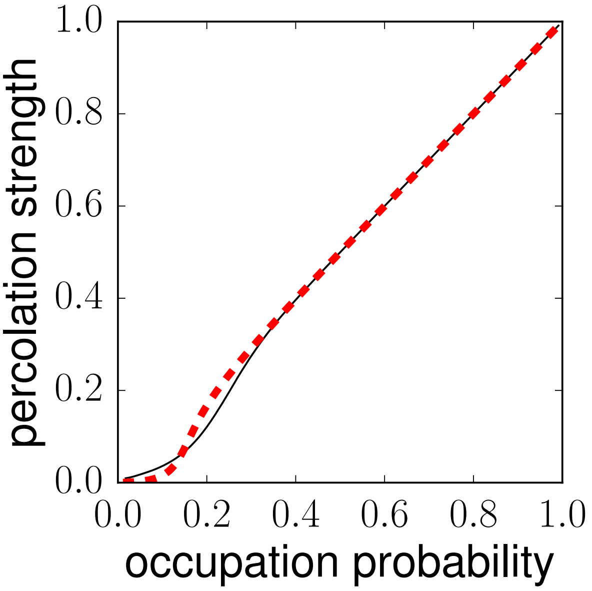

Supplementary Figure 11: Percolation diagram for the network `College football` [8]. The black thin line represents the site percolation order parameter S as a function of the site occupation probability p. We calculate also the order parameter B for bond percolation and multiply it by p to obtain the red dashed line.

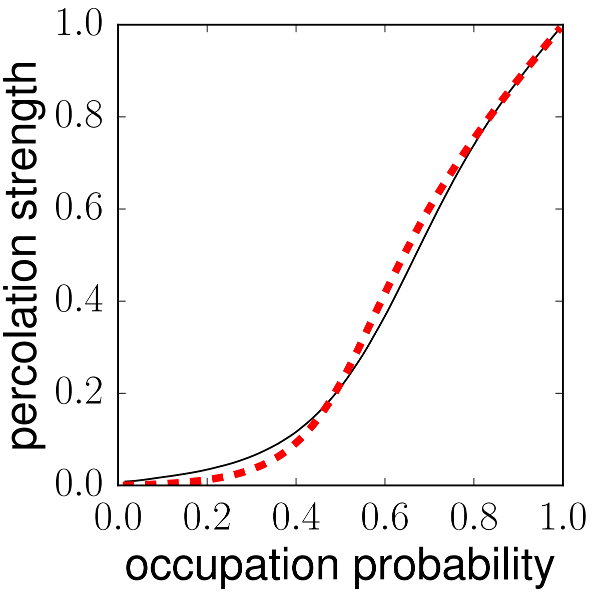

Supplementary Figure 12: Percolation diagram for the network `S 208` [1]. The black thin line represents the site percolation order parameter S as a function of the site occupation probability p. We calculate also the order parameter B for bond percolation and multiply it by p to obtain the red dashed line.

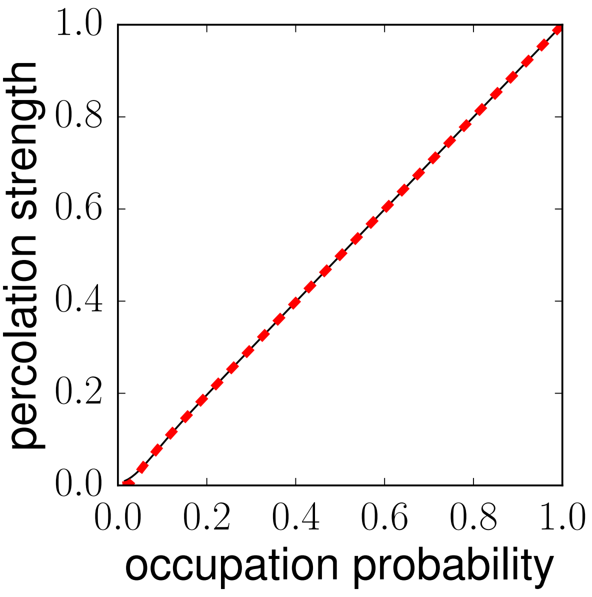

Supplementary Figure 14: Percolation diagram for the network `Bay Dry` [10, 11]. The black thin line represents the site percolation order parameter S as a function of the site occupation probability p. We calculate also the order parameter B for bond percolation and multiply it by p to obtain the red dashed line.

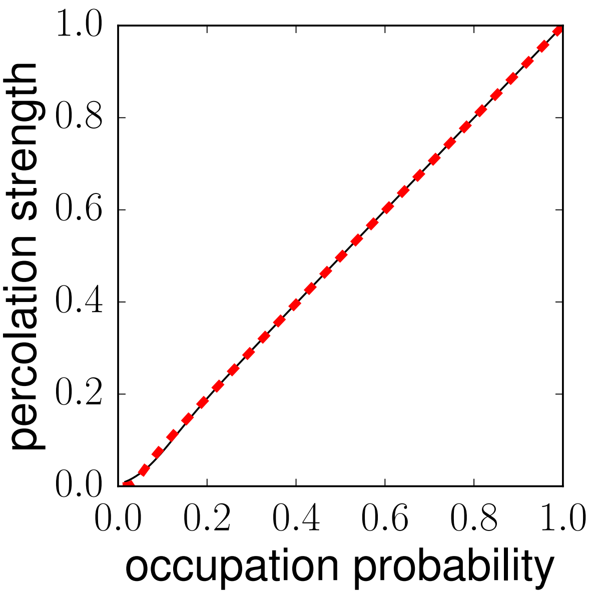

Supplementary Figure 13: Percolation diagram for the network `High school, 2011` [9]. The black thin line represents the site percolation order parameter S as a function of the site occupation probability p. We calculate also the order parameter B for bond percolation and multiply it by p to obtain the red dashed line.

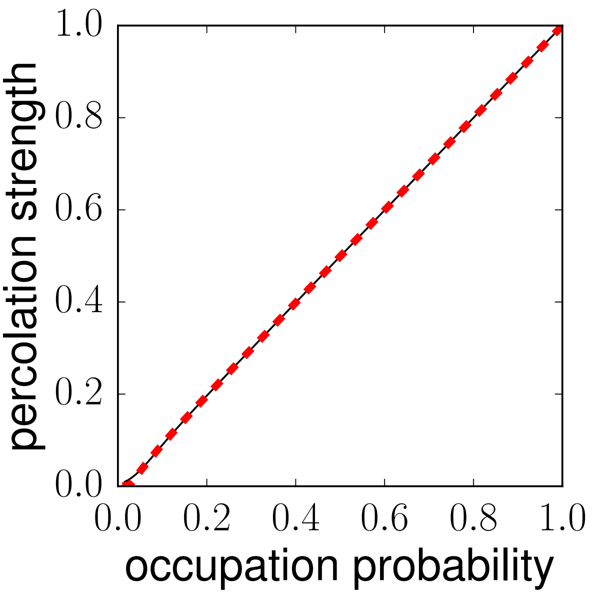

Supplementary Figure 15: Percolation diagram for the network `Bay Wet` [11]. The black thin line represents the site percolation order parameter S as a function of the site occupation probability p. We calculate also the order parameter B for bond percolation and multiply it by p to obtain the red dashed line.

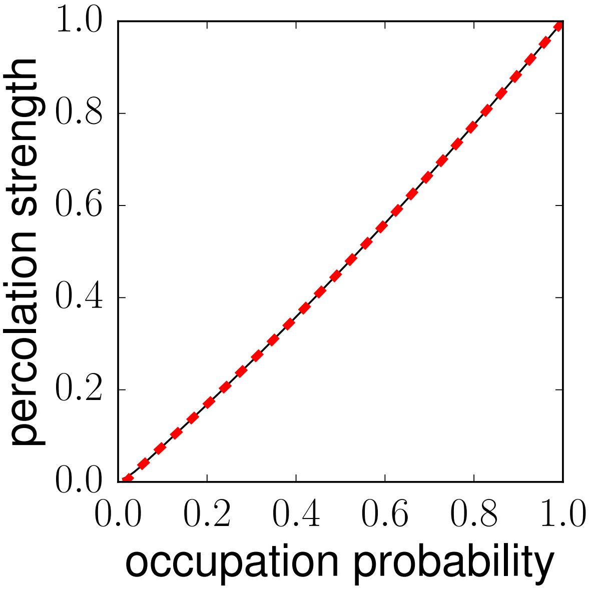

Supplementary Figure 16: Percolation diagram for the network `Radoslaw Email` [11, 12]. The black thin line represents the site percolation order parameter S as a function of the site occupation probability p. We calculate also the order parameter B for bond percolation and multiply it by p to obtain the red dashed line.

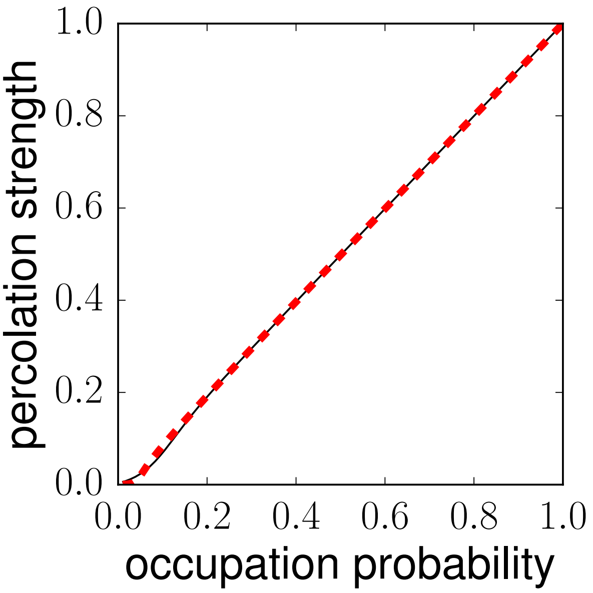

Supplementary Figure 17: Percolation diagram for the network `High school, 2012` [9]. The black thin line represents the site percolation order parameter S as a function of the site occupation probability p. We calculate also the order parameter B for bond percolation and multiply it by p to obtain the red dashed line.

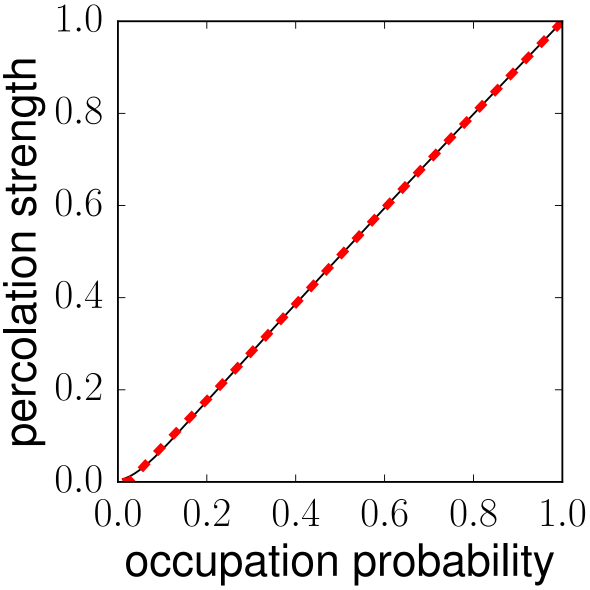

Supplementary Figure 18: Percolation diagram for the network `Little Rock Lake` [11, 13]. The black thin line represents the site percolation order parameter S as a function of the site occupation probability p. We calculate also the order parameter B for bond percolation and multiply it by p to obtain the red dashed line.

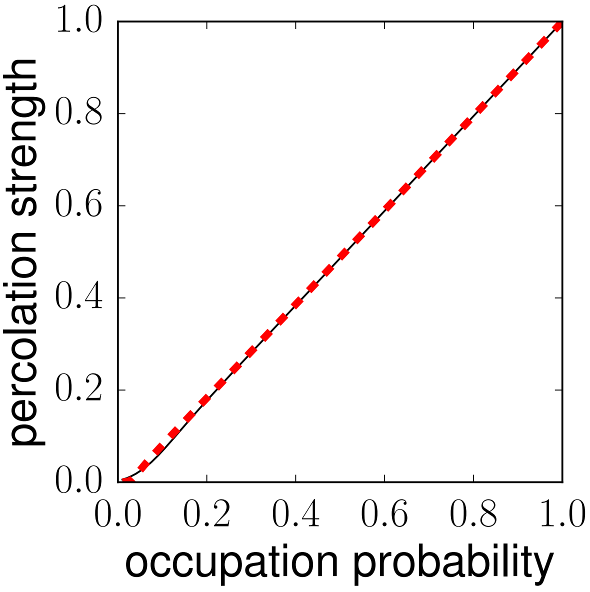

Supplementary Figure 19: Percolation diagram for the network `Jazz` [14]. The black thin line represents the site percolation order parameter S as a function of the site occupation probability p. We calculate also the order parameter B for bond percolation and multiply it by p to obtain the red dashed line.

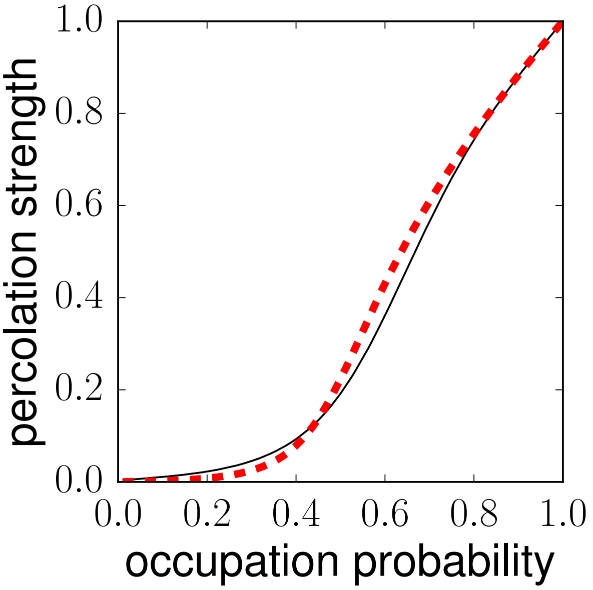

Supplementary Figure 20: Percolation diagram for the network `S 420` [1]. The black thin line represents the site percolation order parameter S as a function of the site occupation probability p. We calculate also the order parameter B for bond percolation and multiply it by p to obtain the red dashed line.

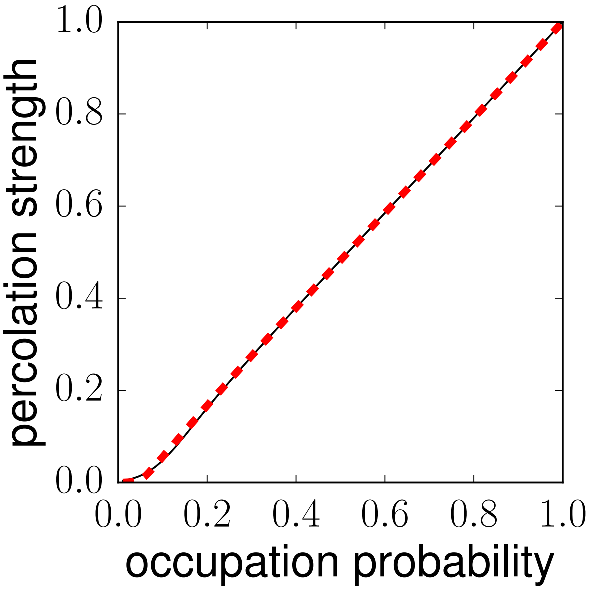

Supplementary Figure 21: Percolation diagram for the network `C. Elegans, neural` [15]. The black thin line represents the site percolation order parameter S as a function of the site occupation probability p. We calculate also the order parameter B for bond percolation and multiply it by p to obtain the red dashed line.

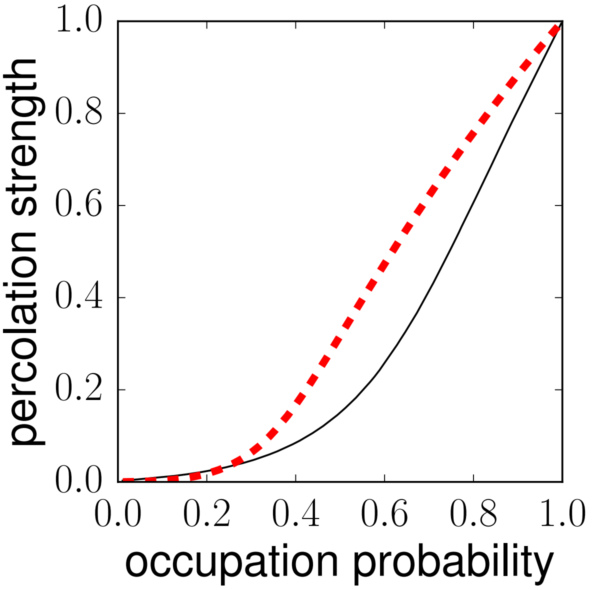

Supplementary Figure 22: Percolation diagram for the network `Network Science` [7]. The black thin line represents the site percolation order parameter S as a function of the site occupation probability p. We calculate also the order parameter B for bond percolation and multiply it by p to obtain the red dashed line.

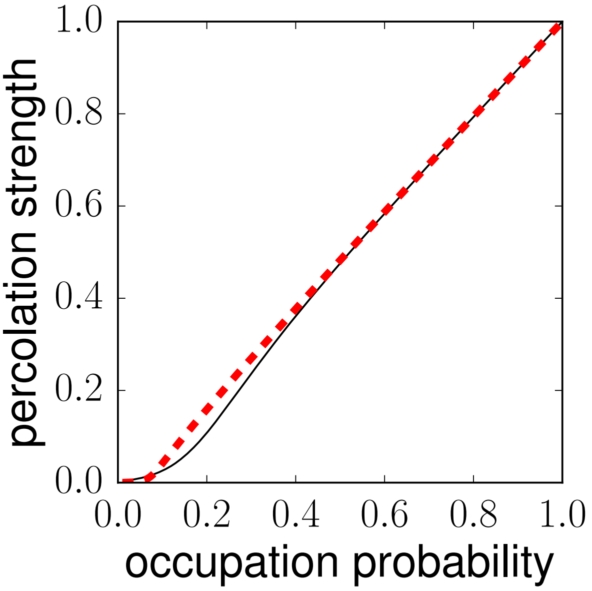

Supplementary Figure 23: Percolation diagram for the network `Dublin` [11, 16]. The black thin line represents the site percolation order parameter S as a function of the site occupation probability p. We calculate also the order parameter B for bond percolation and multiply it by p to obtain the red dashed line.

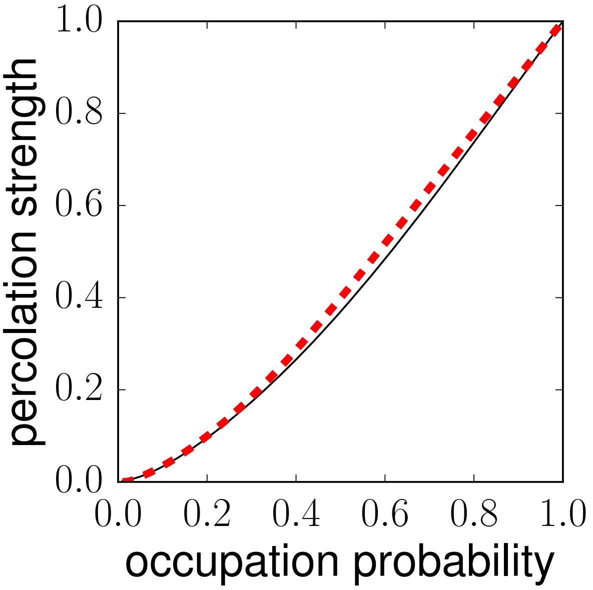

Supplementary Figure 24: Percolation diagram for the network `US Air Trasportation` [17]. The black thin line represents the site percolation order parameter S as a function of the site occupation probability p. We calculate also the order parameter B for bond percolation and multiply it by p to obtain the red dashed line.

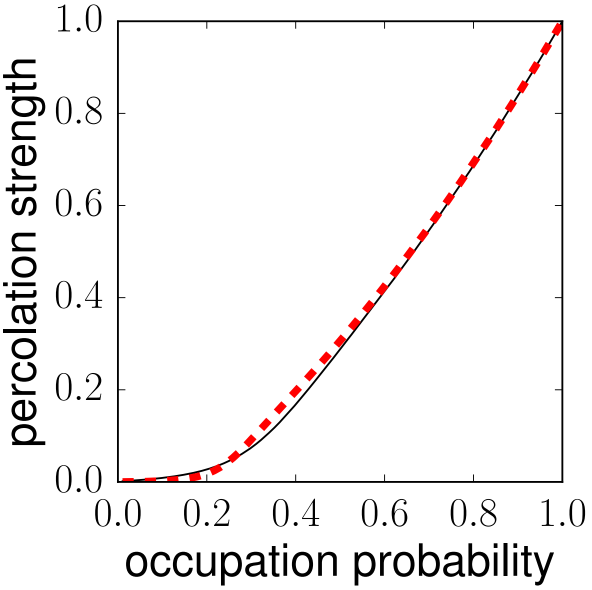

Supplementary Figure 26: Percolation diagram for the network `Yeast, transcription` [18]. The black thin line represents the site percolation order parameter S as a function of the site occupation probability p. We calculate also the order parameter B for bond percolation and multiply it by p to obtain the red dashed line.

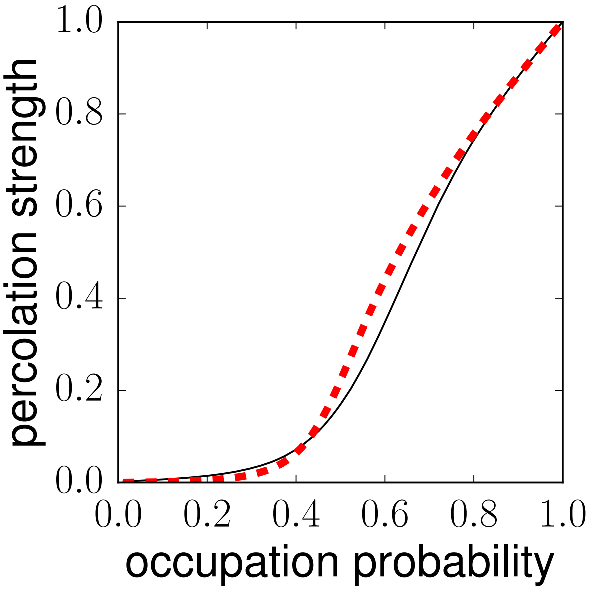

Supplementary Figure 25: Percolation diagram for the network `S 838` [1]. The black thin line represents the site percolation order parameter S as a function of the site occupation probability p. We calculate also the order parameter B for bond percolation and multiply it by p to obtain the red dashed line.

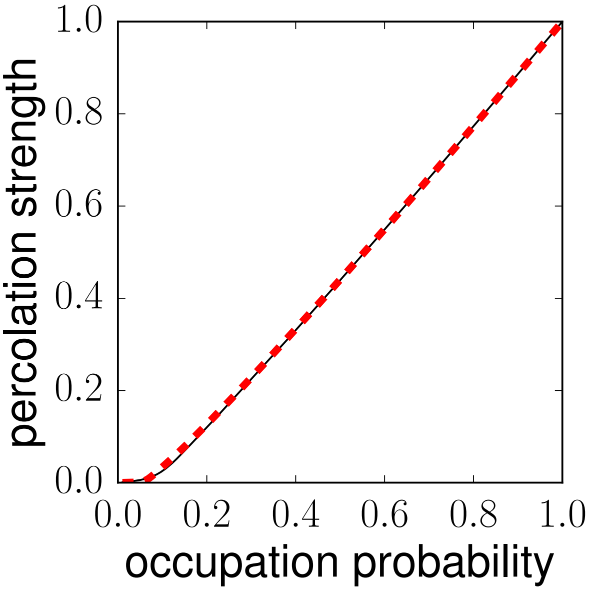

Supplementary Figure 27: Percolation diagram for the network `URV email` [19]. The black thin line represents the site percolation order parameter S as a function of the site occupation probability p. We calculate also the order parameter B for bond percolation and multiply it by p to obtain the red dashed line.

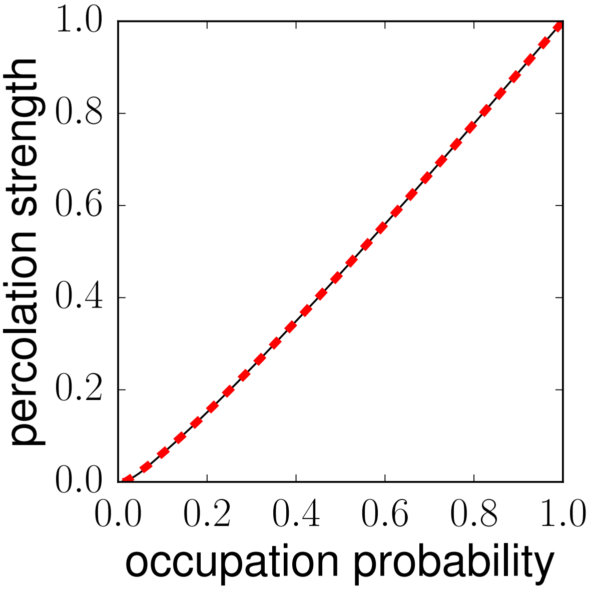

Supplementary Figure 28: Percolation diagram for the network `Political blogs` [6]. The black thin line represents the site percolation order parameter S as a function of the site occupation probability p. We calculate also the order parameter B for bond percolation and multiply it by p to obtain the red dashed line.

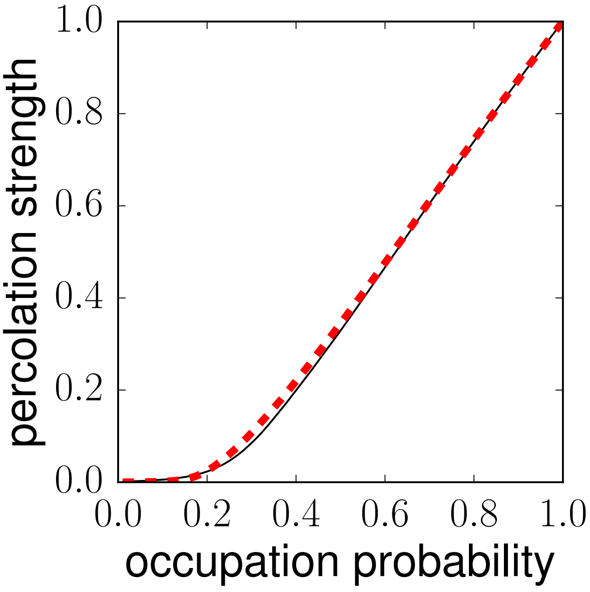

Supplementary Figure 29: Percolation diagram for the network `Air traffic` [11]. The black thin line represents the site percolation order parameter S as a function of the site occupation probability p. We calculate also the order parameter B for bond percolation and multiply it by p to obtain the red dashed line.

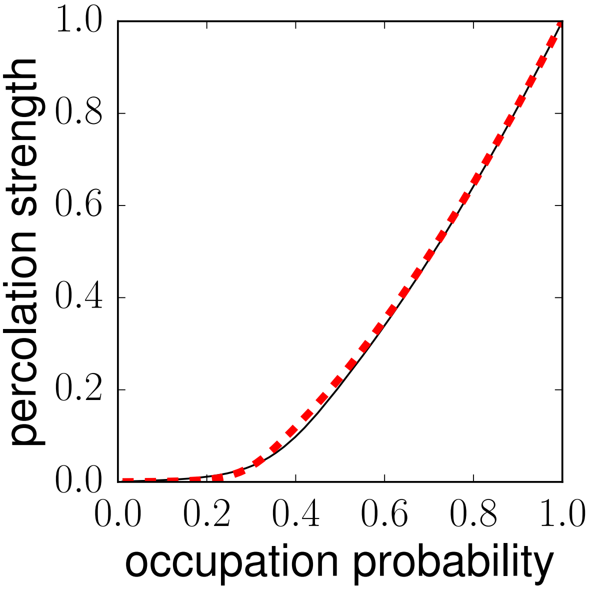

Supplementary Figure 30: Percolation diagram for the network `Yeast, protein` [20]. The black thin line represents the site percolation order parameter S as a function of the site occupation probability p. We calculate also the order parameter B for bond percolation and multiply it by p to obtain the red dashed line.

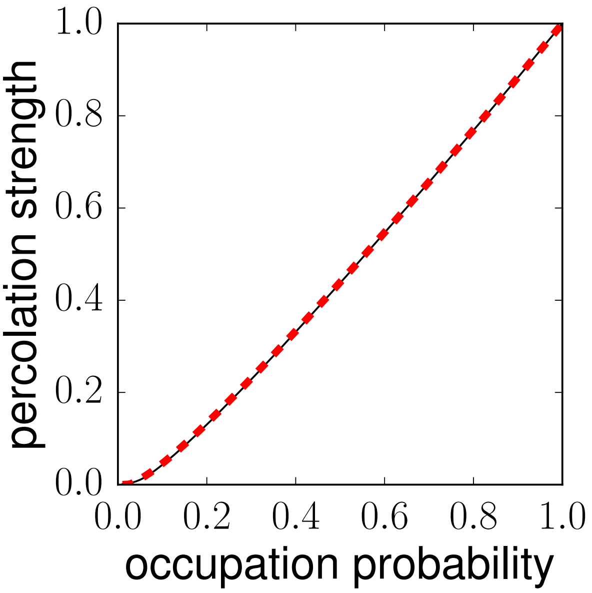

Supplementary Figure 31: Percolation diagram for the network `Petster, hamster` [11]. The black thin line represents the site percolation order parameter S as a function of the site occupation probability p. We calculate also the order parameter B for bond percolation and multiply it by p to obtain the red dashed line.

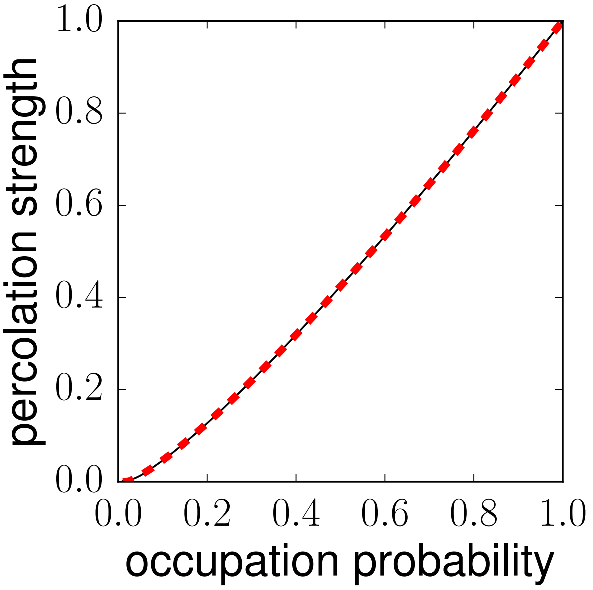

Supplementary Figure 32: Percolation diagram for the network `UC Irvine` [11, 21]. The black thin line represents the site percolation order parameter S as a function of the site occupation probability p. We calculate also the order parameter B for bond percolation and multiply it by p to obtain the red dashed line.

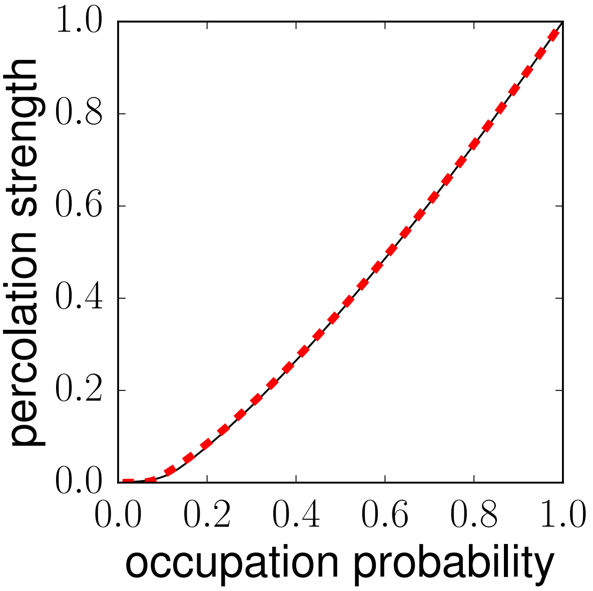

Supplementary Figure 33: Percolation diagram for the network `Yeast, protein` [22]. The black thin line represents the site percolation order parameter S as a function of the site occupation probability p. We calculate also the order parameter B for bond percolation and multiply it by p to obtain the red dashed line.

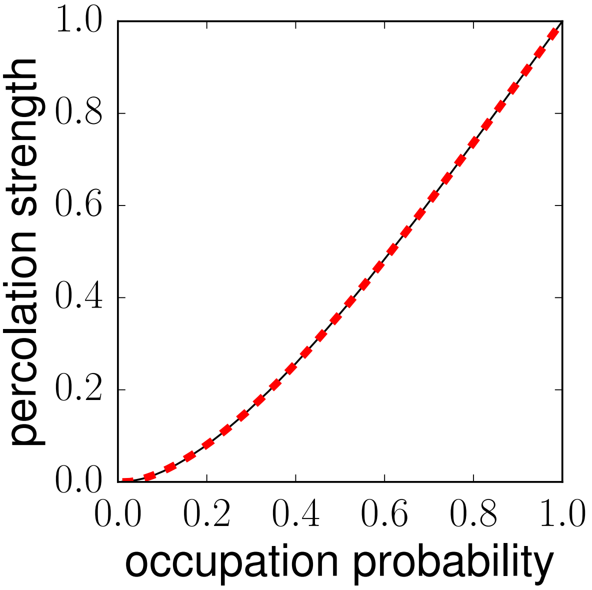

Supplementary Figure 34: Percolation diagram for the network `Japanese` [1]. The black thin line represents the site percolation order parameter S as a function of the site occupation probability p. We calculate also the order parameter B for bond percolation and multiply it by p to obtain the red dashed line.

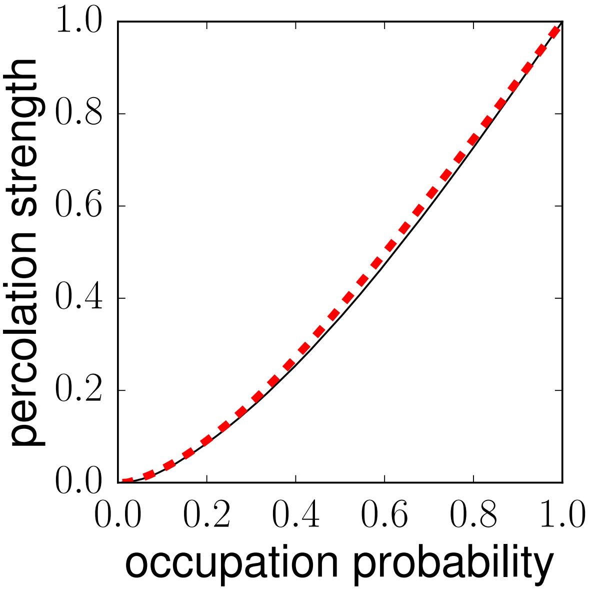

Supplementary Figure 35: Percolation diagram for the network `Open flights` [11, 23]. The black thin line represents the site percolation order parameter S as a function of the site occupation probability p. We calculate also the order parameter B for bond percolation and multiply it by p to obtain the red dashed line.

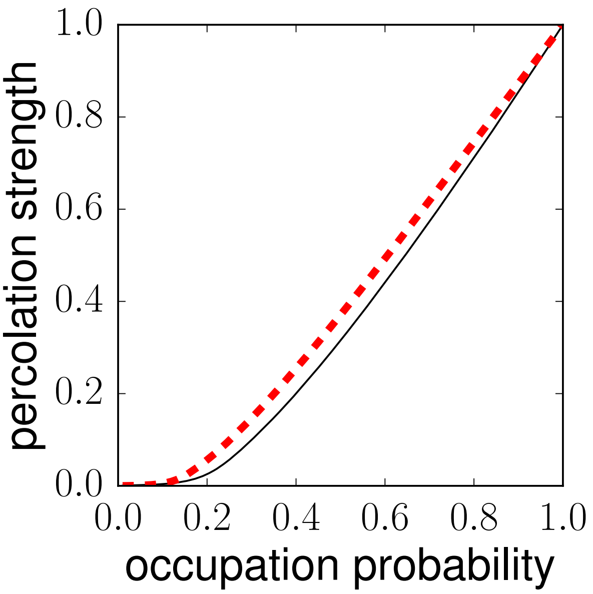

Supplementary Figure 36: Percolation diagram for the network `GR-QC, 1993-2003` [24]. The black thin line represents the site percolation order parameter S as a function of the site occupation probability p. We calculate also the order parameter B for bond percolation and multiply it by p to obtain the red dashed line.

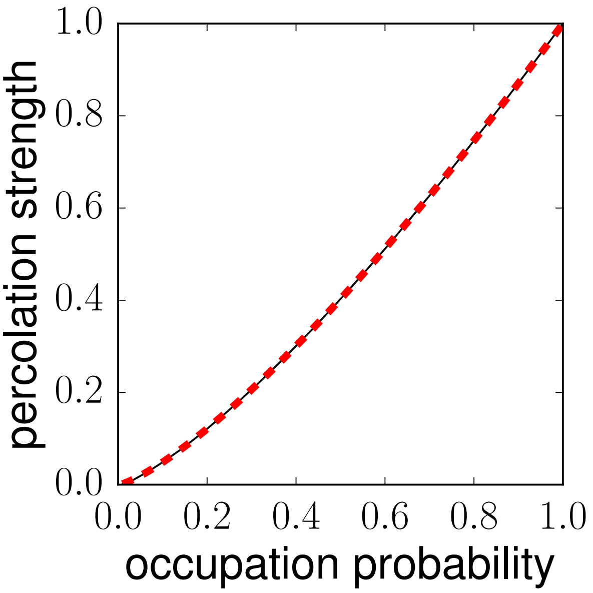

Supplementary Figure 37: Percolation diagram for the network `Tennis` [25]. The black thin line represents the site percolation order parameter S as a function of the site occupation probability p. We calculate also the order parameter B for bond percolation and multiply it by p to obtain the red dashed line.

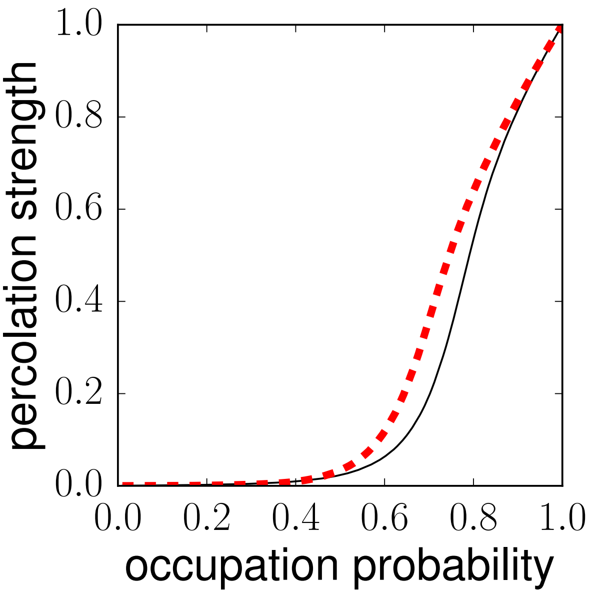

Supplementary Figure 38: Percolation diagram for the network `US Power grid` [15]. The black thin line represents the site percolation order parameter S as a function of the site occupation probability p. We calculate also the order parameter B for bond percolation and multiply it by p to obtain the red dashed line.

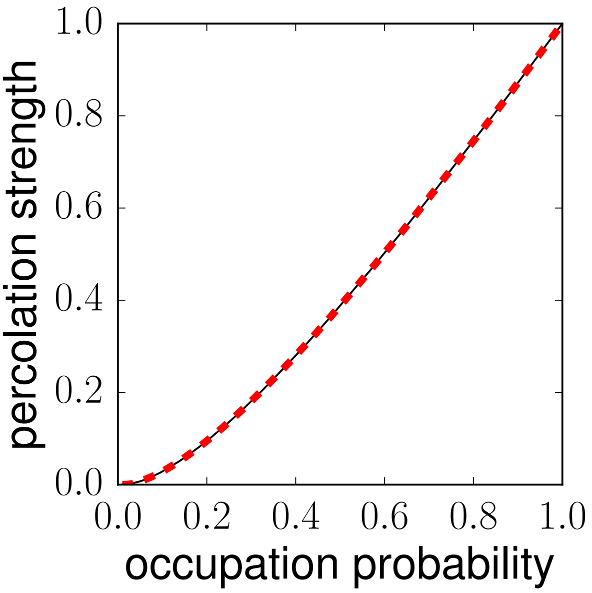

Supplementary Figure 39: Percolation diagram for the network `HT09` [16]. The black thin line represents the site percolation order parameter S as a function of the site occupation probability p. We calculate also the order parameter B for bond percolation and multiply it by p to obtain the red dashed line.

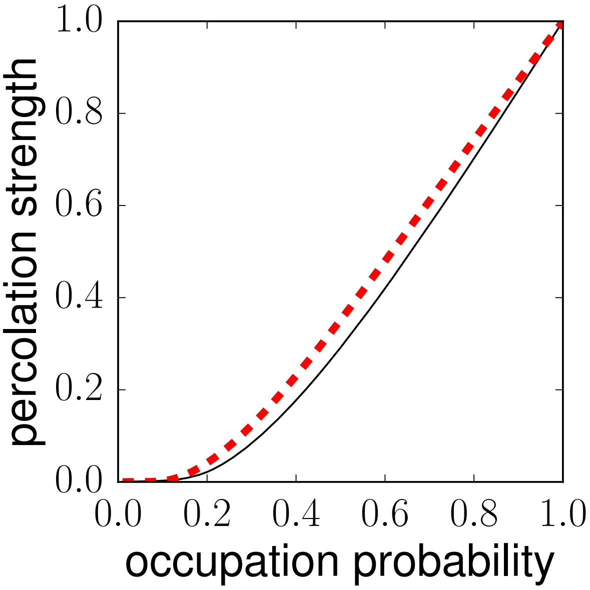

Supplementary Figure 40: Percolation diagram for the network `Hep-Th, 1995-1999` [26]. The black thin line represents the site percolation order parameter S as a function of the site occupation probability p. We calculate also the order parameter B for bond percolation and multiply it by p to obtain the red dashed line.

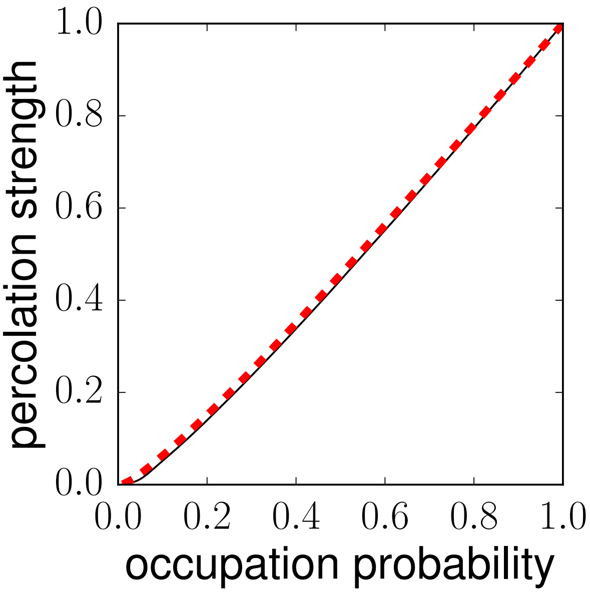

Supplementary Figure 41: Percolation diagram for the network `Reactome` [11, 27]. The black thin line represents the site percolation order parameter S as a function of the site occupation probability p. We calculate also the order parameter B for bond percolation and multiply it by p to obtain the red dashed line.

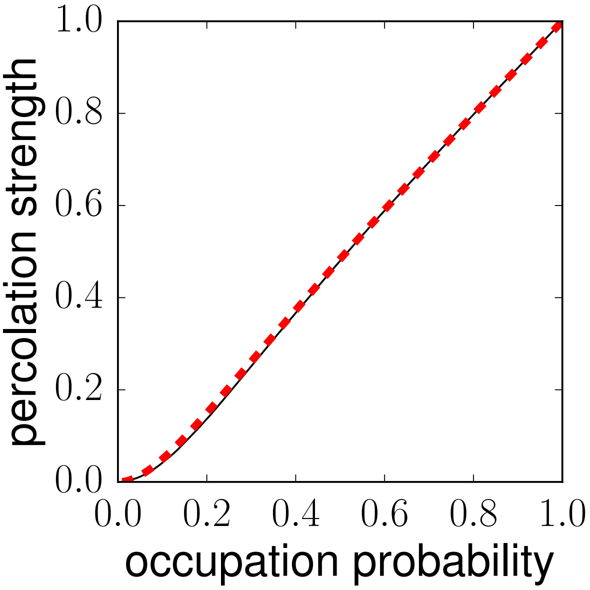

Supplementary Figure 42: Percolation diagram for the network `Jung` [11, 28]. The black thin line represents the site percolation order parameter S as a function of the site occupation probability p. We calculate also the order parameter B for bond percolation and multiply it by p to obtain the red dashed line.

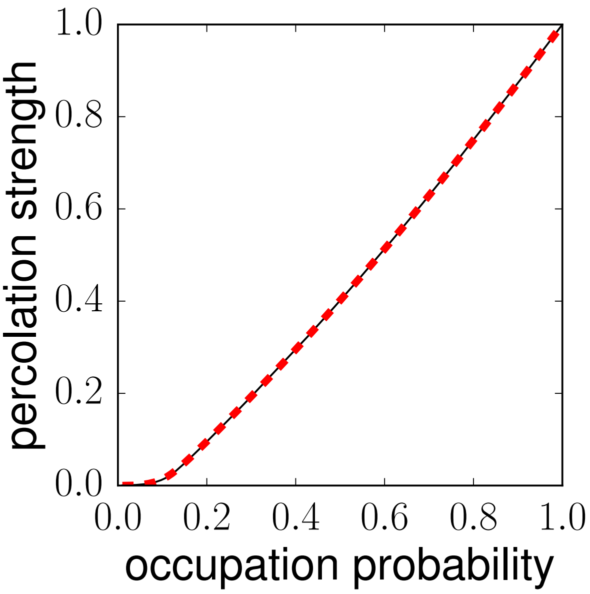

Supplementary Figure 43: Percolation diagram for the network `Gnutella, Aug. 8, 2002` [24, 29]. The black thin line represents the site percolation order parameter S as a function of the site occupation probability p. We calculate also the order parameter B for bond percolation and multiply it by p to obtain the red dashed line.

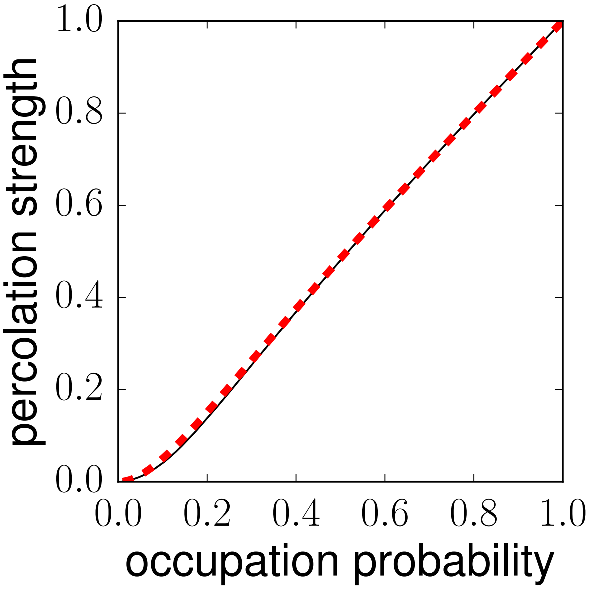

Supplementary Figure 44: Percolation diagram for the network `JDK` [11]. The black thin line represents the site percolation order parameter S as a function of the site occupation probability p. We calculate also the order parameter B for bond percolation and multiply it by p to obtain the red dashed line.

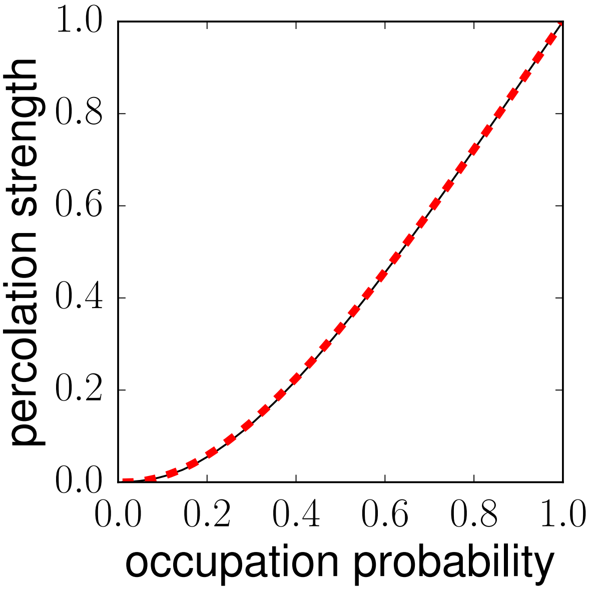

Supplementary Figure 45: Percolation diagram for the network `AS Oregon` [30]. The black thin line represents the site percolation order parameter S as a function of the site occupation probability p. We calculate also the order parameter B for bond percolation and multiply it by p to obtain the red dashed line.

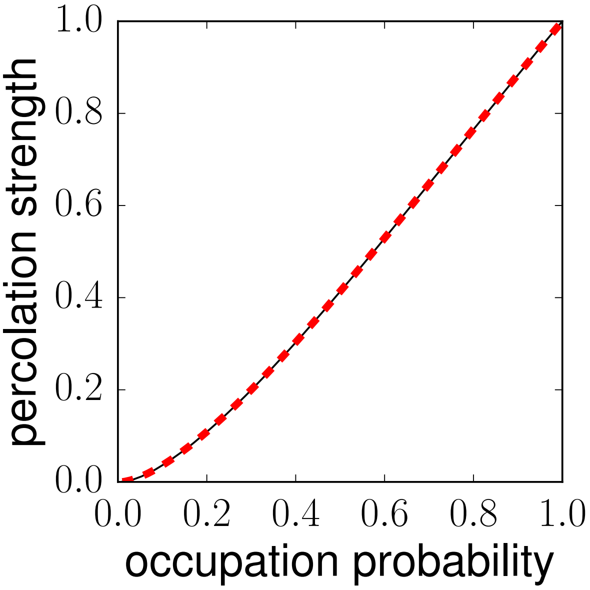

Supplementary Figure 46: Percolation diagram for the network `English` [1]. The black thin line represents the site percolation order parameter S as a function of the site occupation probability p. We calculate also the order parameter B for bond percolation and multiply it by p to obtain the red dashed line.

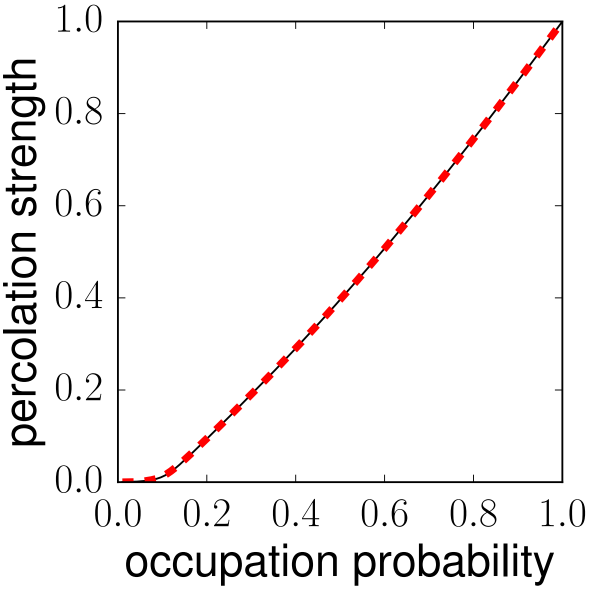

Supplementary Figure 47: Percolation diagram for the network `Gnutella, Aug. 9, 2002` [24, 29]. The black thin line represents the site percolation order parameter S as a function of the site occupation probability p. We calculate also the order parameter B for bond percolation and multiply it by p to obtain the red dashed line.

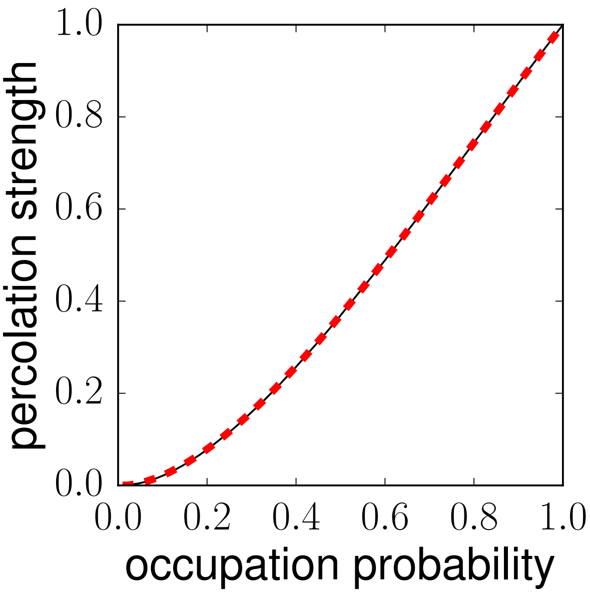

Supplementary Figure 48: Percolation diagram for the network `French` [1]. The black thin line represents the site percolation order parameter S as a function of the site occupation probability p. We calculate also the order parameter B for bond percolation and multiply it by p to obtain the red dashed line.

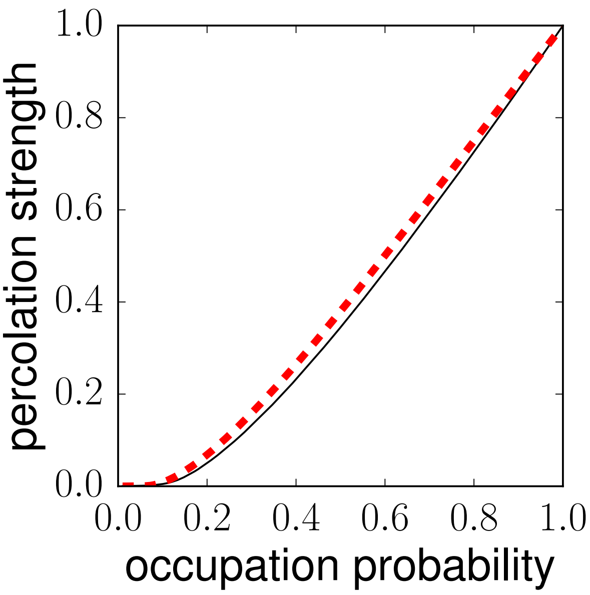

Supplementary Figure 49: Percolation diagram for the network `Hep-Th, 1993-2003` [24]. The black thin line represents the site percolation order parameter S as a function of the site occupation probability p. We calculate also the order parameter B for bond percolation and multiply it by p to obtain the red dashed line.

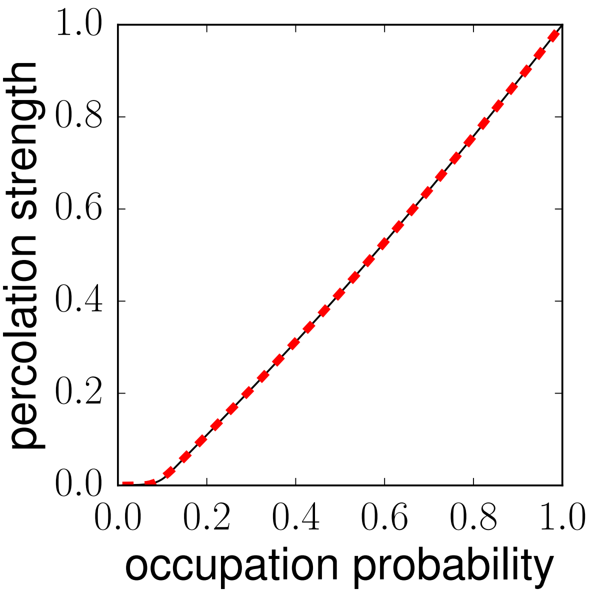

Supplementary Figure 50: Percolation diagram for the network `Gnutella, Aug. 6, 2002` [24, 29]. The black thin line represents the site percolation order parameter S as a function of the site occupation probability p. We calculate also the order parameter B for bond percolation and multiply it by p to obtain the red dashed line.

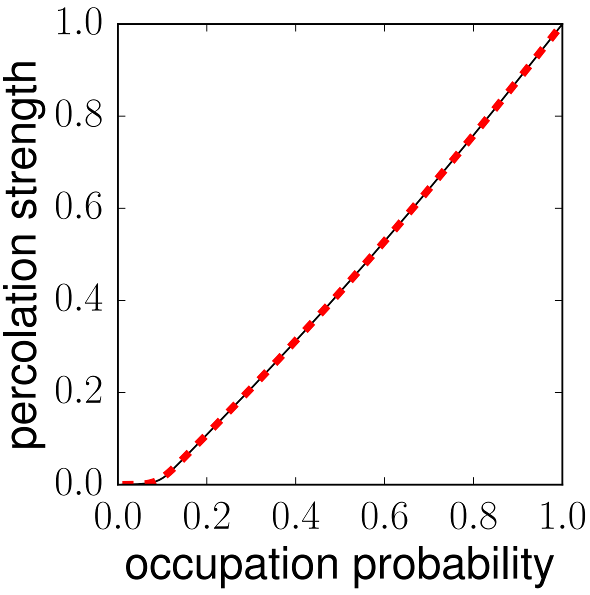

Supplementary Figure 51: Percolation diagram for the network `Gnutella, Aug. 5, 2002` [24, 29]. The black thin line represents the site percolation order parameter S as a function of the site occupation probability p. We calculate also the order parameter B for bond percolation and multiply it by p to obtain the red dashed line.

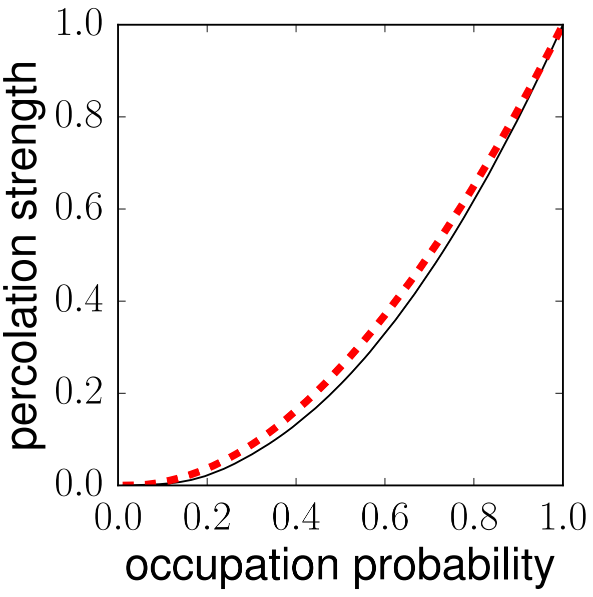

Supplementary Figure 52: Percolation diagram for the network `PGP` [31]. The black thin line represents the site percolation order parameter S as a function of the site occupation probability p. We calculate also the order parameter B for bond percolation and multiply it by p to obtain the red dashed line.

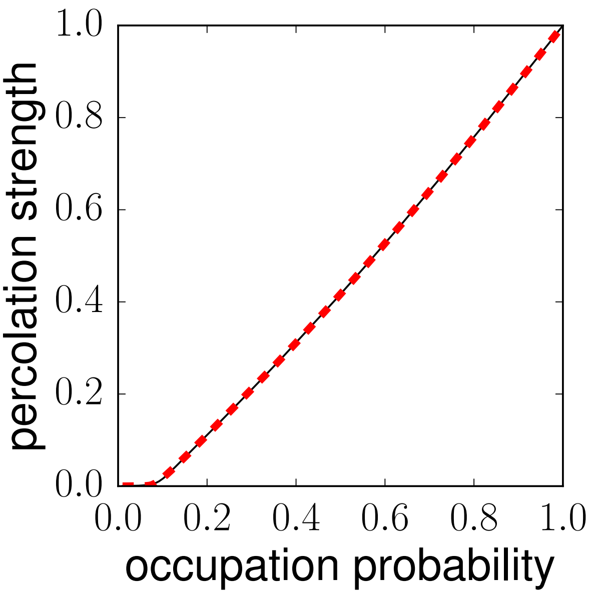

Supplementary Figure 53: Percolation diagram for the network `Gnutella, August 4 2002` [24, 29]. The black thin line represents the site percolation order parameter S as a function of the site occupation probability p. We calculate also the order parameter B for bond percolation and multiply it by p to obtain the red dashed line.

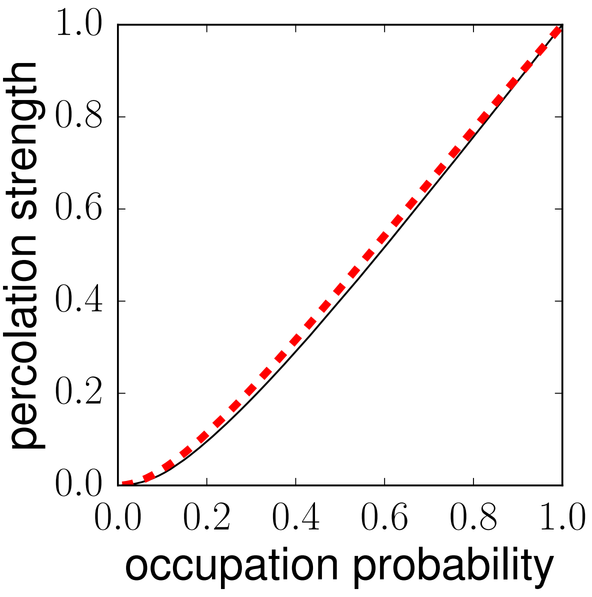

Supplementary Figure 54: Percolation diagram for the network `Hep-Ph, 1993-2003` [24]. The black thin line represents the site percolation order parameter S as a function of the site occupation probability p. We calculate also the order parameter B for bond percolation and multiply it by p to obtain the red dashed line.

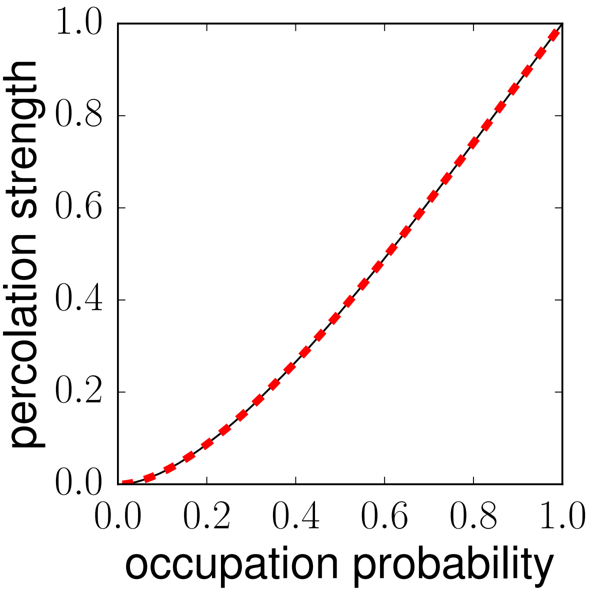

Supplementary Figure 55: Percolation diagram for the network `Spanish` [1]. The black thin line represents the site percolation order parameter S as a function of the site occupation probability p. We calculate also the order parameter B for bond percolation and multiply it by p to obtain the red dashed line.

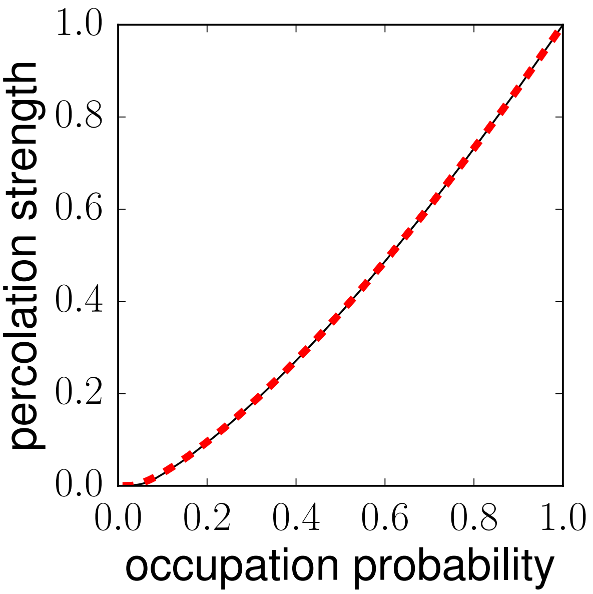

Supplementary Figure 56: Percolation diagram for the network `DBLP, citations` [11, 32]. The black thin line represents the site percolation order parameter S as a function of the site occupation probability p. We calculate also the order parameter B for bond percolation and multiply it by p to obtain the red dashed line.

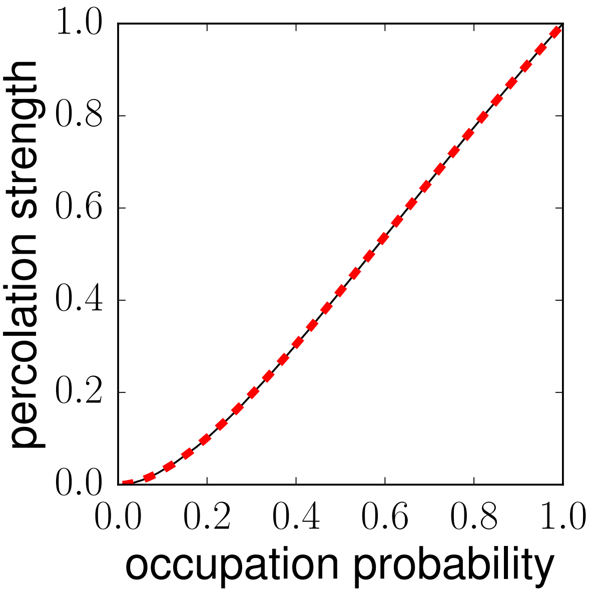

Supplementary Figure 57: Percolation diagram for the network `Spanish` [11]. The black thin line represents the site percolation order parameter S as a function of the site occupation probability p. We calculate also the order parameter B for bond percolation and multiply it by p to obtain the red dashed line.

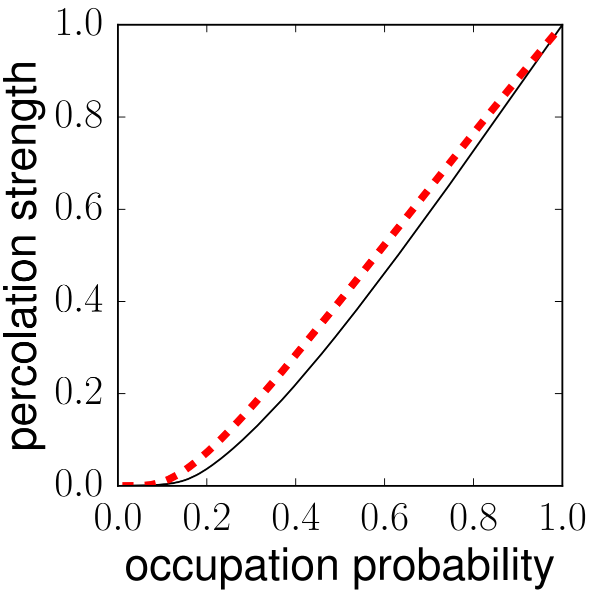

Supplementary Figure 58: Percolation diagram for the network `Cond-Mat, 1995-1999` [26]. The black thin line represents the site percolation order parameter S as a function of the site occupation probability p. We calculate also the order parameter B for bond percolation and multiply it by p to obtain the red dashed line.

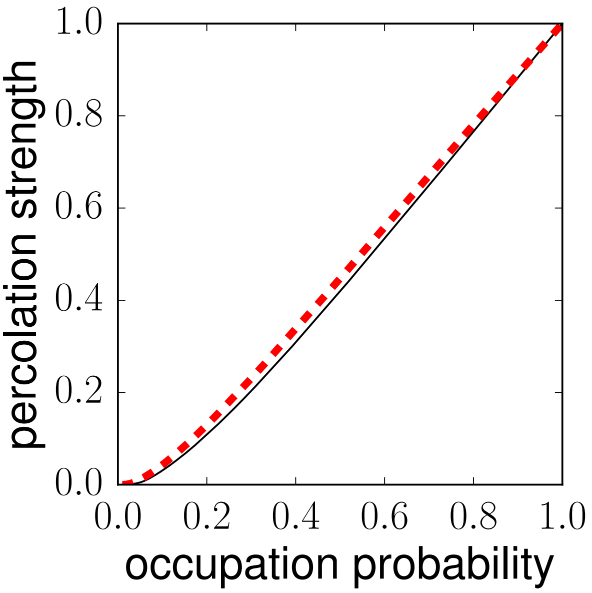

Supplementary Figure 59: Percolation diagram for the network `Astrophysics` [26]. The black thin line represents the site percolation order parameter S as a function of the site occupation probability p. We calculate also the order parameter B for bond percolation and multiply it by p to obtain the red dashed line.

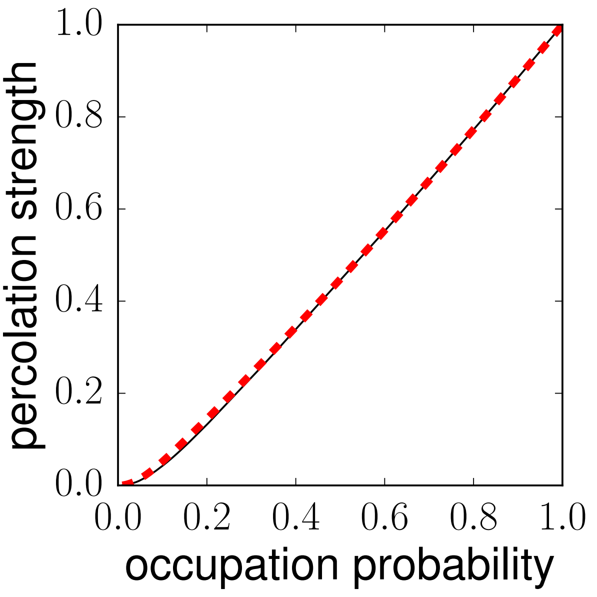

Supplementary Figure 60: Percolation diagram for the network `Google` [33]. The black thin line represents the site percolation order parameter S as a function of the site occupation probability p. We calculate also the order parameter B for bond percolation and multiply it by p to obtain the red dashed line.

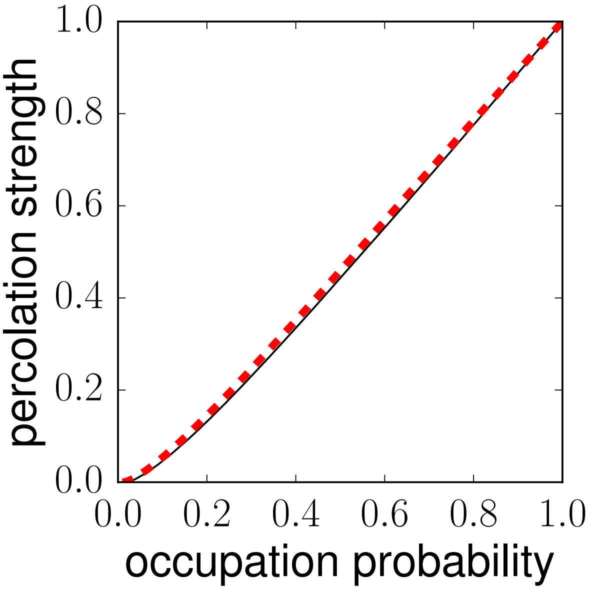

Supplementary Figure 61: Percolation diagram for the network `AstroPhys, 1993-2003` [24]. The black thin line represents the site percolation order parameter S as a function of the site occupation probability p. We calculate also the order parameter B for bond percolation and multiply it by p to obtain the red dashed line.

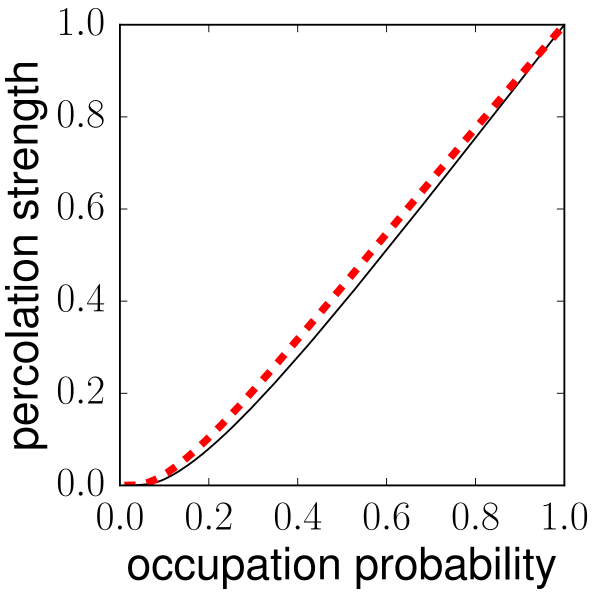

Supplementary Figure 62: Percolation diagram for the network `Cond-Mat, 1993-2003` [24]. The black thin line represents the site percolation order parameter S as a function of the site occupation probability p. We calculate also the order parameter B for bond percolation and multiply it by p to obtain the red dashed line.

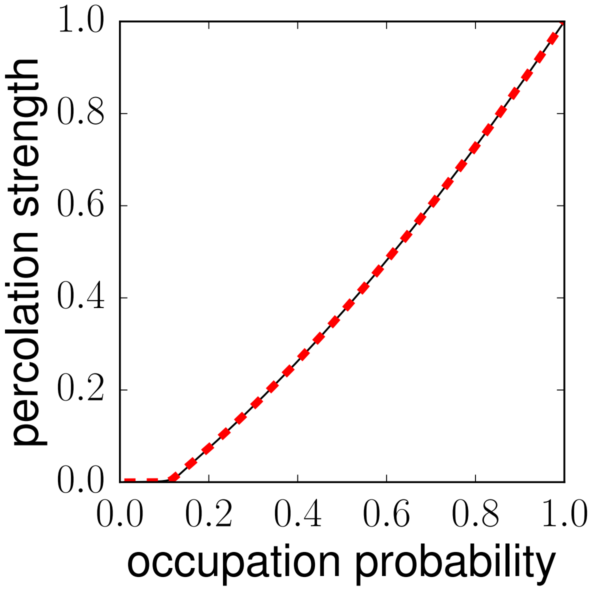

Supplementary Figure 63: Percolation diagram for the network `Gnutella, Aug. 25, 2002` [24, 29]. The black thin line represents the site percolation order parameter S as a function of the site occupation probability p. We calculate also the order parameter B for bond percolation and multiply it by p to obtain the red dashed line.

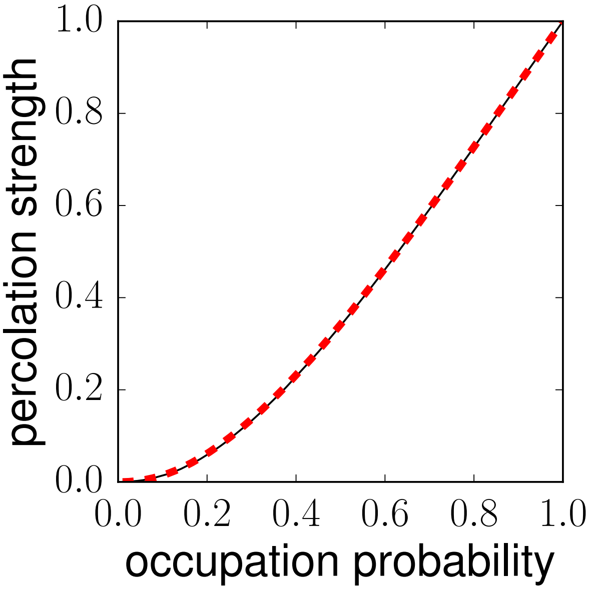

Supplementary Figure 64: Percolation diagram for the network `Internet`. The black thin line represents the site percolation order parameter S as a function of the site occupation probability p. We calculate also the order parameter B for bond percolation and multiply it by p to obtain the red dashed line.

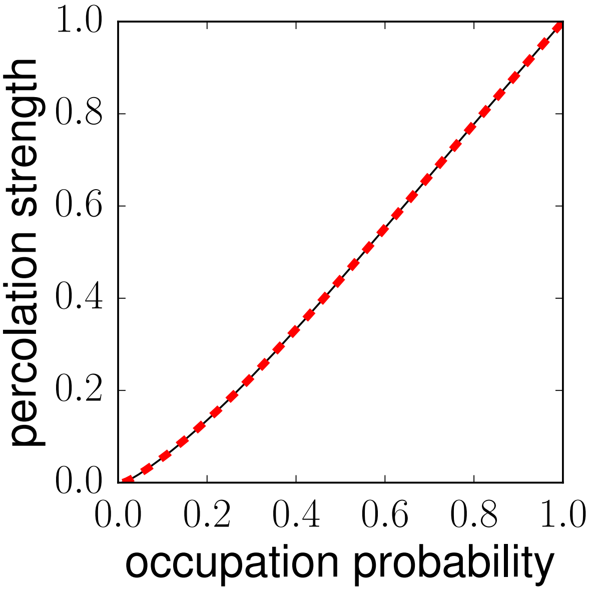

Supplementary Figure 65: Percolation diagram for the network `Thesaurus` [11, 34]. The black thin line represents the site percolation order parameter S as a function of the site occupation probability p. We calculate also the order parameter B for bond percolation and multiply it by p to obtain the red dashed line.

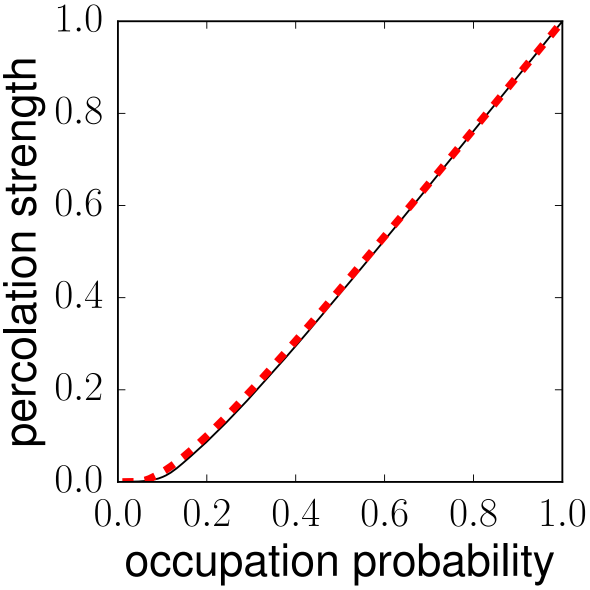

Supplementary Figure 66: Percolation diagram for the network `Cora` [11, 35]. The black thin line represents the site percolation order parameter S as a function of the site occupation probability p. We calculate also the order parameter B for bond percolation and multiply it by p to obtain the red dashed line.

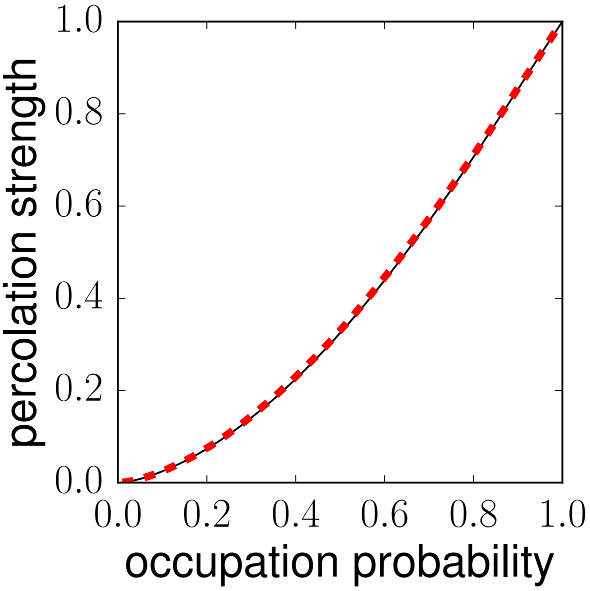

Supplementary Figure 67: Percolation diagram for the network `Linux, mailing list` [11]. The black thin line represents the site percolation order parameter S as a function of the site occupation probability p. We calculate also the order parameter B for bond percolation and multiply it by p to obtain the red dashed line.

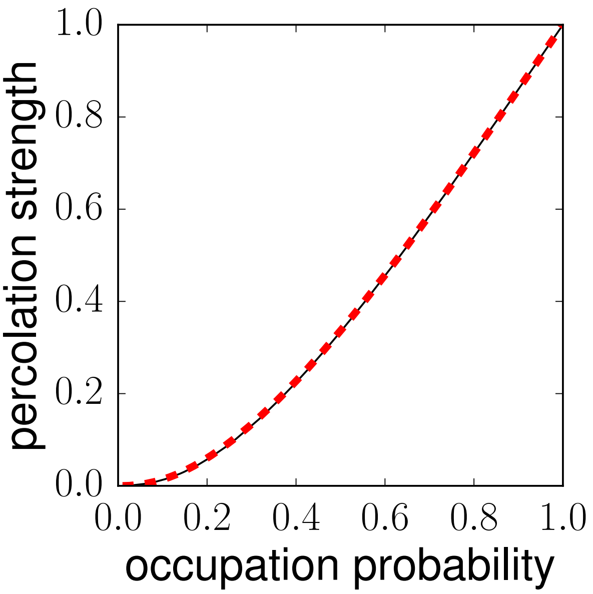

Supplementary Figure 68: Percolation diagram for the network `AS Caida` [30]. The black thin line represents the site percolation order parameter S as a function of the site occupation probability p. We calculate also the order parameter B for bond percolation and multiply it by p to obtain the red dashed line.

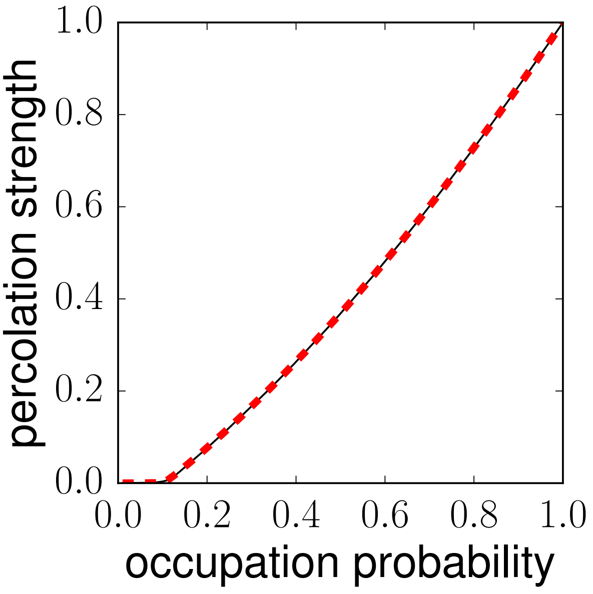

Supplementary Figure 69: Percolation diagram for the network `Gnutella, Aug. 24, 2002` [24, 29]. The black thin line represents the site percolation order parameter S as a function of the site occupation probability p. We calculate also the order parameter B for bond percolation and multiply it by p to obtain the red dashed line.

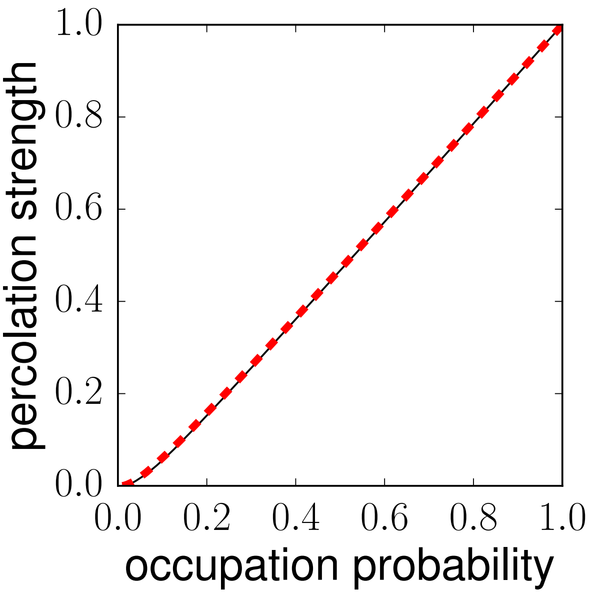

Supplementary Figure 70: Percolation diagram for the network `Hep-Th, citations` [11, 24]. The black thin line represents the site percolation order parameter S as a function of the site occupation probability p. We calculate also the order parameter B for bond percolation and multiply it by p to obtain the red dashed line.

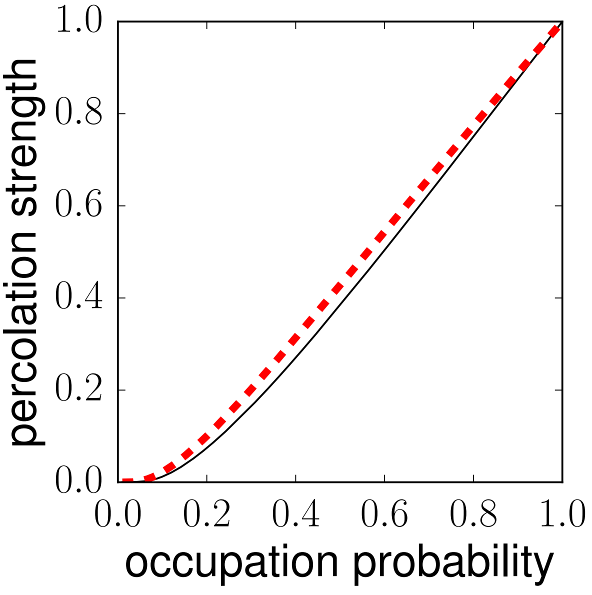

Supplementary Figure 71: Percolation diagram for the network `Cond-Mat, 1995-2003` [26]. The black thin line represents the site percolation order parameter S as a function of the site occupation probability p. We calculate also the order parameter B for bond percolation and multiply it by p to obtain the red dashed line.

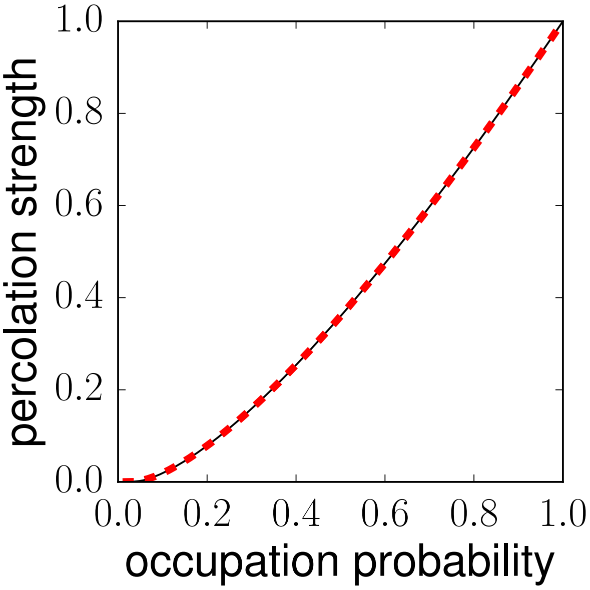

Supplementary Figure 72: Percolation diagram for the network `Digg` [11, 36]. The black thin line represents the site percolation order parameter S as a function of the site occupation probability p. We calculate also the order parameter B for bond percolation and multiply it by p to obtain the red dashed line.

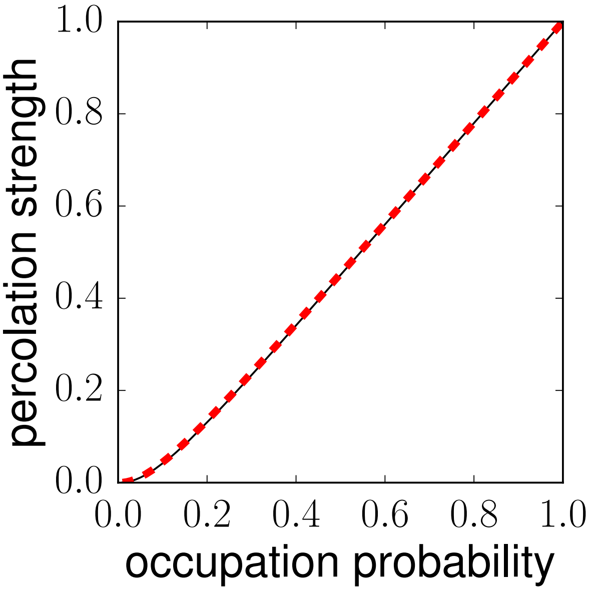

Supplementary Figure 73: Percolation diagram for the network `Linux, soft.` [11]. The black thin line represents the site percolation order parameter S as a function of the site occupation probability p. We calculate also the order parameter B for bond percolation and multiply it by p to obtain the red dashed line.

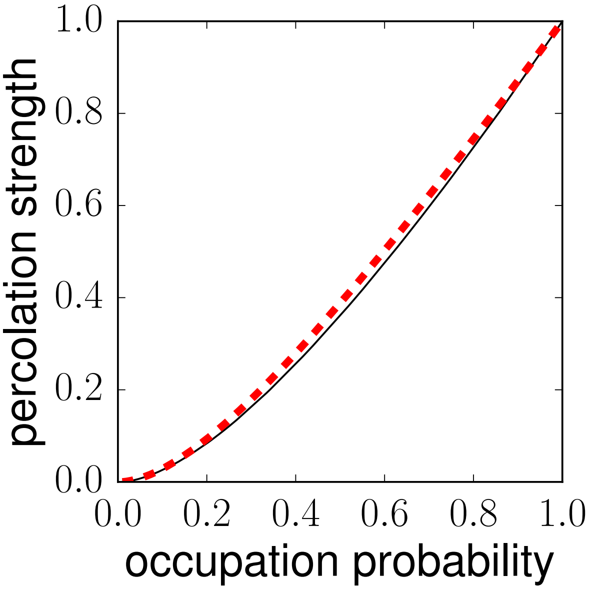

Supplementary Figure 74: Percolation diagram for the network `Enron` [37]. The black thin line represents the site percolation order parameter S as a function of the site occupation probability p. We calculate also the order parameter B for bond percolation and multiply it by p to obtain the red dashed line.

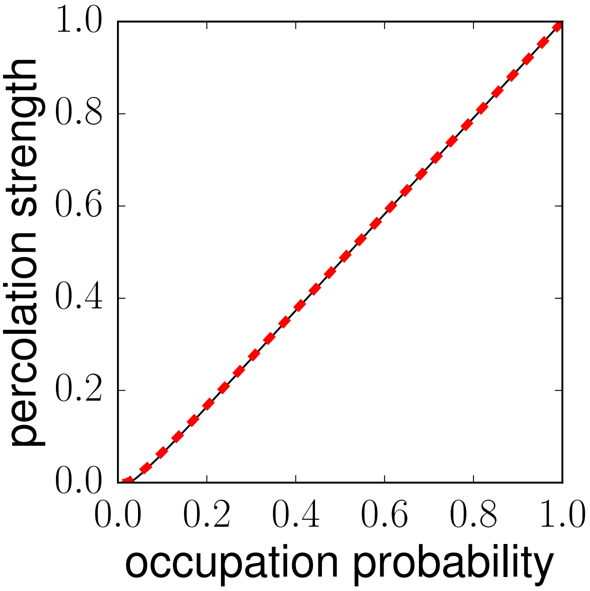

Supplementary Figure 75: Percolation diagram for the network `Hep-Ph, citations` [11, 24]. The black thin line represents the site percolation order parameter S as a function of the site occupation probability p. We calculate also the order parameter B for bond percolation and multiply it by p to obtain the red dashed line.

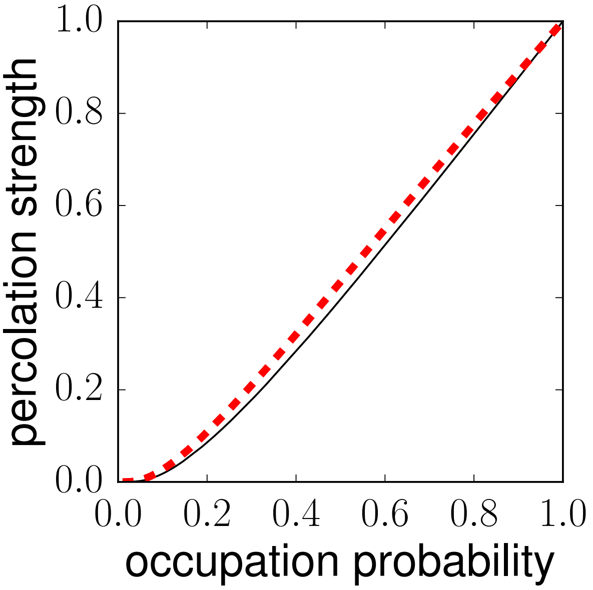

Supplementary Figure 76: Percolation diagram for the network `Cond-Mat, 1995-2005` [26]. The black thin line represents the site percolation order parameter S as a function of the site occupation probability p. We calculate also the order parameter B for bond percolation and multiply it by p to obtain the red dashed line.

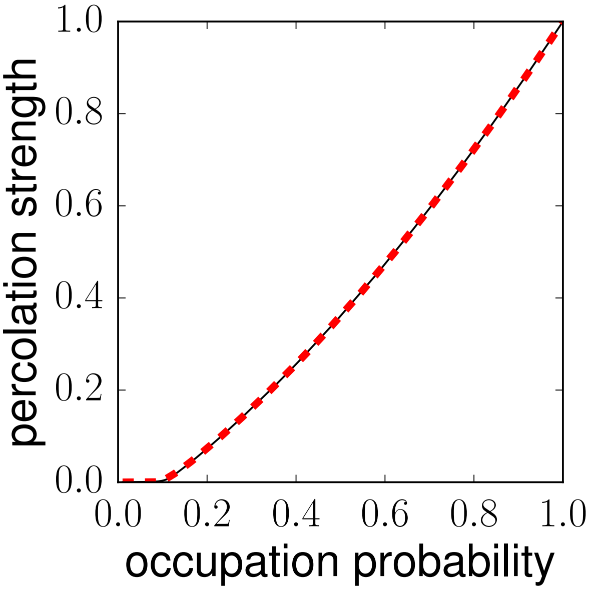

Supplementary Figure 77: Percolation diagram for the network `Gnutella, Aug. 30, 2002` [24, 29]. The black thin line represents the site percolation order parameter S as a function of the site occupation probability p. We calculate also the order parameter B for bond percolation and multiply it by p to obtain the red dashed line.

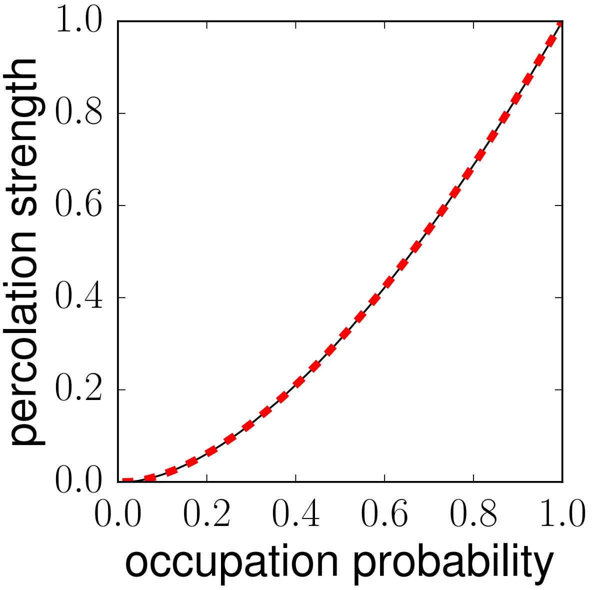

Supplementary Figure 78: Percolation diagram for the network `Slashdot` [11, 38]. The black thin line represents the site percolation order parameter S as a function of the site occupation probability p. We calculate also the order parameter B for bond percolation and multiply it by p to obtain the red dashed line.

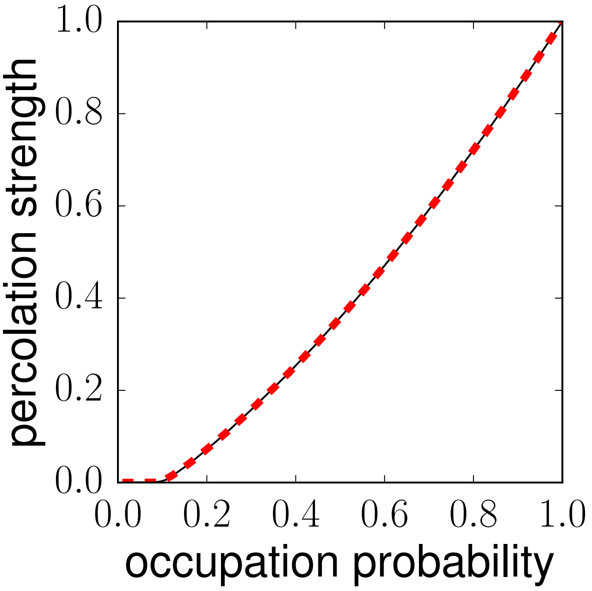

Supplementary Figure 79: Percolation diagram for the network `Gnutella, Aug. 31, 2002` [24, 29]. The black thin line represents the site percolation order parameter S as a function of the site occupation probability p. We calculate also the order parameter B for bond percolation and multiply it by p to obtain the red dashed line.

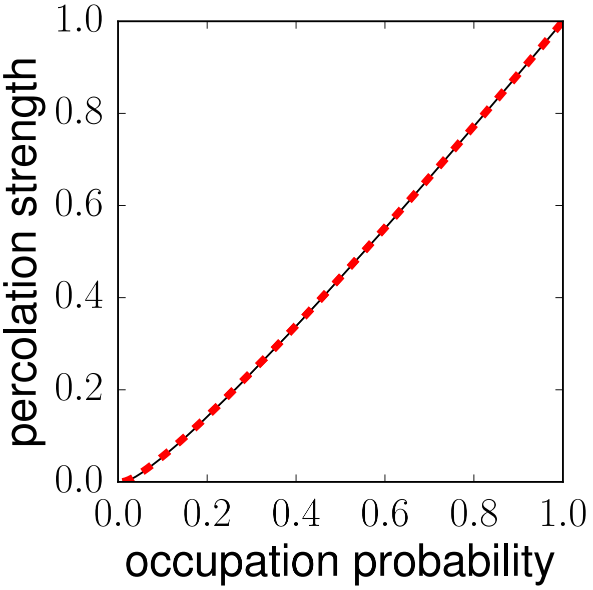

Supplementary Figure 80: Percolation diagram for the network `Facebook` [39]. The black thin line represents the site percolation order parameter S as a function of the site occupation probability p. We calculate also the order parameter B for bond percolation and multiply it by p to obtain the red dashed line.

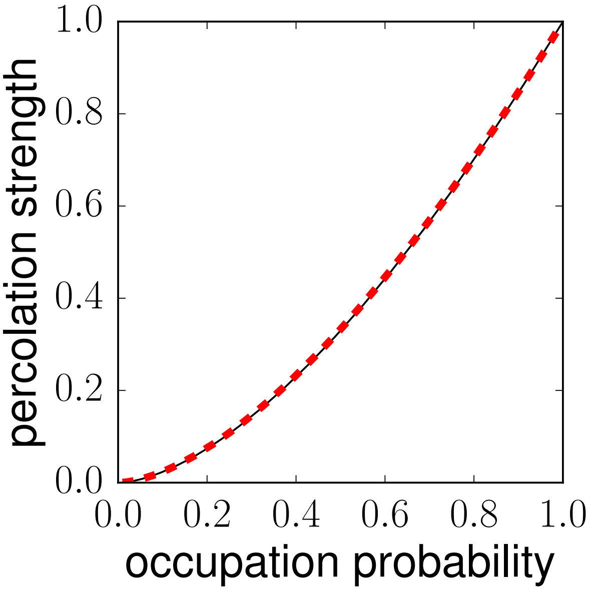

Supplementary Figure 81: Percolation diagram for the network `Epinions` [11, 40]. The black thin line represents the site percolation order parameter S as a function of the site occupation probability p. We calculate also the order parameter B for bond percolation and multiply it by p to obtain the red dashed line.

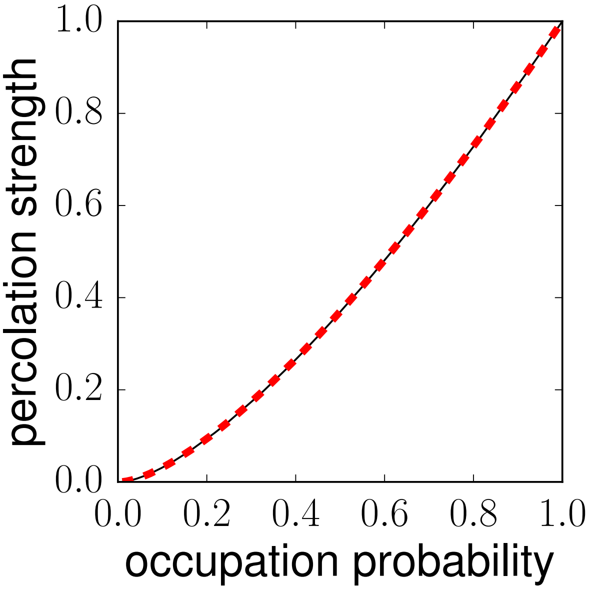

Supplementary Figure 82: Percolation diagram for the network `Slashdot zoo` [11, 41]. The black thin line represents the site percolation order parameter S as a function of the site occupation probability p. We calculate also the order parameter B for bond percolation and multiply it by p to obtain the red dashed line.

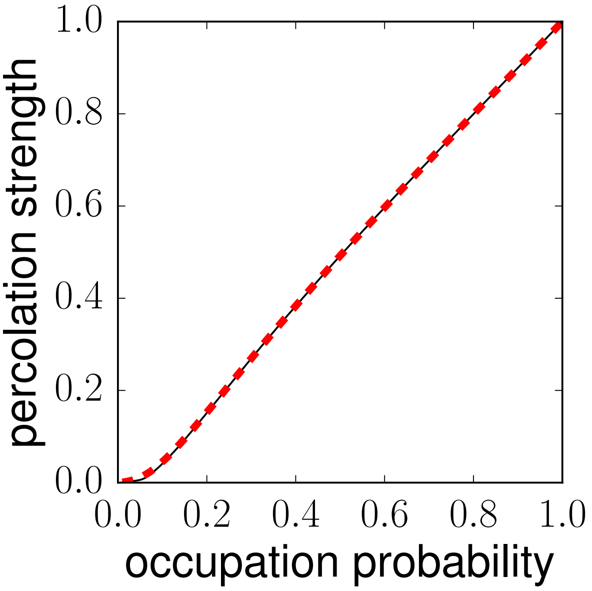

Supplementary Figure 83: Percolation diagram for the network `Flickr` [11, 42]. The black thin line represents the site percolation order parameter S as a function of the site occupation probability p. We calculate also the order parameter B for bond percolation and multiply it by p to obtain the red dashed line.

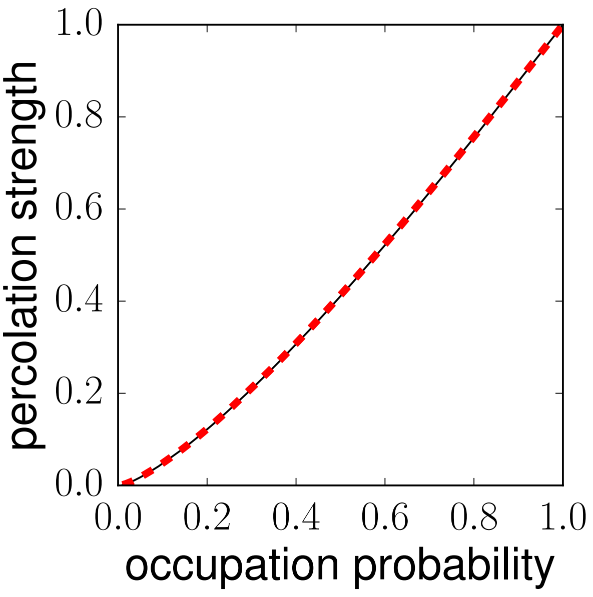

Supplementary Figure 84: Percolation diagram for the network `Wikipedia, edits` [11, 43]. The black thin line represents the site percolation order parameter S as a function of the site occupation probability p. We calculate also the order parameter B for bond percolation and multiply it by p to obtain the red dashed line.

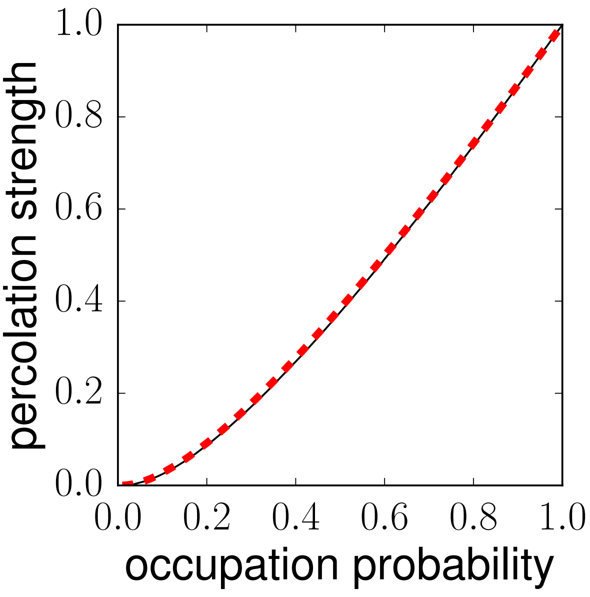

Supplementary Figure 86: Percolation diagram for the network `Gowalla` [11, 44]. The black thin line represents the site percolation order parameter S as a function of the site occupation probability p. We calculate also the order parameter B for bond percolation and multiply it by p to obtain the red dashed line.

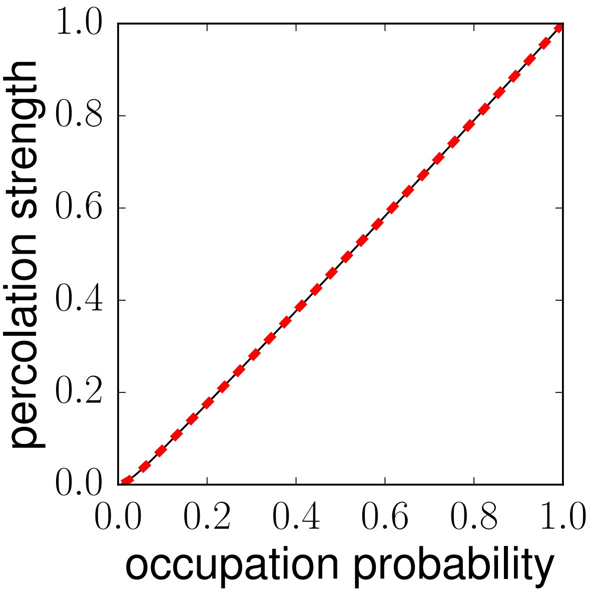

Supplementary Figure 85: Percolation diagram for the network `Petster, cats` [11]. The black thin line represents the site percolation order parameter S as a function of the site occupation probability p. We calculate also the order parameter B for bond percolation and multiply it by p to obtain the red dashed line.

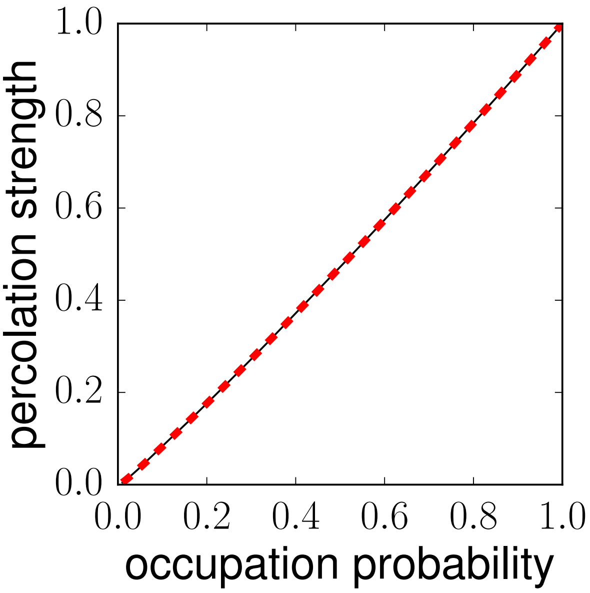

Supplementary Figure 87: Percolation diagram for the network `Libimseti` [11, 45, 46]. The black thin line represents the site percolation order parameter S as a function of the site occupation probability p. We calculate also the order parameter B for bond percolation and multiply it by p to obtain the red dashed line.

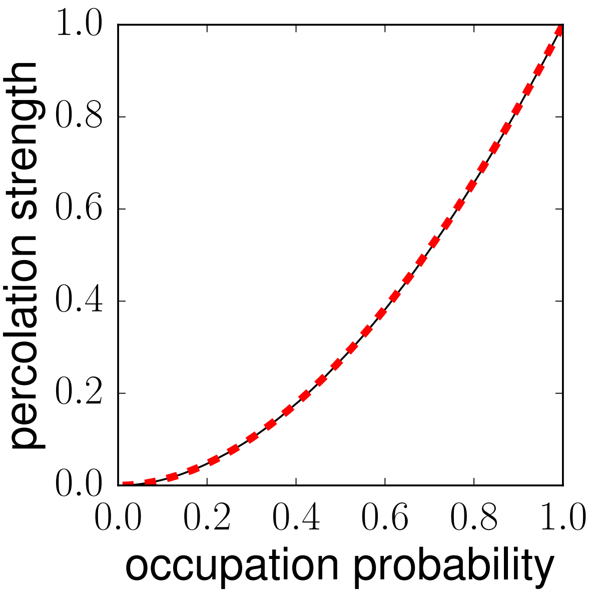

Supplementary Figure 88: Percolation diagram for the network `EU email` [11, 24]. The black thin line represents the site percolation order parameter S as a function of the site occupation probability p. We calculate also the order parameter B for bond percolation and multiply it by p to obtain the red dashed line.

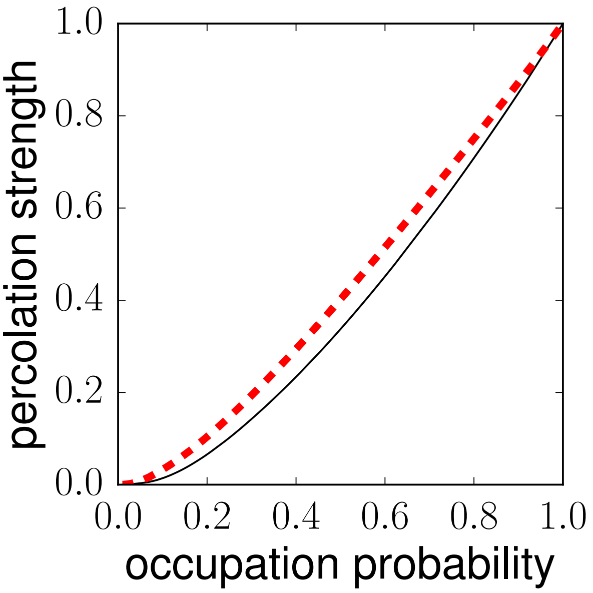

Supplementary Figure 89: Percolation diagram for the network `Web Stanford` [37]. The black thin line represents the site percolation order parameter S as a function of the site occupation probability p. We calculate also the order parameter B for bond percolation and multiply it by p to obtain the red dashed line.

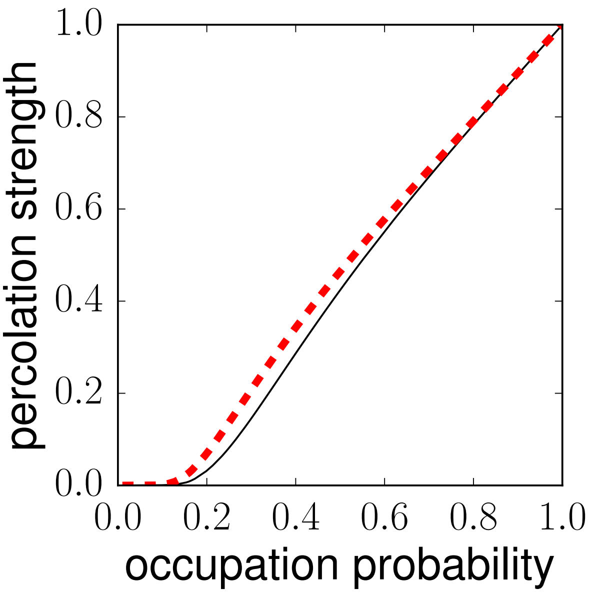

Supplementary Figure 90: Percolation diagram for the network `Amazon, Mar. 2, 2003` [47]. The black thin line represents the site percolation order parameter S as a function of the site occupation probability p. We calculate also the order parameter B for bond percolation and multiply it by p to obtain the red dashed line.

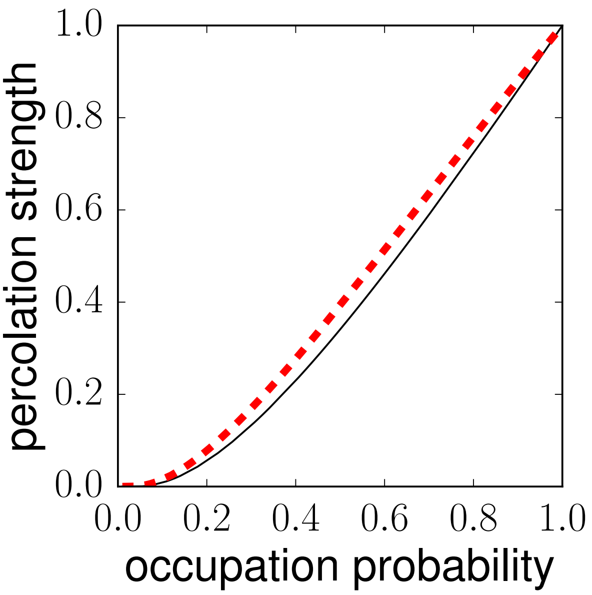

Supplementary Figure 91: Percolation diagram for the network `DBLP, collaborations` [11, 32]. The black thin line represents the site percolation order parameter S as a function of the site occupation probability p. We calculate also the order parameter B for bond percolation and multiply it by p to obtain the red dashed line.

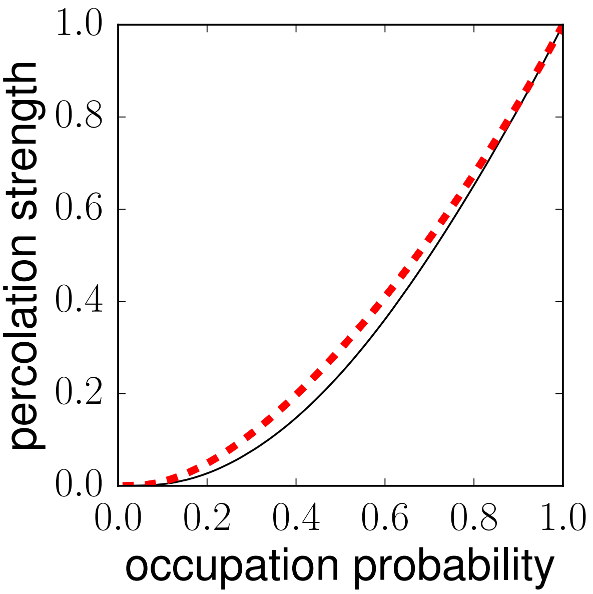

Supplementary Figure 92: Percolation diagram for the network `Web Notre Dame` [48]. The black thin line represents the site percolation order parameter S as a function of the site occupation probability p. We calculate also the order parameter B for bond percolation and multiply it by p to obtain the red dashed line.

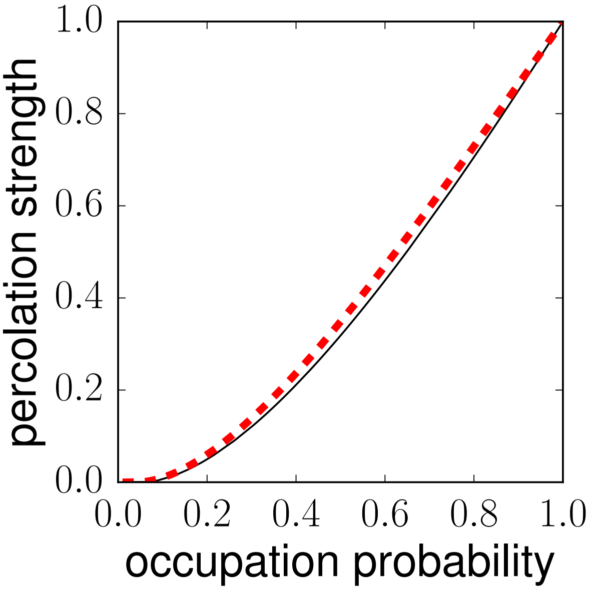

Supplementary Figure 93: Percolation diagram for the network `MathSciNet` [49]. The black thin line represents the site percolation order parameter S as a function of the site occupation probability p. We calculate also the order parameter B for bond percolation and multiply it by p to obtain the red dashed line.

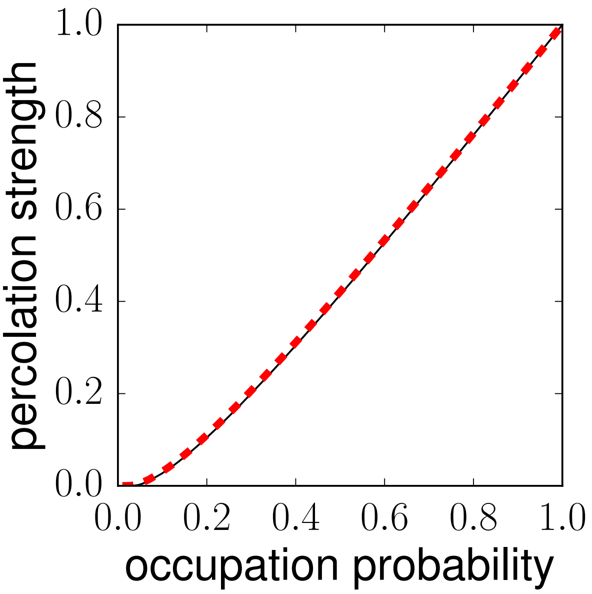

Supplementary Figure 94: Percolation diagram for the network `CiteSeer` [11, 50]. The black thin line represents the site percolation order parameter S as a function of the site occupation probability p. We calculate also the order parameter B for bond percolation and multiply it by p to obtain the red dashed line.

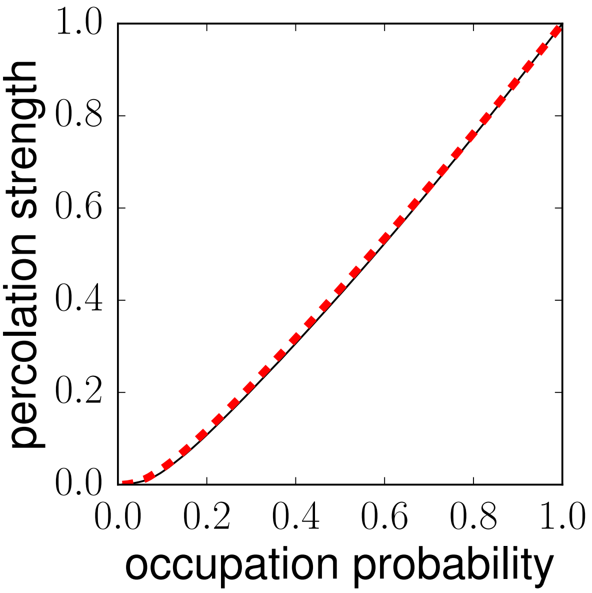

Supplementary Figure 95: Percolation diagram for the network `Zhishi` [11, 51]. The black thin line represents the site percolation order parameter S as a function of the site occupation probability p. We calculate also the order parameter B for bond percolation and multiply it by p to obtain the red dashed line.

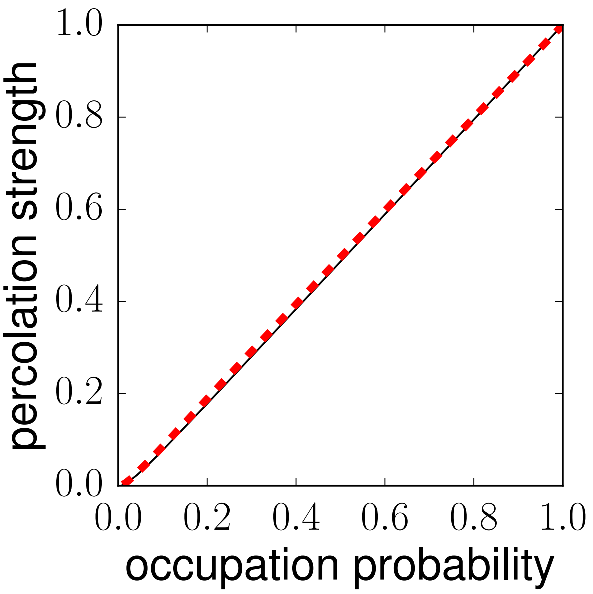

Supplementary Figure 96: Percolation diagram for the network `Actor coll. net.` [11, 52]. The black thin line represents the site percolation order parameter S as a function of the site occupation probability p. We calculate also the order parameter B for bond percolation and multiply it by p to obtain the red dashed line.

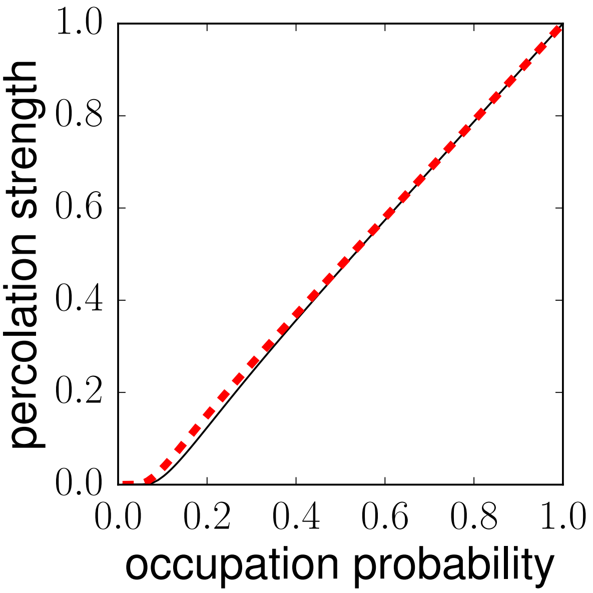

Supplementary Figure 97: Percolation diagram for the network `Amazon, Mar. 12, 2003` [47]. The black thin line represents the site percolation order parameter S as a function of the site occupation probability p. We calculate also the order parameter B for bond percolation and multiply it by p to obtain the red dashed line.

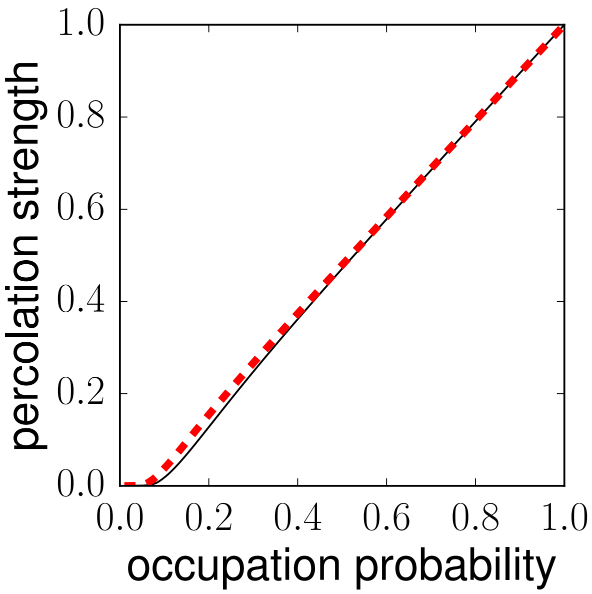

Supplementary Figure 98: Percolation diagram for the network `Amazon, Jun. 6, 2003` [47]. The black thin line represents the site percolation order parameter S as a function of the site occupation probability p. We calculate also the order parameter B for bond percolation and multiply it by p to obtain the red dashed line.

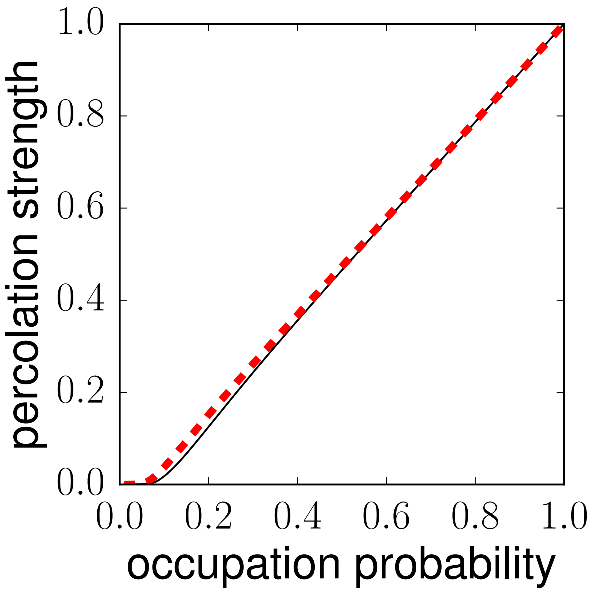

Supplementary Figure 99: Percolation diagram for the network `Amazon, May 5, 2003` [47]. The black thin line represents the site percolation order parameter S as a function of the site occupation probability p. We calculate also the order parameter B for bond percolation and multiply it by p to obtain the red dashed line.

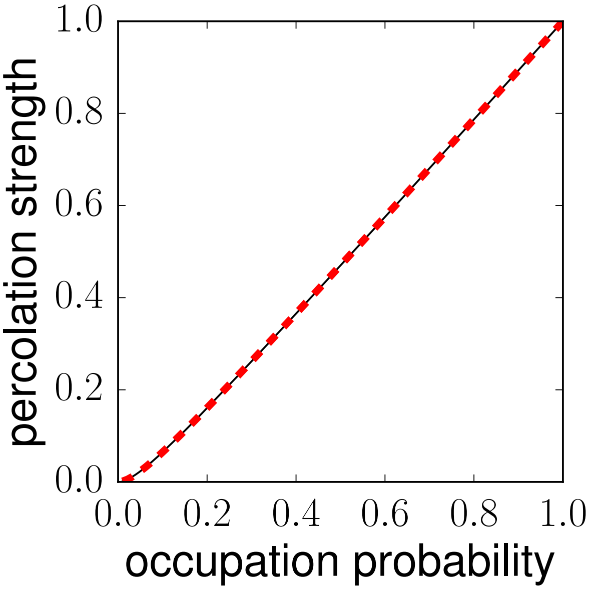

Supplementary Figure 100: Percolation diagram for the network `Petster, dogs` [11]. The black thin line represents the site percolation order parameter S as a function of the site occupation probability p. We calculate also the order parameter B for bond percolation and multiply it by p to obtain the red dashed line.

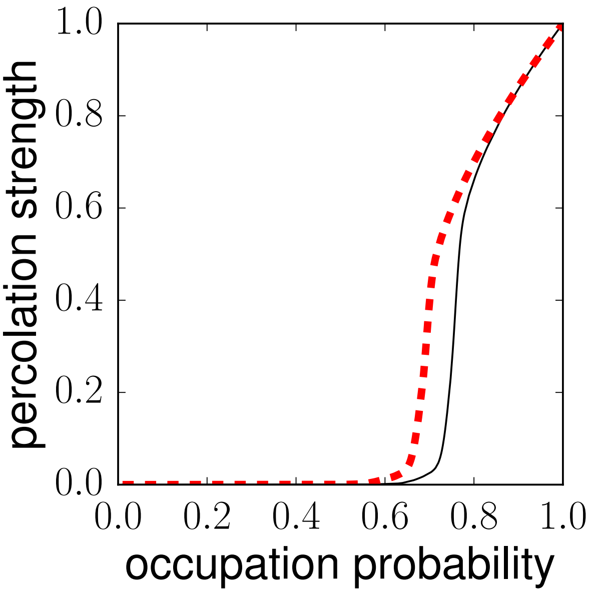

Supplementary Figure 101: Percolation diagram for the network `Road network PA` [37]. The black thin line represents the site percolation order parameter S as a function of the site occupation probability p. We calculate also the order parameter B for bond percolation and multiply it by p to obtain the red dashed line.

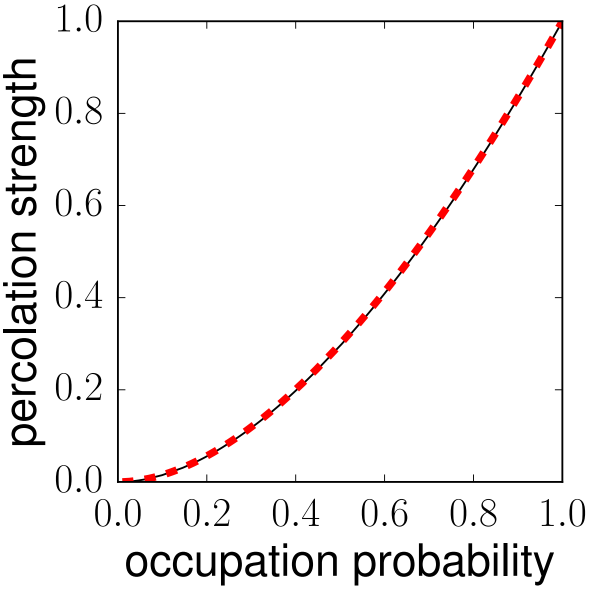

Supplementary Figure 102: Percolation diagram for the network `YouTube friend. net.` [11, 53]. The black thin line represents the site percolation order parameter S as a function of the site occupation probability p. We calculate also the order parameter B for bond percolation and multiply it by p to obtain the red dashed line.

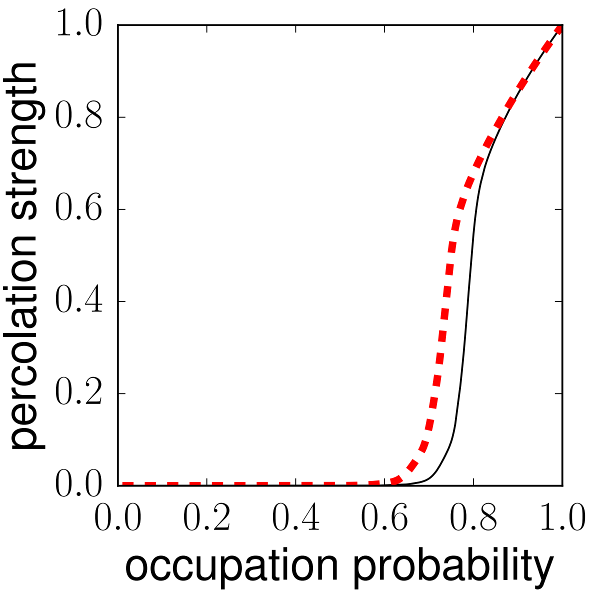

Supplementary Figure 103: Percolation diagram for the network `Road network TX` [37]. The black thin line represents the site percolation order parameter S as a function of the site occupation probability p. We calculate also the order parameter B for bond percolation and multiply it by p to obtain the red dashed line.

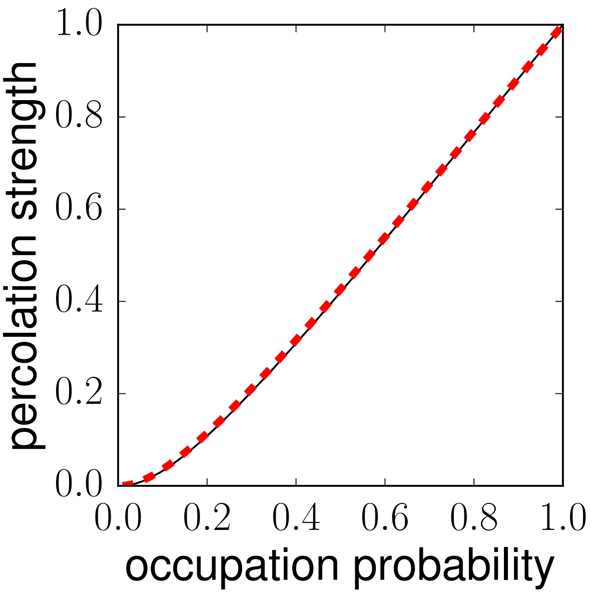

Supplementary Figure 104: Percolation diagram for the network `AS Skitter` [30]. The black thin line represents the site percolation order parameter S as a function of the site occupation probability p. We calculate also the order parameter B for bond percolation and multiply it by p to obtain the red dashed line.

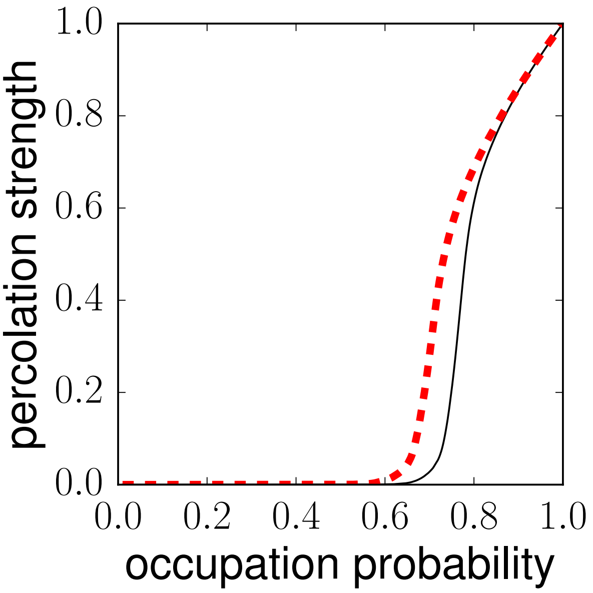

Supplementary Figure 105: Percolation diagram for the network `Road network CA` [37]. The black thin line represents the site percolation order parameter S as a function of the site occupation probability p. We calculate also the order parameter B for bond percolation and multiply it by p to obtain the red dashed line.

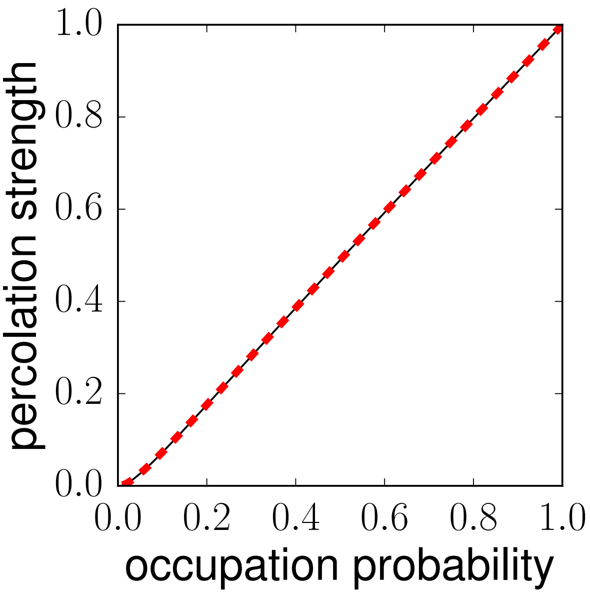

Supplementary Figure 106: Percolation diagram for the network `Wikipedia, pages` [49]. The black thin line represents the site percolation order parameter S as a function of the site occupation probability p. We calculate also the order parameter B for bond percolation and multiply it by p to obtain the red dashed line.

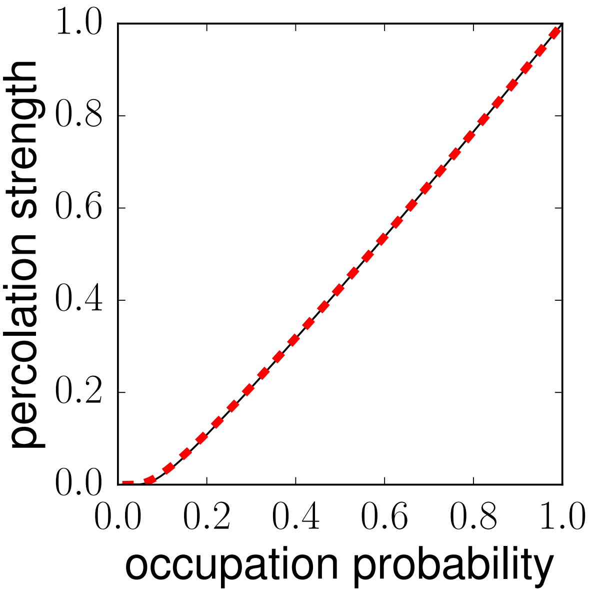

Supplementary Figure 107: Percolation diagram for the network `US Patents` [11, 54]. The black thin line represents the site percolation order parameter S as a function of the site occupation probability p. We calculate also the order parameter B for bond percolation and multiply it by p to obtain the red dashed line.

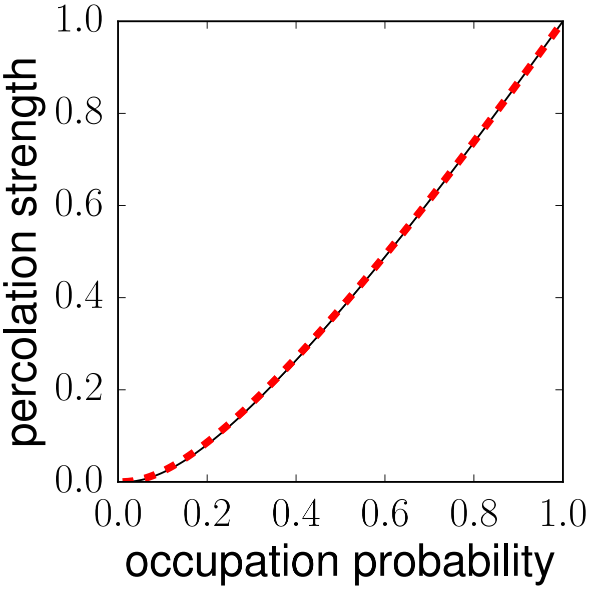

Supplementary Figure 108: Percolation diagram for the network `DBpedia` [11, 55]. The black thin line represents the site percolation order parameter S as a function of the site occupation probability p. We calculate also the order parameter B for bond percolation and multiply it by p to obtain the red dashed line.

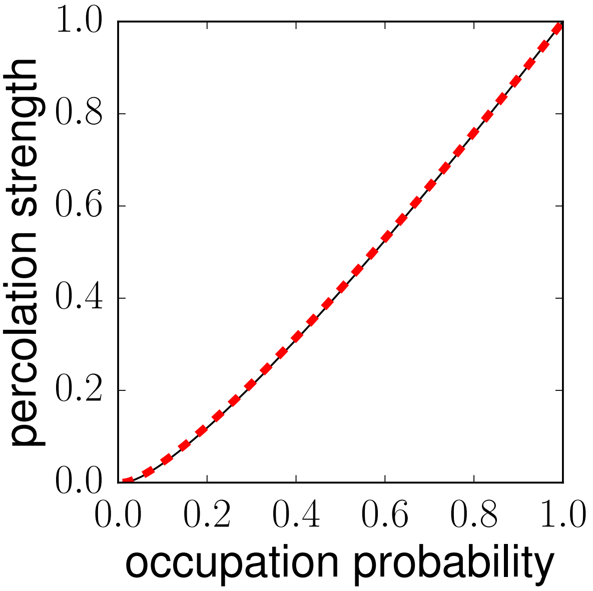

Supplementary Figure 109: Percolation diagram for the network `LiveJournal` [11, 56]. The black thin line represents the site percolation order parameter S as a function of the site occupation probability p. We calculate also the order parameter B for bond percolation and multiply it by p to obtain the red dashed line.

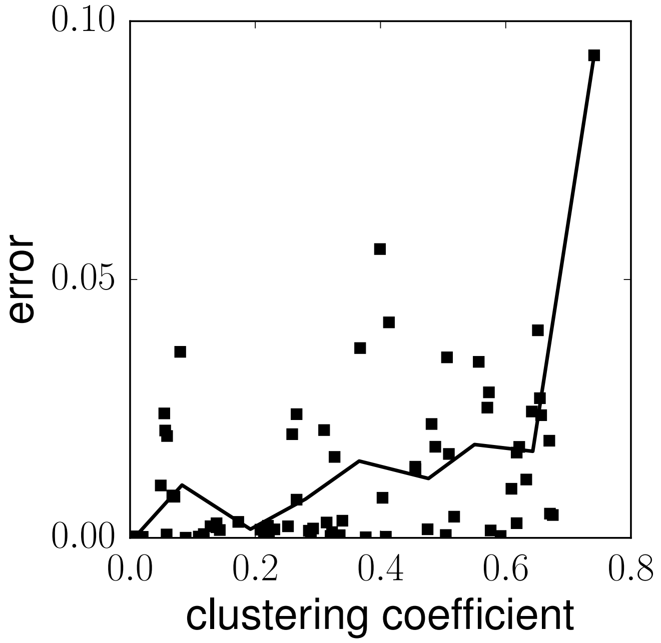

Supplementary Figure 110: Quantitative test of the equation $S = pB$ in real networks. We measure the error as $V = \int dp\, |S(p) - pB(p)|$, i.e., the area laying between the curves S and pB as obtained from numerical simulations (see Figs. 1- 109). V is then plotted against the average clustering coefficient C of the network, used as a proxy for the extent to which the locally tree-like approximation holds. The clustering coefficient of the node i is computed as $C_i = \frac{\sum_{r,s} A_{i,r} A_{i,s} A_{r,s}}{\sum_{r,s} A_{i,r} A_{i,s}}$, with A adjacency matrix of the network. The clustering coefficient of the network is then estimated as the average value over all nodes, that is $C = 1/N \sum_i C_i$. The black line represents the average value of V at different level of clustering, and serves as a guide for the global behavior of V as a function of C. The black line is generated according to the following procedure. We divide the range of possible values of C in nine equally spaced bins. We then estimate the average value of the error V in each bin, and the average value of the cluster coefficient within each bin. The black line is finally obtained connecting these points.

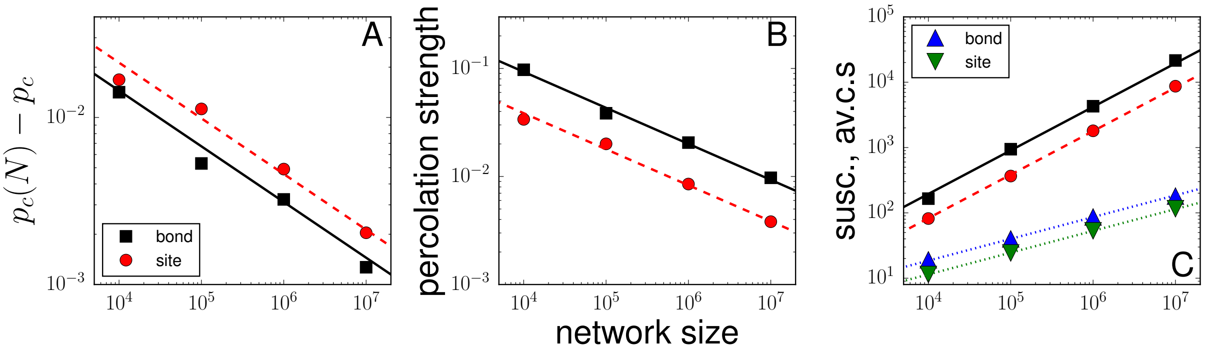

Supplementary Figure 111: Critical exponents of bond (black squares) and site (red circles) percolation on Erdős-Rényi graphs. Results are obtained by setting the average degree $\langle k \rangle = 4.0$. **A** Best estimate of the pseudo-critical critical point $p_c(N)$ for different network sizes N. Numerical results are compared with the expected power-law decay towards p_c, with $p_c = 1/\langle k \rangle$ and decay exponent $\nu = 3$ (full black and dashed red lines). **B** Order parameter at pseudo-criticality as a function of the network size. The full black and the dashed red lines decay with exponent $\beta_b/\nu = \beta_s/\nu = 1/3$ as N grows. **C** Maximal values of the susceptibility as a function of N (black squares for bond percolation, and red circles for site percolation). The full black and the dashed red lines increase as $N^{1-\beta/\nu}$ ($\beta_b = \beta_s = \beta$), thus as $N^{2/3}$. Average cluster size for bond (blue triangles) and site (green triangles) at pseudo-criticality as a function of N. The dotted lines increase as $N^{1-2\beta/\nu}$, thus as $N^{1/3}$.

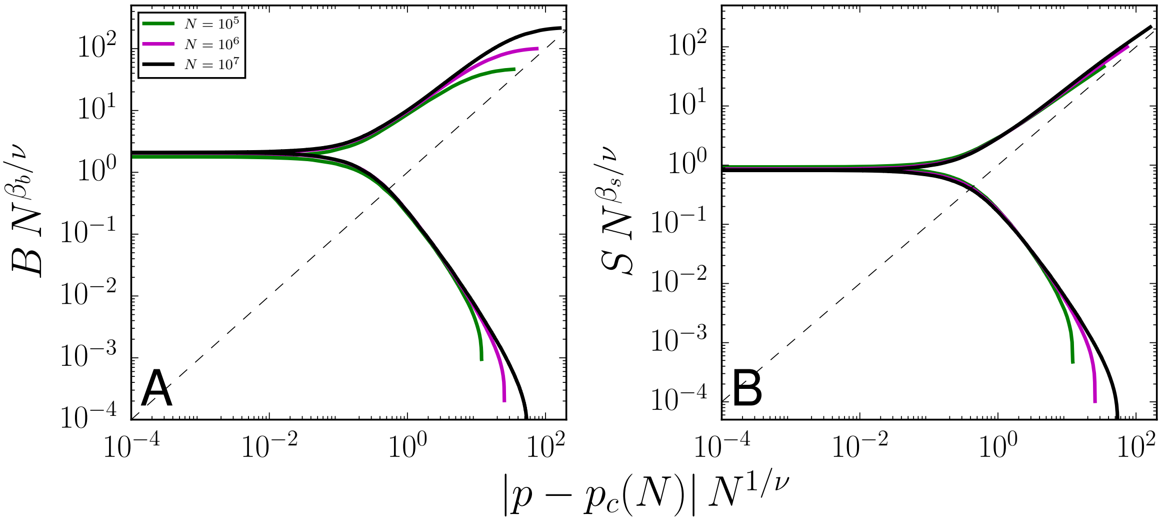

Supplementary Figure 112: Finite-size scaling for percolation on Erdős-Rényi graphs. We analyzed the same networks as in Fig. 111. The average degree is $\langle k \rangle = 4.0$. **A** Collapse between the order parameters B of the bond percolation model in networks with different sizes. The collapse is obtained by setting $\beta_b/\nu = 1/3$ and $\nu = 3$. The dashed line corresponds to a power-law with exponent equal to $\beta_b = 1$. **B** Same as in panel A, but for the site percolation model. The critical exponent used in the scaling are $\beta_s = \beta_b = 1$ and $\nu = 3$.

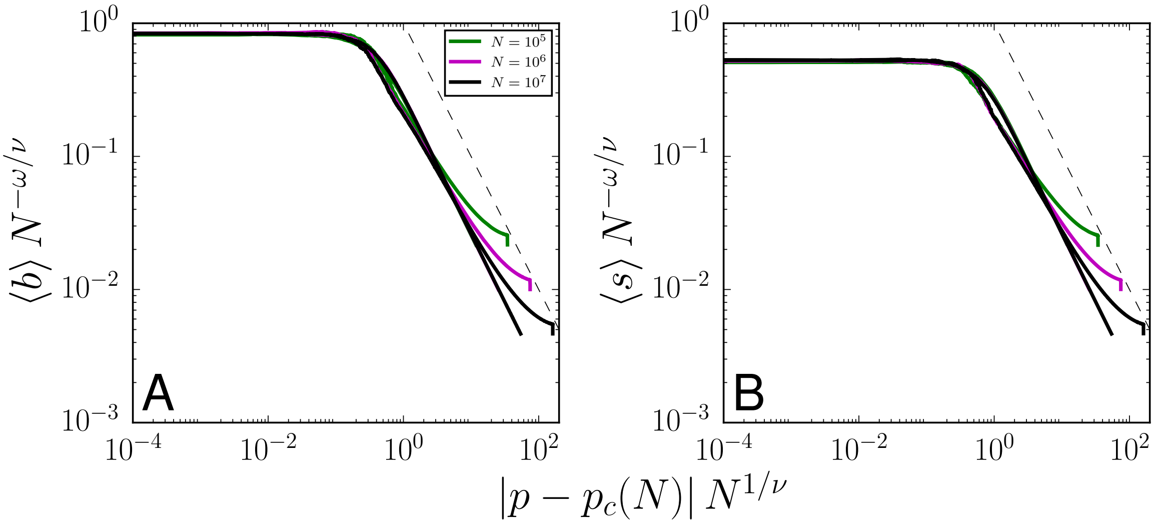

Supplementary Figure 113: Finite-size scaling for percolation on Erdős-Rényi graphs. We analyzed the same networks as in Figs. 111 and 112. The average degree is $\langle k \rangle = 4.0$. **A** Collapse between the average size of finite clusters $\langle b \rangle$ of the bond percolation model in networks with different sizes. The collapse is obtained by setting $\omega = 1$ and $\nu = 3$. The dashed line corresponds to a power-law with exponent equal to ω/ν. **B** Same as in panel A, but for the site percolation model. The critical exponent used in the scaling are identical to those used in panel A.

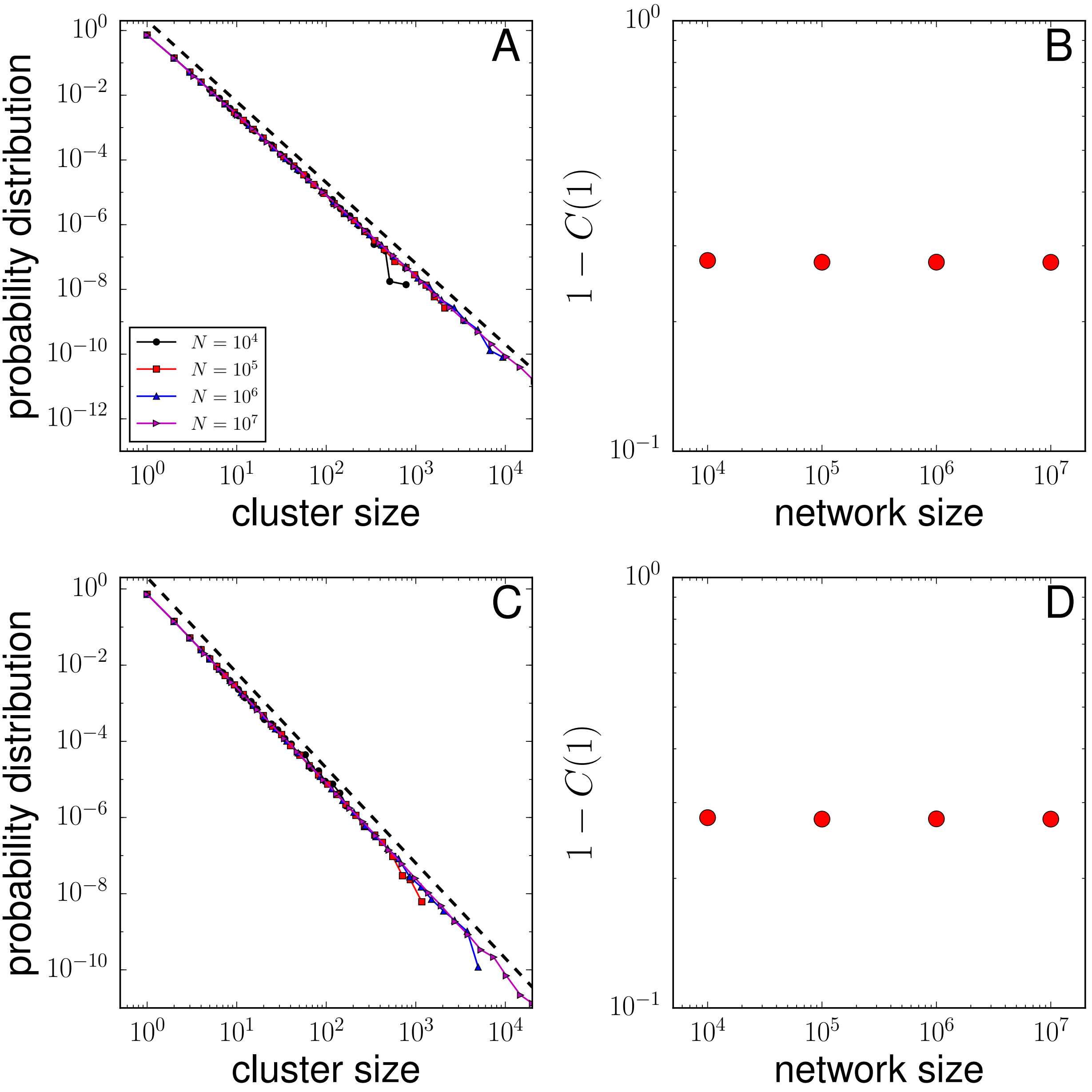

Supplementary Figure 114: Distribution of finite-cluster sizes for percolation on Erdős-Rényi graphs. We analyzed the same networks as in Figs. 111, 112, and 113. The average degree is $\langle k \rangle = 4.0$. **A** Probability distribution to observe a cluster of a given size in the bond percolation model for $p = p_c(N)$. Each curve corresponds to a different network size. The tail of the various distributions decays as a power-law for large values of the cluster sizes with exponent compatible with 5/2 (dashed line). **B** Weight of clusters of size one, $C(1)$, in the distribution of finite-cluster sizes. As the system size grows, $C(1)$ tends to an asymptotic value clearly smaller than one. **C and D** Same as in panels A and B, but for the site percolation model.

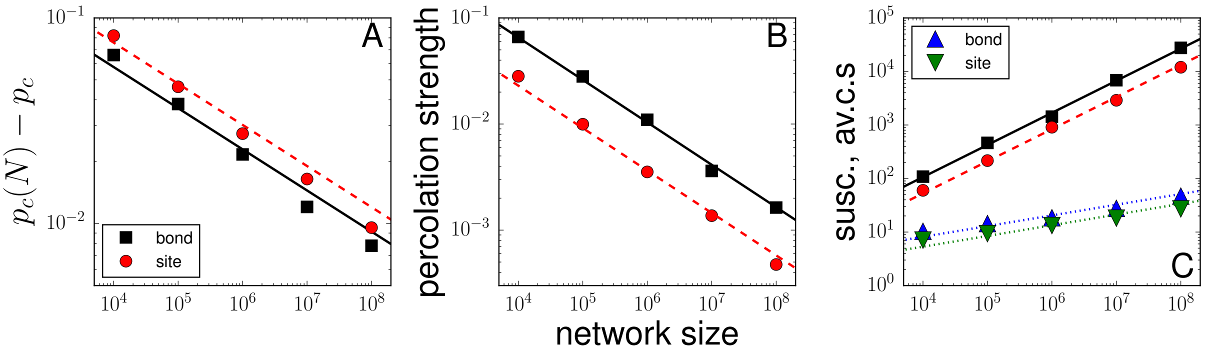

Supplementary Figure 115: Critical exponents of bond (black squares) and site (red circles) percolation on scale-free graphs. Results are obtained by setting the degree exponent $\gamma = 3.5$. **A** Best estimate of the pseudo-critical critical point $p_c(N)$ for different network sizes N. Numerical results are compared with the expected power-law decay towards p_c, with $p_c = \sum_{k=3}^{\sqrt{N}} k^{1-\gamma}/(\sum_{k=3}^{\sqrt{N}} k^{2-\gamma} - \sum_{k=3}^{\sqrt{N}} k^{1-\gamma})$ and decay exponent $\nu = (\gamma-1)/(\gamma-3)$ (full black and dashed red lines). **B** Order parameter at pseudo-criticality as a function of the network size. The full black and the dashed red lines decay with exponent $\beta/\nu = 1/(\gamma-1)$ as N grows ($\beta_b = \beta_s = \beta$). **C** Maximal values of the suceptibility as a function of N. The full black and the dashed red lines increase as $N^{1-\beta/\nu}$, thus as $N^{(\gamma-2)/(\gamma-1)}$. Average cluster size for bond (blue triangles) and site (green triangles) percolation at pseudo-criticality as a function of N. The dotted lines increase as $N^{1-2\beta/\nu}$, thus as $N^{(\gamma-3)/(\gamma-1)}$.

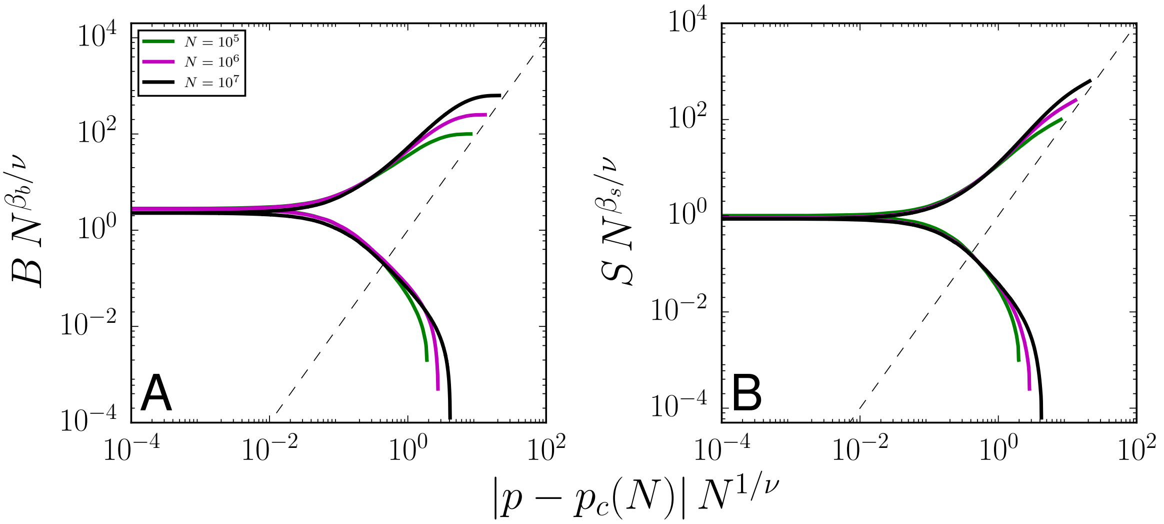

Supplementary Figure 116: Finite-size scaling for percolation on scale-free graphs. We analyzed the same networks as in Fig. 115. The degree exponent is $\gamma = 3.5$. **A** Collapse between the order parameters B of the bond percolation model in networks with different sizes. The collapse is obtained by setting $\beta_b/\nu = 1/(\gamma-1)$ and $\nu = (\gamma-1)/(\gamma-3)$. The dashed line corresponds to a power-law with exponent equal to $\beta_b = 1/(\gamma-3)$. **B** Same as in panel A, but for the site percolation model. The critical exponent used in the scaling are $\beta_s = \beta_b = 1/(\gamma-3)$ and $\nu = (\gamma-1)/(\gamma-3)$.

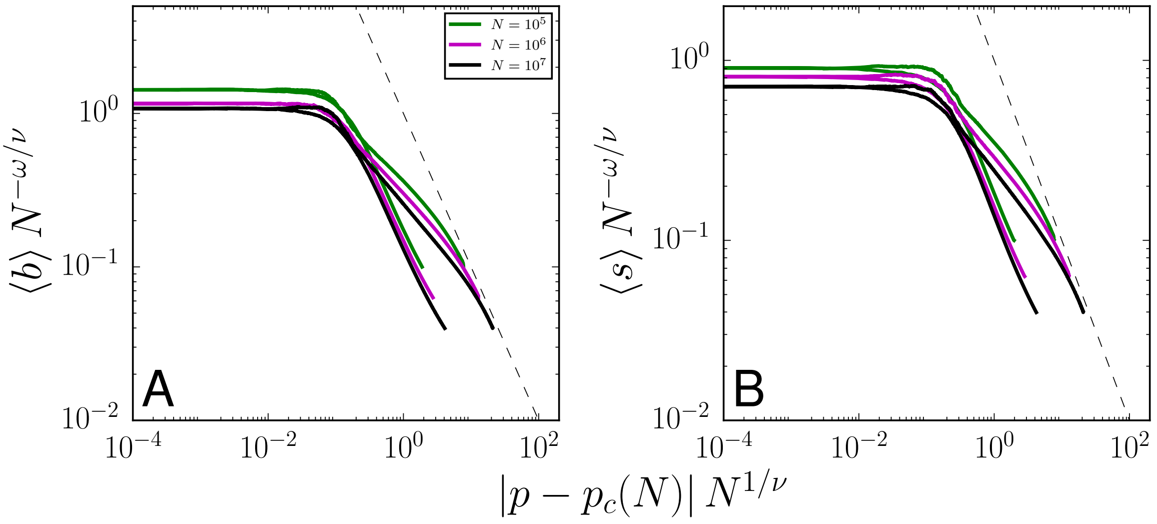

Supplementary Figure 117: Finite-size scaling for percolation on scale-free graphs. We analyzed the same networks as in Figs. 115 and 116. The degree exponent is $\gamma = 3.5$. **A** Collapse between the average size of finite clusters $\langle b \rangle$ of the bond percolation model in networks with different sizes. The collapse is obtained by setting $\omega = 1$ and $\nu = (\gamma - 1)/(\gamma - 3)$. The dashed line corresponds to a power-law with exponent equal to ω/ν. **B** Same as in panel A, but for the site percolation model. The critical exponent used in the scaling are identical to those used in panel A.

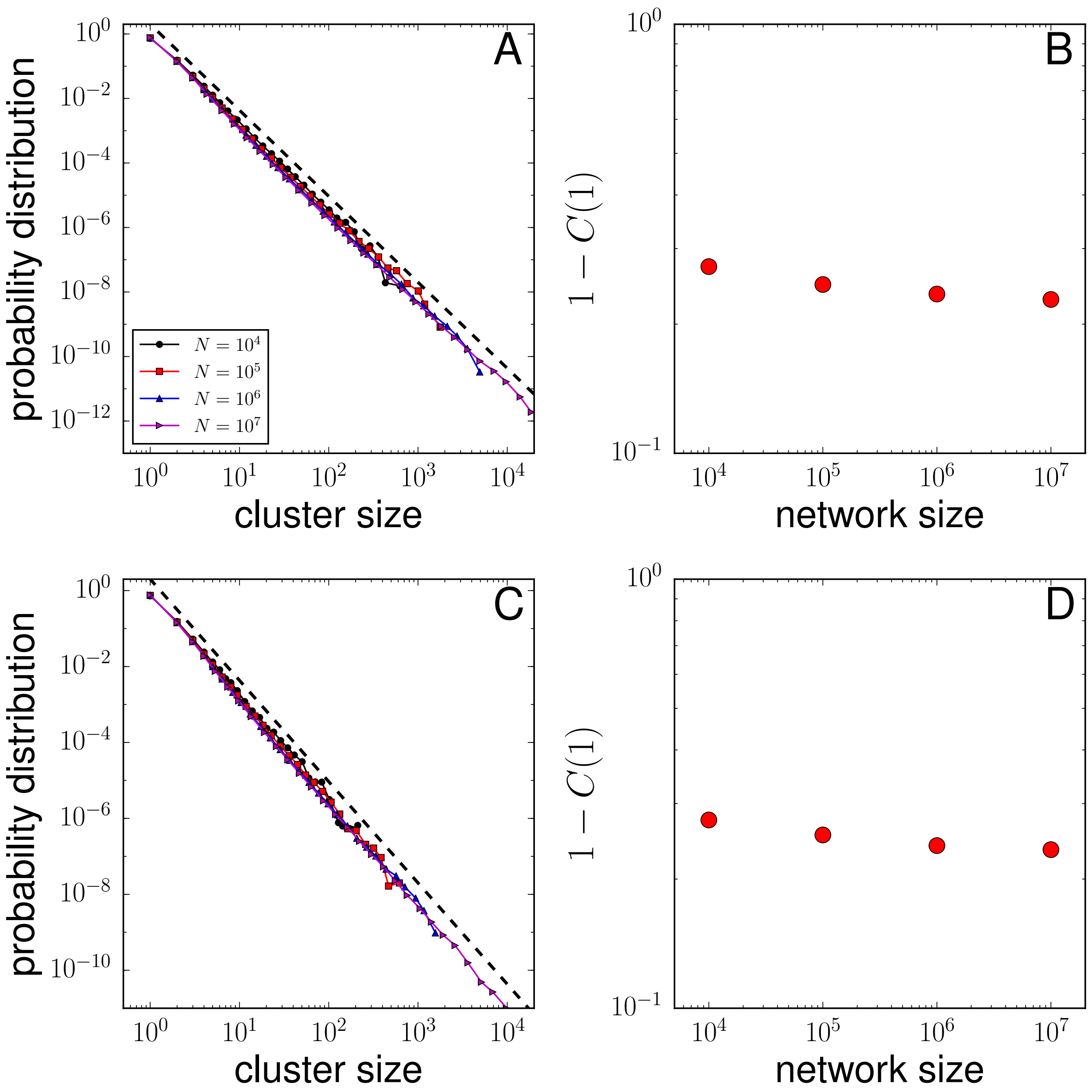

Supplementary Figure 118: Distribution of finite-cluster sizes in scale-free graphs. We analyzed the same networks as in Figs. 115, 116, and 117. The degree exponent is $\gamma = 3.5$. **A** Probability distribution to observe a cluster of a given size in the bond percolation model for $p = p_c(N)$. Each curve corresponds to a different network size. The tail of the various distributions decays as a power-law for large values of the cluster sizes with exponent compatible with $(2\gamma - 3)/(\gamma - 2)$ (dashed line). **B** Weight of clusters of size one, $C(1)$, in the distribution of finite-cluster sizes. As the system size grows, $C(1)$ tends to an asymptotic value clearly smaller than one. **C and D** Same as in panels A and B, but for the site percolation model.

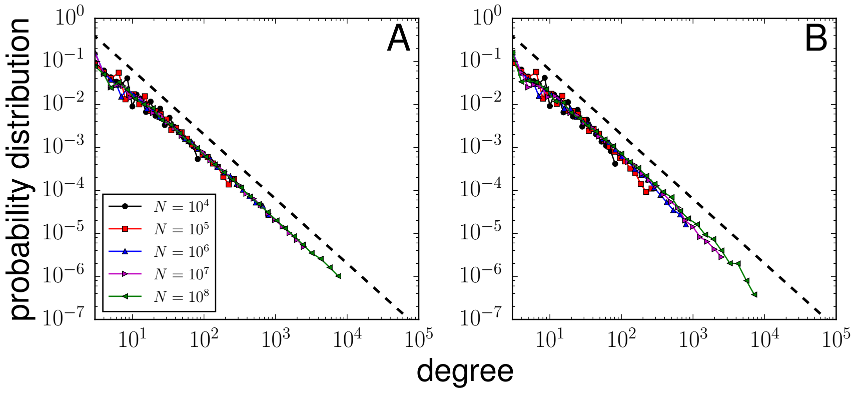

Supplementary Figure 119: Degree distribution at the pseudo-critical point $p_c(N)$. Different colors and symbols correspond to different network sizes N. The degree distribution of the entire network is $P(k) \sim k^{-\gamma}$ if $k \in [3, \sqrt{N}]$, and $P(k) = 0$, otherwise. In this analysis, the degree exponent is $\gamma = 2.5$. The degree distribution of the largest cluster is compatible instead with $P(k) \sim k^{-\gamma+1}$ if $k \in [3, \sqrt{N}]$, and $P(k) = 0$, otherwise, as emphasized by the good agreement of the scaling of the empirical points with the dashed lines. The histograms are computed over at least 10 independent realizations of the percolation model on the same network. **A** Results for the bond percolation model. **B** Results for the site percolation model.

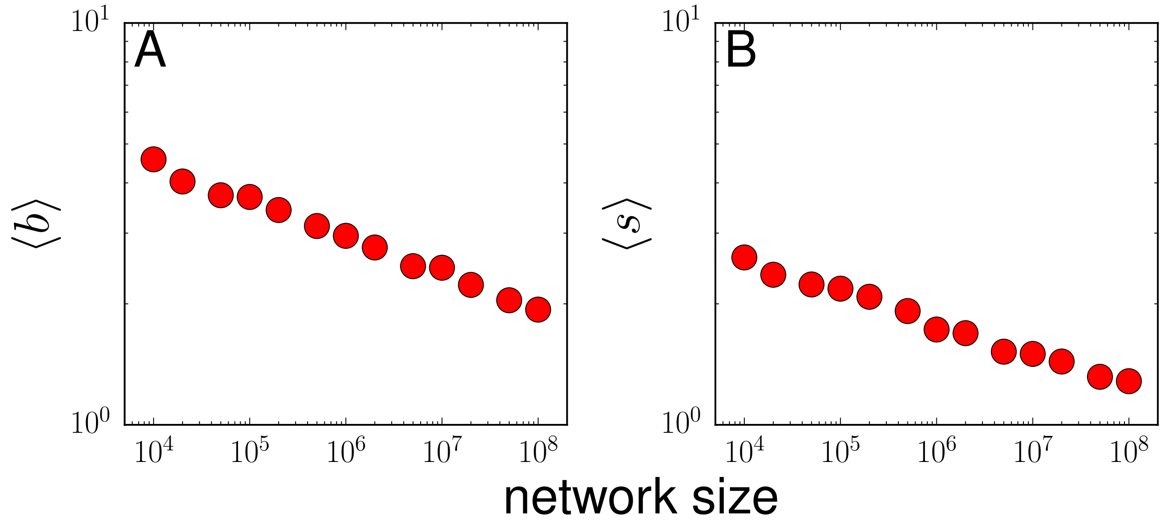

Supplementary Figure 120: Average cluster size in scale-free graphs. We analyzed the same networks as in Figs. 1, 2, 3 of the main text. The degree exponent is $\gamma = 2.5$. **A** Average size of finite clusters $\langle b \rangle$ at pseudo-criticality as a function of the network size N. **B** Same as in panel A, but for the site percolation model.

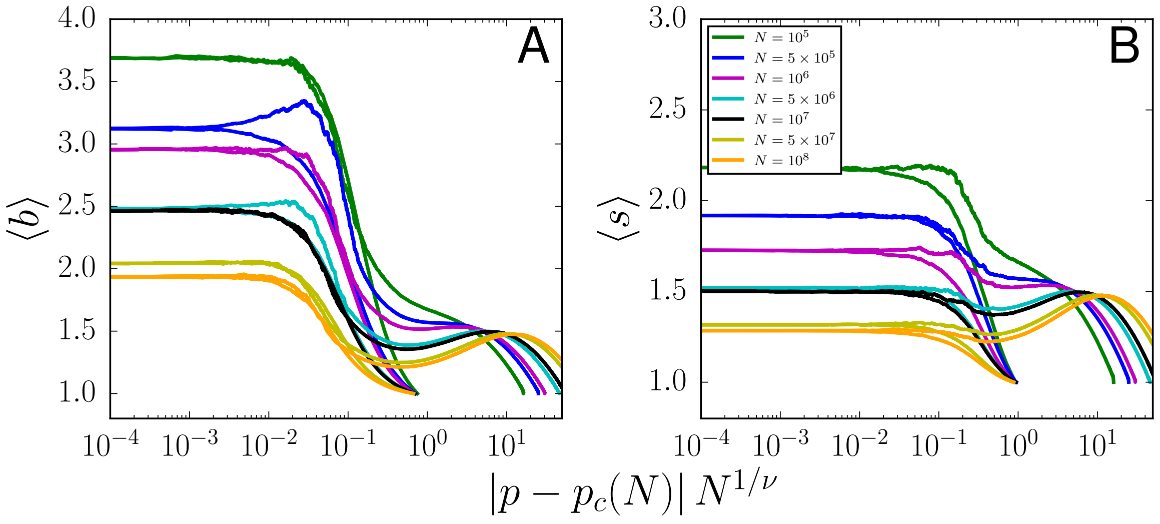

Supplementary Figure 121: Finite-size scaling in scale-free graphs. We analyzed the same networks as in Figs. 1, 2 and 3 of the main text. The degree exponent is $\gamma = 2.5$. **A** Average size of finite clusters $\langle b \rangle$ of the bond percolation model in networks with different sizes. We set $\nu = 2/(\gamma - 3)$. **B** Same as in panel A, but for the site percolation model. In addition to a lack of power-law scaling of the peak observable for $p = p_c(N)$, we also note the presence of an additional anomalous peak located at $p \simeq 0.1$.

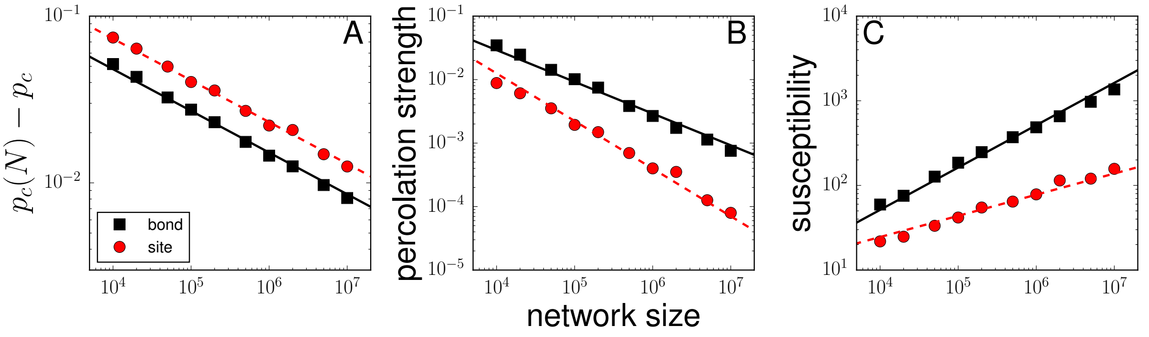

Supplementary Figure 122: Critical exponents of bond (black squares) and site (red circles) percolation on scale-free graphs with clustering. Results are obtained on networks generated according to the reciped proposed by Newman [57]. In the specific case here, we assigned every node to a number t of triangles, with t random integer number extracted from the power-law distribution $P(t) \sim t^{-\gamma}$ if $t \in [2, \sqrt{N}]$, and $P(t) = 0$, otherwise. We set $\gamma = 2.5$. The procedure generates networks with power-law degree distribution $P(k) \sim k^{-\gamma}$, and average clustering coefficient $C \simeq 0.24$ for all sizes N. **A** Best estimate of the pseudo-critical critical point $p_c(N)$ for different network sizes N. Numerical results are compared with the expected power-law decay towards $p_c = 0$, and decay exponent $1/\nu = (3 - \gamma)/2$ (full black and dashed red lines). **B** Order parameter at pseudo-criticality as a function of the network size. The full black line decays with exponent $\beta_b/\nu = 1/2$ as N grows, while the dashed red line corresponds to the exponent $\beta_s/\nu = (4 - \gamma)/2$. **C** Maximal values of the suceptibility as a function of N. The full black line increases as as $N^{1-\beta_b/\nu}$, thus as $N^{1/2}$. The dashed red line corresponds to the exponent $1 - \beta_s/\nu = (\gamma - 2)/2$.

Supplementary Tables

network	N	E	p_c	q_c	C	V	Fig.	Refs.	Url
Social 3	32	80	0.2500	0.3125	0.3266	0.0157	1	[1]	url
Karate club	34	78	0.2436	0.3824	0.5706	0.0252	2	[2]	url
Protein 2	53	123	0.3902	0.6038	0.4135	0.0417	3	[1]	url
Dolphins	62	159	0.2390	0.3871	0.2590	0.0201	4	[3]	url
Social 1	67	142	0.3099	0.4030	0.3099	0.0209	5	[1]	url
Les Miserables	77	254	0.1339	0.3377	0.5731	0.0282	6	[4]	url
Protein 1	95	213	0.6056	0.8000	0.3991	0.0559	7	[1]	url
E. Coli, transcription	97	212	0.5189	0.6495	0.3675	0.0367	8	[5]	url
Political books	105	441	0.1927	0.3429	0.4875	0.0176	9	[6]	url
David Copperfield	112	425	0.1035	0.1786	0.1728	0.0031	10	[7]	url
College football	115	613	0.1338	0.2087	0.4032	0.0078	11	[8]	url
S 208	122	189	0.4656	0.5492	0.0591	0.0197	12	[1]	url
High school, 2011	126	1,709	0.0380	0.0873	0.5759	0.0014	13	[9]	url
Bay Dry	128	2,106	0.0304	0.0469	0.3346	0.0005	14	[10, 11]	url
Bay Wet	128	2,075	0.0308	0.0469	0.3346	0.0005	15	[11]	url
Radoslaw Email	167	3,250	0.0200	0.0419	0.5919	0.0004	16	[11, 12]	url
High school, 2012	180	2,220	0.0437	0.0944	0.4752	0.0017	17	[9]	url
Little Rock Lake	183	2,434	0.0300	0.0874	0.3226	0.0011	18	[11, 13]	url
Jazz	198	2,742	0.0314	0.0859	0.6175	0.0028	19	[14]	url
S 420	252	399	0.4511	0.5357	0.0561	0.0208	20	[1]	url
C. Elegans, neural	297	2,148	0.0540	0.1044	0.2924	0.0018	21	[15]	url
Network Science	379	914	0.3982	0.6359	0.7412	0.0934	22	[7]	url
Dublin	410	2,765	0.0778	0.1951	0.4558	0.0133	23	[11, 16]	url
US Air Trasportation	500	2,980	0.0268	0.1080	0.6175	0.0165	24	[17]	url
S 838	512	819	0.4493	0.5449	0.0547	0.0241	25	[1]	url
Yeast, transcription	662	1,062	0.2458	0.3414	0.0490	0.0101	26	[18]	url
URV email	1,133	5,451	0.0646	0.1068	0.2202	0.0023	27	[19]	url
Political blogs	1,222	16,714	0.0168	0.0442	0.3203	0.0004	28	[6]	url
Air traffic	1,226	2,408	0.1815	0.2871	0.0675	0.0082	29	[11]	url
Yeast, protein	1,458	1,948	0.2988	0.3752	0.0708	0.0080	30	[20]	url
Petster, hamster	1,788	12,476	0.0273	0.0587	0.1433	0.0015	31	[11]	url
UC Irvine	1,893	13,835	0.0248	0.0449	0.1097	0.0002	32	[11, 21]	url
Yeast, protein	2,224	6,609	0.0794	0.1245	0.1381	0.0028	33	[22]	url
Japanese	2,698	7,995	0.0318	0.1160	0.2196	0.0003	34	[1]	url
Open flights	2,905	15,645	0.0199	0.0792	0.4555	0.0138	35	[11, 23]	url
GR-QC, 1993-2003	4,158	13,422	0.1345	0.2244	0.5569	0.0341	36	[24]	url
Tennis	4,338	81,865	0.0073	0.0274	0.2888	0.0001	37	[25]	url
US Power grid	4,941	6,594	0.6583	0.7353	0.0801	0.0360	38	[15]	url
HT09	5,352	18,481	0.0283	0.1700	0.0087	0.0002	39	[16]	url
Hep-Th, 1995-1999	5,835	13,815	0.1081	0.1973	0.5062	0.0349	40	[26]	url

Supplementary Table 1: Analysis of real networks. From left to right, we report: name of the network, number of nodes in the giant component, number of edges in the giant component, best estimate of the bond percolation threshold, best estimate of the site percolation threshold, clustering coefficient, error associated to the relation $S = pB$ (see caption of Supplementary Fig. 110 for definition), number of figure showing the percolation diagram of the network, references of the paper(s) where the network was analyzed, and url where network data have been dowloaded (clicking on the world "url" automatically opens the corresponding webpage in the browser).

network	N	E	p_c	q_c	C	V	Fig.	Refs.	Url
Reactome	5,973	145,778	0.0114	0.0591	0.6091	0.0095	41	[11, 27]	url
Jung	6,120	50,290	0.0093	0.1239	0.6752	0.0044	42	[11, 28]	url
Gnutella, Aug. 8, 2002	6,299	20,776	0.0455	0.1121	0.0109	0.0002	43	[24, 29]	url
JDK	6,434	53,658	0.0091	0.1173	0.6707	0.0047	44	[11]	url
AS Oregon	6,474	12,572	0.0356	0.1767	0.2522	0.0022	45	[30]	url
English	7,377	44,205	0.0114	0.1590	0.4085	0.0002	46	[1]	url
Gnutella, Aug. 9, 2002	8,104	26,008	0.0451	0.1099	0.0095	0.0002	47	[24, 29]	url
French	8,308	23,832	0.0236	0.1284	0.2138	0.0006	48	[1]	url
Hep-Th, 1993-2003	8,638	24,806	0.0755	0.1363	0.4816	0.0220	49	[24]	url
Gnutella, Aug. 6, 2002	8,717	31,525	0.0648	0.1005	0.0067	0.0002	50	[24, 29]	url
Gnutella, Aug. 5, 2002	8,842	31,837	0.0565	0.1017	0.0072	0.0002	51	[24, 29]	url
PGP	10,680	24,316	0.0642	0.1935	0.2659	0.0239	52	[31]	url
Gnutella, August 4 2002	10,876	39,994	0.0762	0.0942	0.0062	0.0002	53	[24, 29]	url
Hep-Ph, 1993-2003	11,204	117,619	0.0048	0.0565	0.6216	0.0176	54	[24]	url
Spanish	11,558	43,050	0.0127	0.2093	0.3764	0.0001	55	[1]	url
DBLP, citations	12,495	49,563	0.0325	0.0652	0.1178	0.0007	56	[11, 32]	url
Spanish	12,643	55,019	0.0119	0.2347	0.5042	0.0006	57	[11]	url
Cond-Mat, 1995-1999	13,861	44,619	0.0637	0.1649	0.6514	0.0402	58	[26]	url
Astrophysics	14,845	119,652	0.0182	0.0551	0.6696	0.0188	59	[26]	url
Google	15,763	148,585	0.0078	0.0636	0.5176	0.0041	60	[33]	url
AstroPhys, 1993-2003	17,903	196,972	0.0132	0.0323	0.6328	0.0113	61	[24]	url
Cond-Mat, 1993-2003	21,363	91,286	0.0367	0.0836	0.6417	0.0245	62	[24]	url
Gnutella, Aug. 25, 2002	22,663	54,693	0.1150	0.1265	0.0053	0.0003	63	[24, 29]	url
Internet	22,963	48,436	0.0193	0.1940	0.2304	0.0016	64		url
Thesaurus	23,132	297,094	0.0112	0.0174	0.0888	0.0000	65	[11, 34]	url
Cora	23,166	89,157	0.0454	0.1184	0.2660	0.0074	66	[11, 35]	url
Linux, mailing list	24,567	158,164	0.0052	0.2261	0.3391	0.0033	67	[11]	url
AS Caida	26,475	53,381	0.0210	0.1640	0.2082	0.0016	68	[30]	url
Gnutella, Aug. 24, 2002	26,498	65,359	0.1060	0.1174	0.0055	0.0002	69	[24, 29]	url
Hep-Th, citations	27,400	352,021	0.0110	0.0245	0.3139	0.0030	70	[11, 24]	url
Cond-Mat, 1995-2003	27,519	116,181	0.0342	0.0814	0.6546	0.0270	71	[26]	url
Digg	29,652	84,781	0.0413	0.0577	0.0054	0.0001	72	[11, 36]	url
Linux, soft.	30,817	213,208	0.0076	0.0457	0.1286	0.0023	73	[11]	url
Enron	33,696	180,811	0.0100	0.4460	0.5092	0.0162	74	[37]	url
Hep-Ph, citations	34,401	420,784	0.0158	0.0332	0.2856	0.0014	75	[11, 24]	url
Cond-Mat, 1995-2005	36,458	171,735	0.0256	0.0585	0.6566	0.0237	76	[26]	url
Gnutella, Aug. 30, 2002	36,646	88,303	0.0968	0.1122	0.0063	0.0002	77	[24, 29]	url
Slashdot	51,083	116,573	0.0262	0.0435	0.0201	0.0002	78	[11, 38]	url
Gnutella, Aug. 31, 2002	62,561	147,878	0.0956	0.1082	0.0055	0.0002	79	[24, 29]	url
Facebook	63,392	816,886	0.0086	0.0192	0.2218	0.0012	80	[39]	url

Supplementary Table 2: Continuation of Supplementary Table 1.

network	N	E	p_c	q_c	C	V	Fig.	Refs.	Url
`Epinions`	75,877	405,739	0.0062	0.0148	0.1378	0.0021	81	[11, 40]	url
`Slashdot zoo`	79,116	467,731	0.0088	0.0162	0.0584	0.0007	82	[11, 41]	url
`Flickr`	105,722	2,316,668	0.0142	0.0662	0.0884	0.0007	83	[11, 42]	url
`Wikipedia, edits`	113,123	2,025,910	0.0029	0.0085	0.3748	0.0017	84	[11, 43]	url
`Petster, cats`	148,826	5,447,464	0.0010	0.0303	0.3877	0.0003	85	[11]	url
`Gowalla`	196,591	950,327	0.0073	0.0310	0.2367	0.0052	86	[11, 44]	url
`Libimseti`	220,970	17,233,144	0.0011	0.0028	0.0429	0.0000	87	[11, 45, 46]	url
`EU email`	224,832	339,925	0.0119	0.5975	0.0791	0.0012	88	[11, 24]	url
`Web Stanford`	255,265	1,941,926	0.0598	0.1932	0.6189	0.0418	89	[37]	url
`Amazon, Mar. 2, 2003`	262,111	899,792	0.0940	0.1539	0.4198	0.0252	90	[47]	url
`DBLP, collaborations`	317,080	1,049,866	0.0337	0.0658	0.6324	0.0321	91	[11, 32]	url
`Web Notre Dame`	325,729	1,090,108	0.0847	0.2037	0.2346	0.0291	92	[48]	url
`MathSciNet`	332,689	820,644	0.0478	0.0805	0.4104	0.0186	93	[49]	url
`CiteSeer`	365,154	1,721,981	0.0250	0.0473	0.1832	0.0045	94	[11, 50]	url
`Zhishi`	372,840	2,318,025	0.0301	0.0738	0.2323	0.0081	95	[11, 51]	url
`Actor coll. net.`	374,511	15,014,839	0.0013	0.0047	0.7788	0.0071	96	[11, 52]	url
`Amazon, Mar. 12, 2003`	400,727	2,349,869	0.0401	0.0761	0.4022	0.0095	97	[47]	url
`Amazon, Jun. 6, 2003`	403,364	2,443,311	0.0364	0.0759	0.4177	0.0095	98	[47]	url
`Amazon, May 5, 2003`	410,236	2,439,437	0.0360	0.0759	0.4064	0.0094	99	[47]	url
`Petster, dogs`	426,485	8,543,321	0.0015	0.0380	0.1710	0.0002	100	[11]	url
`Road network PA`	1,087,562	1,541,514	0.6923	0.7571	0.0465	0.0385	101	[37]	url
`YouTube friend. net.`	1,134,890	2,987,624	0.0063	0.0171	0.0808	0.0021	102	[11, 53]	url
`Road network TX`	1,351,137	1,879,201	0.7362	0.7875	0.0470	0.0359	103	[37]	url
`AS Skitter`	1,694,616	11,094,209	0.0018	0.0130	0.2584	0.0044	104	[30]	url
`Road network CA`	1,957,027	2,760,388	0.6933	0.7582	0.0465	0.0382	105	[37]	url
`Wikipedia, pages`	2,070,367	42,336,614	0.0014	0.0046	0.2425	0.0002	106	[49]	url
`US Patents`	3,764,117	16,511,740	0.0290	0.0515	0.0758	0.0014	107	[11, 54]	url
`DBpedia`	3,915,921	12,577,253	0.0170	0.0380	0.3125	0.0032	108	[11, 55]	url
`LiveJournal`	5,189,809	48,688,097	0.0028	0.0116	0.2749	0.0033	109	[11, 56]	url

Supplementary Table 3: Continuation of Supplementary Tables 1 and 2.

Supplementary Note 1

Analysis of real networks

We report systematic comparisons between the bond and site percolation models in 109 real networks [58]. We consider graphs of heterogeneous nature, including biological, infrastructural, information, technological, social, and communication networks, and thus very diverse also in terms of structural properties (e.g., degree distribution and correlations, clustering coefficient, and diameter). In our numerical study, we reduce, if necessary, weighted and/or directed networks to their unweighted and undirected projections. We focus our attention only on their giant connected components. Details on the networks analyzed are reported in Supplementary Tables 1, 2 and 3. Numerical results have been obtained by averaging over $10,000$ independent realizations of the Newman-Ziff algorithm [59]. Best estimates of the bond percolation threshold p_c, and the site percolation threshold q_c are given by the respective values of the occupation probability where the susceptibility reaches its maximum. Finally, in Supplementary Fig. 110, we report a quantitative test of Eq. (5) of the main text.

Numerical results for the Erdős-Rényi model and random scale-free networks

In the following, we report the results of numerical simulations of the bond and site percolation models in various graph models. These results have been already discussed in the main text. Detailed descriptions are reported in the captions of the figures.

Supplementary References

[1] Milo, R. *et al.* Superfamilies of evolved and designed networks. *Science* **303**, 1538–1542 (2004).
[2] Zachary, W. W. An information flow model for conflict and fission in small groups. *J. Anthropol. Res.* 452–473 (1977).
[3] Lusseau, D. *et al.* The bottlenose dolphin community of doubtful sound features a large proportion of long-lasting associations. *Behav. Ecol. Sociobiol.* **54**, 396–405 (2003).
[4] Knuth, D. E., Knuth, D. E. & Knuth, D. E. *The Stanford GraphBase: A Platform For Combinatorial Computing*, vol. 37 (Addison-Wesley Reading, 1993).
[5] Mangan, S. & Alon, U. Structure and function of the feed-forward loop network motif. *Proc. Natl. Acad. Sci. USA* **100**, 11980–11985 (2003).
[6] Adamic, L. A. & Glance, N. The political blogosphere and the 2004 us election: divided they blog. In *Proceedings of the 3rd international workshop on Link discovery*, 36–43 (ACM, 2005).
[7] Newman, M. E. Finding community structure in networks using the eigenvectors of matrices. *Phys. Rev. E* **74**, 036104 (2006).
[8] Girvan, M. & Newman, M. E. Community structure in social and biological networks. *Proc. Natl. Acad. Sci. USA* **99**, 7821–7826 (2002).
[9] Fournet, J. & Barrat, A. Contact patterns among high school students. *PloS One* **9**, e107878 (2014).
[10] Ulanowicz, R., Bondavalli, C. & Egnotovich, M. Network analysis of trophic dynamics in south florida ecosystem, fy 97: The florida bay ecosystem. *Annual Report to the United States Geological Service Biological Resources Division Ref. No.[UMCES] CBL* 98–123 (1998).
[11] Kunegis, J. KONECT – The Koblenz Network Collection. In *Proc. Int. Conf. on World Wide Web Companion*, 1343–1350 (2013). URL `http://userpages.uni-koblenz.de/~kunegis/paper/kunegis-koblenz-network-collection.pdf`.
[12] Michalski, R., Palus, S. & Kazienko, P. Matching organizational structure and social network extracted from email communication. In *Lecture Notes in Business Information Processing*, vol. 87, 197–206 (Springer Berlin Heidelberg, 2011).
[13] Martinez, N. D. Artifacts or attributes? effects of resolution on the little rock lake food web. *Ecol. Monogr.* 367–392 (1991).
[14] Gleiser, P. M. & Danon, L. Community structure in jazz. *Adv. Complex Syst.* **6**, 565–573 (2003).
[15] Watts, D. J. & Strogatz, S. H. Collective dynamics of small-worldnetworks. *Nature* **393**, 440–442 (1998).
[16] Isella, L. *et al.* What's in a crowd? analysis of face-to-face behavioral networks. *J. Theor. Biol.* **271**, 166–180 (2011).
[17] Colizza, V., Pastor-Satorras, R. & Vespignani, A. Reaction–diffusion processes and metapopulation models in heterogeneous networks. *Nature Phys.* **3**, 276–282 (2007).
[18] Milo, R. *et al.* Network motifs: simple building blocks of complex networks. *Science* **298**, 824–827 (2002).
[19] Guimera, R., Danon, L., Diaz-Guilera, A., Giralt, F. & Arenas, A. Self-similar community structure in a network of human interactions. *Phys. Rev. E* **68**, 065103 (2003).
[20] Jeong, H., Mason, S. P., Barabási, A.-L. & Oltvai, Z. N. Lethality and centrality in protein networks. *Nature* **411**, 41–42 (2001).
[21] Opsahl, T. & Panzarasa, P. Clustering in weighted networks. *Soc. Networks* **31**, 155–163 (2009).
[22] Bu, D. *et al.* Topological structure analysis of the protein–protein interaction network in budding yeast. *Nucleic Acids Res.* **31**, 2443–2450 (2003).
[23] Opsahl, T., Agneessens, F. & Skvoretz, J. Node centrality in weighted networks: Generalizing degree and shortest paths. *Soc. Networks* **32**, 245–251 (2010).
[24] Leskovec, J., Kleinberg, J. & Faloutsos, C. Graph evolution: densification and shrinking diameters. In *ACM Transactions on Knowledge Discovery from Data (TKDD)*, vol. 1, 2 (ACM, 2007).
[25] Radicchi, F. Who is the best player ever? a complex network analysis of the history of professional tennis. *PloS One* **6**, e17249 (2011).
[26] Newman, M. E. The structure of scientific collaboration networks. *Proc. Natl. Acad. Sci. USA* **98**, 404–409 (2001).
[27] Joshi-Tope, G. *et al.* Reactome: a knowledgebase of biological pathways. *Nucleic Acids Res.* **33**, D428–D432 (2005).
[28] Šubelj, L. & Bajec, M. Software systems through complex networks science: Review, analysis and applications. In *Proceedings of the First International Workshop on Software Mining*, 9–16 (ACM, 2012).
[29] Ripeanu, M., Foster, I. & Iamnitchi, A. Mapping the gnutella network: properties of large-scale peer-to-peer systems and implications for system design. *IEEE Internet Comput.* **6**, 2002 (2002).
[30] Leskovec, J., Kleinberg, J. & Faloutsos, C. Graphs over time: densification laws, shrinking diameters and possible explanations. In *Proceedings of the eleventh ACM SIGKDD international conference on Knowledge discovery in data mining*, 177–187 (ACM, 2005).
[31] Boguñá, M., Pastor-Satorras, R., Díaz-Guilera, A. & Arenas, A. Models of social networks based on social distance attachment. *Phys. Rev. E* **70**, 056122 (2004).
[32] Ley, M. The dblp computer science bibliography: Evolution, research issues, perspectives. In *String Processing and Information Retrieval*, 1–10 (Springer, 2002).
[33] Palla, G., Farkas, I. J., Pollner, P., Derenyi, I. & Vicsek, T. Directed network modules. *New J. Phys.* **9**, 186 (2007).
[34] Kiss, G. R., Armstrong, C., Milroy, R. & Piper, J. An associative thesaurus of english and its computer analysis. *The Computer and Literary Studies* 153–165 (1973).
[35] Šubelj, L. & Bajec, M. Model of complex networks based on citation dynamics. In *Proceedings of the 22nd international conference on World Wide Web companion*, 527–530 (International World Wide Web Conferences Steering Committee,

2013).
[36] De Choudhury, M., Sundaram, H., John, A. & Seligmann, D. D. Social synchrony: Predicting mimicry of user actions in online social media. In *Computational Science and Engineering, 2009. CSE'09. International Conference on*, vol. 4, 151–158 (IEEE, 2009).
[37] Leskovec, J., Lang, K. J., Dasgupta, A. & Mahoney, M. W. Community structure in large networks: Natural cluster sizes and the absence of large well-defined clusters. *Internet Mathematics* **6**, 29–123 (2009).
[38] Gómez, V., Kaltenbrunner, A. & López, V. Statistical analysis of the social network and discussion threads in slashdot. In *Proceedings of the 17th international conference on World Wide Web*, 645–654 (ACM, 2008).
[39] Viswanath, B., Mislove, A., Cha, M. & Gummadi, K. P. On the evolution of user interaction in facebook. In *Proceedings of the 2nd ACM workshop on Online social networks*, 37–42 (ACM, 2009).
[40] Richardson, M., Agrawal, R. & Domingos, P. Trust management for the semantic web. In *The Semantic Web-ISWC 2003*, 351–368 (Springer, 2003).
[41] Kunegis, J., Lommatzsch, A. & Bauckhage, C. The slashdot zoo: mining a social network with negative edges. In *Proceedings of the 18th international conference on World Wide Web*, 741–750 (ACM, 2009).
[42] McAuley, J. & Leskovec, J. Learning to discover social circles in ego networks. In *Advances in Neural Information Processing Systems*, 548–556 (2012).
[43] Brandes, U. & Lerner, J. Structural similarity: spectral methods for relaxed blockmodeling. *J. Classif.* **27**, 279–306 (2010).
[44] Cho, E., Myers, S. A. & Leskovec, J. Friendship and mobility: user movement in location-based social networks. In *Proceedings of the 17th ACM SIGKDD international conference on Knowledge discovery and data mining*, 1082–1090 (ACM, 2011).
[45] Brozovsky, L. & Petricek, V. Recommender system for online dating service. *arXiv preprint cs/0703042* (2007).
[46] Kunegis, J., Gröner, G. & Gottron, T. Online dating recommender systems: The split-complex number approach. In *Proceedings of the 4th ACM RecSys workshop on Recommender systems and the social web*, 37–44 (ACM, 2012).
[47] Leskovec, J., Adamic, L. A. & Huberman, B. A. The dynamics of viral marketing. *ACM Transactions on the Web (TWEB)* **1**, 5 (2007).
[48] Albert, R., Jeong, H. & Barabási, A.-L. Internet: Diameter of the world-wide web. *Nature* **401**, 130–131 (1999).
[49] Palla, G., Farkas, I. J., Pollner, P., Derényi, I. & Vicsek, T. Fundamental statistical features and self-similar properties of tagged networks. *New J. Phys.* **10**, 123026 (2008).
[50] Bollacker, K. D., Lawrence, S. & Giles, C. L. Citeseer: An autonomous web agent for automatic retrieval and identification of interesting publications. In *Proceedings of the second international conference on Autonomous agents*, 116–123 (ACM, 1998).
[51] Niu, X. *et al.* Zhishi. me-weaving chinese linking open data. In *The Semantic Web–ISWC 2011*, 205–220 (Springer, 2011).
[52] Barabási, A.-L. & Albert, R. Emergence of scaling in random networks. *Science* **286**, 509–512 (1999).
[53] Yang, J. & Leskovec, J. Defining and Evaluating Network Communities based on Ground-truth. In *Proceedings of the ACM SIGKDD Workshop on Mining Data Semantics*, 3 (ACM, 2012).
[54] Hall, B. H., Jaffe, A. B. & Trajtenberg, M. The nber patent citation data file: Lessons, insights and methodological tools. Tech. Rep., National Bureau of Economic Research (2001).
[55] Auer, S. *et al. Dbpedia: A nucleus for a web of open data* (Springer, 2007).
[56] Mislove, A., Marcon, M., Gummadi, K. P., Druschel, P. & Bhattacharjee, B. Measurement and analysis of online social networks. In *Proceedings of the 7th ACM SIGCOMM conference on Internet measurement*, 29–42 (ACM, 2007).
[57] Newman, M. E. Random graphs with clustering. *Phys. Rev. Lett.* **103**, 058701 (2009).
[58] Radicchi, F. Predicting percolation thresholds in networks. *Phys. Rev. E* **91**, 010801 (2015).
[59] Newman, M. E. J. & Ziff, R. Efficient monte carlo algorithm and high-precision results for percolation. *Phys. Rev. Lett.* **85**, 4104 (2000).
